# Maspardin/SPG21 controls lysosome motility and TFEB phosphorylation through RAB7 positioning

**DOI:** 10.1083/jcb.202501135

**Published:** 2025-12-16

**Authors:** Thomas Jacqmin, Florentine Gilis, Martine Albert, Jean-François Gaussin, Michel Jadot, Marielle Boonen

**Affiliations:** 1 Laboratory of Intracellular Trafficking Biology, URPhyM, NARILIS, University of Namur, Namur, Belgium; 2 Laboratory of Physiological Chemistry, URPhyM, NARILIS, University of Namur, Namur, Belgium

## Abstract

Spastic paraplegia 21 is a neurodegenerative disease characterized by the degeneration of corticospinal axons. It is caused by mutations in the *SPG21* gene, which encodes maspardin, a cytosolic protein of unknown function that associates with the late endosomal/lysosomal membrane. Intriguingly, we found that the phosphorylation level of the transcription factor EB (TFEB), a master regulator of the CLEAR gene network, is decreased in *SPG21* knockout cells, leading to TFEB nuclear translocation. Our investigations revealed that the Rag-mediated presentation of TFEB to the mTOR kinase and its subsequent phosphorylation is disturbed by a delocalization of the RAB7 GTPase, a maspardin-binding partner, from retromer-positive late endosomes to lysosomes*.* This redistribution decreases the interaction between RAB7 and its GTPase-activating protein (GAP), TBC1D5. Consequently, RAB7 remains primarily GTP-bound, recruiting more FYCO1 to lysosomes and promoting the anterograde movement of these organelles along microtubules. These findings identify maspardin as a newly discovered RAB7 effector and shed light on several consequences of its deficiency.

## Teaser

Jacqmin et al. report that the loss of maspardin, which is deficient in spastic paraplegia 21, results in RAB7 hyperactivation due to mislocalization. This abnormal RAB7 localization leads to defects in lysosome trafficking and disruption of the mTOR–TFEB pathway.

## Introduction

Mutations in the *SPG21* gene underlie hereditary spastic paraplegia (SPG) type 21 (also known as Mast syndrome). This is a complex form of SPG characterized by motor coordination defects due to axonal degeneration of corticospinal neurons and associated with dementia, cerebellar, and extrapyramidal abnormalities and, in some cases, seizures (Harding, 1983; Simpson et al., 2003). Axonal branching of cortical neurons isolated from *spg21* -/- mice has been found to be altered (although two independent studies reported opposite results) (Soderblom et al., 2010; Davenport et al., 2016). However, the precise mechanism(s) underlying these pathological manifestations remain(s) unclear, as the function of the 308 amino acid protein encoded by human *SPG21* (or *spg21* in mice), called maspardin, has not yet been identified.

Previous immunofluorescence studies have documented that maspardin partially colocalizes with markers of late endosomes and lysosomes in HeLa cells (Hanna and Blackstone, 2009). Furthermore, by studying the distribution of the rat liver proteome between the eight major subcellular compartments using a proteomic profiling approach (Jadot et al., 2017), we remarked that maspardin exhibits an intracellular distribution very similar to those of several other SPG-associated proteins, including strumpellin (SPG8), spatacsin (SPG11), spastizin (SPG15), and AP5Z1 (SPG48). All these proteins reside both in the cytosol and at the late endosomal/lysosomal membrane.

The adaptor protein AP5, spatacsin, and spastizin have been reported to assemble into a complex on late endosomes and contribute to the retrograde trafficking of several proteins from this site to the trans-Golgi network (Hirst et al., 2013). They are also implicated in the process of lysosome reformation by tubulation, for instance, at the end of the autophagy process (Chang et al., 2014; Hirst et al., 2015; Boutry et al., 2019; Khundadze et al., 2019; Vantaggiato et al., 2019). As for strumpellin, it is required for the fission of tubules that bud from endosomes and act as carriers toward other organelles (Harbour et al., 2010; Allison et al., 2017).

Intriguingly, it has been reported that the transcription factor EB (TFEB) is activated in spatacsin (SPG11)-deficient cells, leading to a decrease in lysosomal tubulation processes by a mechanism that is not yet fully understood (Boutry et al., 2019). TFEB is known to regulate the expression of lysosomal and autophagy genes when it is translocated to the nucleus (Sardiello et al. 2009). This translocation is induced by a decrease in TFEB phosphorylation by the mTOR kinase when the mTOR complex 1 (mTORC1) dissociates from the lysosomal membrane (e.g., upon nutrient starvation or lysosomal stress), and/or when TFEB is dephosphorylated by the cytosolic calcineurin phosphatase (Medina et al. 2015). In spatacsin-deficient cells, mTOR activity was reported to be unaltered, but cholesterol depletion at the plasma membrane due to cholesterol accumulation in lysosomes, resulted in calcium entry and calcineurin hyperactivation, which favored TFEB dephosphorylation and nuclear translocation (Boutry et al., 2019).

Interestingly, the small GTPase RAB7 (Ras-related protein RAB7) has been identified as a binding partner of maspardin (SPG21) in HEK293T cells (McCray et al., 2010), and it has been reported that delocalization of RAB7 to amino acid–sensing domains in the lysosomal membrane (where mTOR is active under basal/nutrient-rich conditions) prevents recruitment of Rag GTPases to these domains, thereby disrupting mTOR function (Kvainickas et al., 2019). Rag A/B are required for mTOR binding to the lysosomal membrane, whereas RagC/D recruit and present TFEB to mTOR but are not required for the presentation of other mTOR substrates such as the kinase S6K (Napolitano et al., 2020, 2022). Taken together, the late endosomal/lysosomal localization of maspardin (Hanna and Blackstone, 2009; Jadot et al., 2017), its reported interaction with RAB7 (McCray et al., 2010), and the discovery that TFEB is activated in other lysosome-associated SPG models (Boutry et al., 2019) led us to wonder whether maspardin might be a novel RAB7 effector and modulate the mTOR–TFEB pathway via this GTPase. It is worth noting that mutations in the *RAB7* gene underlie the neurodegenerative disease Charcot-Marie-Tooth type 2B and cause inhibition of axon development as well as neurite outgrowth defects (BasuRay et al., 2013). Several of these mutations increase the membrane residence time of RAB7 by favoring its GTP-bound state (De Luca et al., 2008; Spinosa et al., 2008). Moreover, RAB7-positive vesicles exhibit altered motility in Charcot-Marie-Tooth type 2B models, likely contributing to the impaired neurite development as this process relies on endosomal and lysosomal trafficking for membrane extension and protein delivery (Zhang et al., 2013; Mulligan and Winckler, 2023). Therefore, in addition to testing the effect of a maspardin deficiency on the mTOR–TFEB pathway in the context of a putative RAB7 deregulation, we assessed its putative consequences on lysosome motility.

## Results

### TFEB phosphorylation level and localization are altered in *SPG21* KO HeLa cells

**Figure 1.**
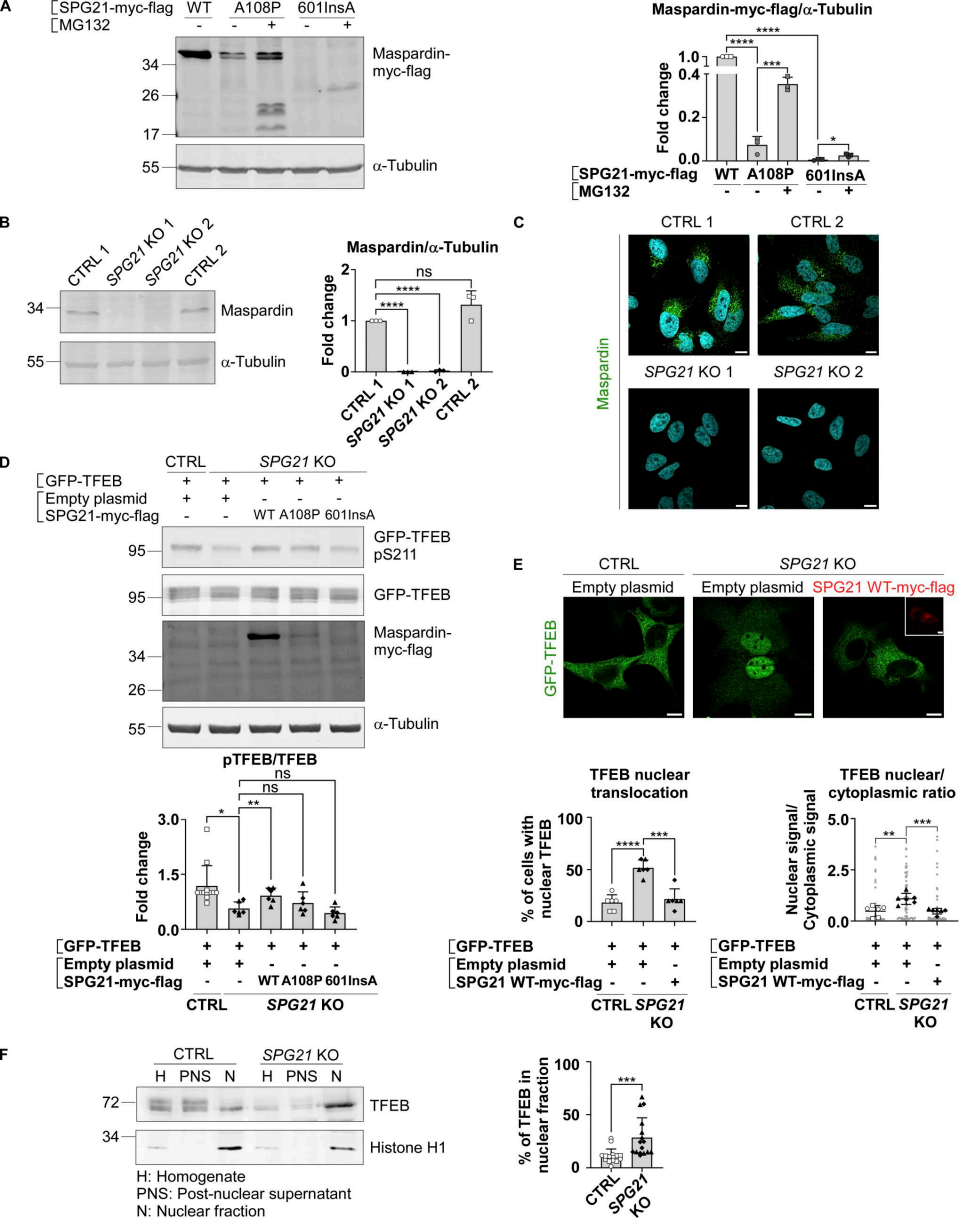
**Pathogenic mutations in *SPG21* cause maspardin misfolding, and maspardin deficiency decreases pTFEB/TFEB ratio in HeLa cells. (A)** HeLa cells were transfected for 48 h with either SPG21 WT-myc-flag, SPG21 A108P-myc-flag, or SPG21 601insA-myc-flag constructs and treated with DMSO (vehicle) or 1 µM of MG132 during the last 16 h. Maspardin was detected by western blotting using an anti-myc antibody (at ∼38 kDa for the WT and p.A108P mutants and at ∼28 kDa for the c.601insA mutant). α-Tubulin detection was used as a loading control. Fold changes of maspardin/α-tubulin signal are shown in the graph on the right. Mean ± SD. *n* = 3 independent experiments. Two-tailed unpaired *t* test. *P < 0.05; ***P < 0.001; ****P < 0.0001. **(B)** Western blotting detection of endogenous maspardin in two control (CTRL) and two newly-generated *SPG21* KO HeLa clones. The graph shows fold changes of maspardin/α-tubulin signal. Mean ± SD. *n* = 3 independent experiments, including two CTRL and two KO clones (open circles: CTRL1; open squares: CTRL2; black triangles: KO1; black diamonds: KO2). Two-tailed unpaired *t* test. ns, nonsignificant; ****P < 0.0001. **(C)** Immunofluorescence detection of endogenous maspardin (green) in CTRL and *SPG21* KO clones. Nuclei were stained with DAPI. One representative set of *n* = 3 independent experiments, including two CTRL and two KO clones. Scale bar = 10 µm. **(D)** Western blotting analysis and quantification of the pTFEB(Ser211)/TFEB ratio in CTRL and *SPG21* KO HeLa cells 48 h after transfection of a GFP-TFEB construct with an empty plasmid or a SPG21-myc-flag construct (WT, p.A108P, or c.601insA). Overexpressed proteins were detected with an anti-myc antibody (N.B. due to its extensive degradation, the 601insA mutant is not detected under these basal conditions). *n* = 6 for the KO cells (3 independent experiments, including two different KO clones) and *n* = 12 for the CTRL cells (each of the 6 blots contained the two CTRL clones co-transfected with GFP-TFEB and an empty plasmid. Only one is shown in the figure). Mean fold change of pTFEB/TFEB ratio ± SD. Two-tailed unpaired *t* test. *P < 0.05; **P < 0.01. **(E)** Analysis of GFP-TFEB distribution in CTRL and *SPG21* KO HeLa by confocal microscopy 72 h after transfection with an empty vector or SPG21 WT-myc-flag. Scale bar = 10 µm. The graphs show the mean percentage of cells (± SD) with a predominant nuclear localization for TFEB (left) or TFEB nuclear/cytoplasmic signal ratio (right). *n* = 6 (three independent experiments, including each two different CTRL and two different KO clones). 10 cells were analyzed per clone in each experiment. These values are shown in light gray, while their averages are shown as follows: open circles: CTRL1; open squares: CTRL2; black triangles: KO1; black diamonds: KO2. Two-tailed unpaired *t* test. **P < 0.01; ***P < 0.001; ****P < 0.0001. **(F)** Analysis of endogenous TFEB nuclear/cytoplasmic distribution in CTRL or *SPG21* KO HeLa cells. The cells were homogenized and fractionated into a nuclear (N) fraction and a postnuclear supernatant (PNS) fraction. Equal amount of proteins from the cell homogenates (H), N, and PNS fractions were analyzed by western blotting using an anti-TFEB antibody. Histone H1 (nuclear marker) was used as a control. *n* = 16 (8 independent experiments, including two CTRL and two KO clones). The graph shows the mean percentage of TFEB in the N fraction relative to total cellular signal ± SD. Two-tailed paired *t* test. ***P < 0.001. Source data are available for this figure: SourceData F1.

Since the first case of SPG 21 was described, several mutations in the *SPG21* gene have been reported to cause the disease (Simpson et al., 2003; Ishiura et al., 2014; Amprosi et al., 2021). We first investigated the effect on maspardin expression of two common mutations, c.601insA and p.A108P, resulting in a premature stop codon and amino acid substitution, respectively (Simpson et al., 2003; Ishiura et al., 2014). When we introduced these mutations into a SPG21-myc-flag cDNA and transfected these constructs into HeLa cells, we found that they reduced maspardin expression level by >90%, as assessed by western blotting (Fig. 1 A, P < 0.0001). After an overnight treatment of the cells with MG132, an inhibitor of the proteasome, the signals increased by four- to fivefold for the full-length p.A108P mutant (detected at ∼38 kDa) and truncated c.601insA mutant (detected around 28 kDa) (Fig. 1 A, P < 0.05 and P < 0.001, respectively). We infer from these data that maspardin expression in cells from patients with SPG 21 (an autosomal recessive disease) is likely to be largely reduced, at least in part, due to protein misfolding.

Thus, we decided to engineer an *SPG21* KO HeLa cell model by a CRISPR–Cas9 genome editing method to search for the molecular function of maspardin. Two control clones and two KO clones, each generated using a different guide RNA, were selected for our studies. The loss of maspardin expression was validated in western blotting (Fig. 1 B) and immunofluorescence analyses (Fig. 1 C). All clones were analyzed separately in the experiments presented hereafter, with the results pooled in graphical representations and a representative set from one of the clones shown in the figure panels.

Interestingly, when we expressed a GFP-tagged TFEB construct in these cells, followed by western blotting detection of total TFEB forms or of its phosphorylated form at serine 211 (a site recognized by the mTOR kinase), we detected a decrease in total signal in *SPG21* KO cells compared with control cells, mainly due to a lower level of phospho-TFEB forms (pTFEB) (Fig. 1 D). An overall twofold decrease in the pTFEB/TFEB ratio was quantified in the KO cells (P < 0.05), which could be corrected by re-expression of WT maspardin, but not by the pathogenic c.601insA mutant (Fig. 1 D). Although it did not reach the statistical threshold, the pTFEB/TFEB ratio was slightly higher in KO cells transfected with the p.A108P mutant than in mock-transfected cells (Fig. 1 D, P = 0.32) and cells expressing the c.601insA mutant (P = 0.078). These observations raise the possibility that this mutant may retain partial functionality, which would be consistent with the milder, late-onset clinical phenotype reported in patients carrying this mutation (Ishiura et al., 2014).

We then analyzed the subcellular localization of GFP-TFEB by fluorescence microscopy, which revealed an increase of its presence in the nucleus of *SPG21* KO cells (P < 0.01) that could also be corrected by re-expression of WT maspardin (P < 0.001), as assessed by two different quantification methods (measurement of the nuclear/cytoplasmic fluorescence ratio and of the percentage of cells with a predominant nuclear localization of GFP-TFEB, Fig. 1 E). In addition, we analyzed endogenous TFEB distribution by western blotting after fractionation of control and *SPG21* KO cells into nuclear and postnuclear fractions (Fig. 1 F). Only total TFEB was analyzed, as endogenous pTFEB was below the detection limit. Consistent with previous findings using the GFP-tagged TFEB construct, we detected an increase in endogenous TFEB signal, more specifically of its lower molecular weight (MW) form, in the nuclear fraction of KO cells (P < 0.001). The higher MW form, likely representing the phosphorylated form of TFEB, was mainly detected in the control cells and remained in the postnuclear fraction, consistent with its trapping in the cytosol by association with 14-3–3 proteins (Roczniak-Ferguson et al., 2012). Taken together, these data suggest that a maspardin deficiency affects one or several molecule(s) that control the phosphorylation level of TFEB and thus its nuclear translocation.

### Reduced pTFEB/TFEB ratio in *SPG21* KO cells results from decreased phosphorylation of TFEB by the mTOR kinase

**Figure S1.**
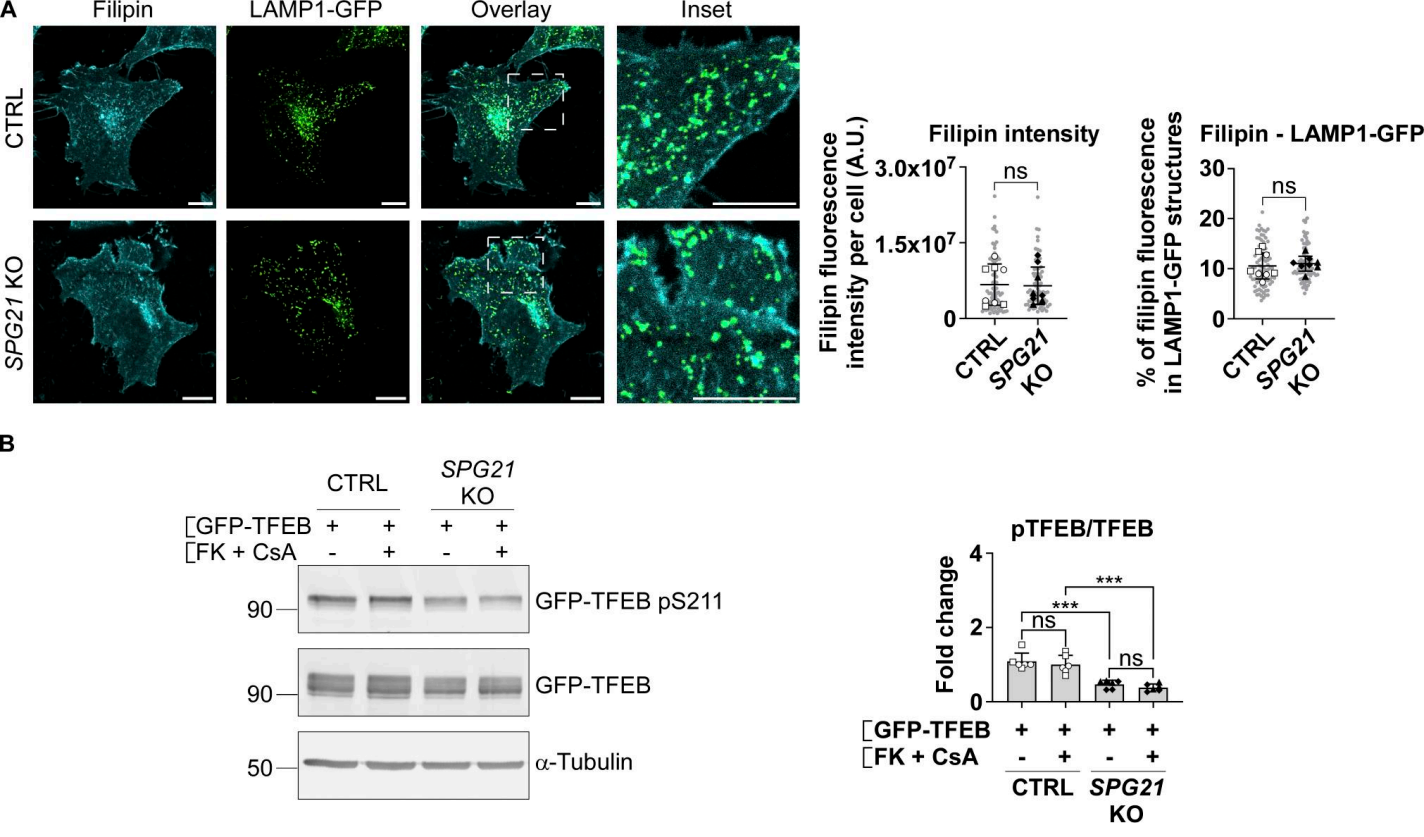
**Calcineurin hyperactivation is not responsible for the decreased pTFEB/TFEB ratio in *SPG21* KO HeLa cells. (A)** Cholesterol staining (filipin) in CTRL and *SPG21* KO HeLa cells 48 h after transfection with a LAMP1-GFP plasmid. Graphs show quantifications of filipin fluorescence intensity per cell (left graph) and the relative fluorescence intensity of filipin in LAMP1-GFP–positive structures (right graph). *n* = 8 (four independent experiments including two CTRL and two KO clones). 10 cells were analyzed per clone in each experiment. These values are shown in light gray. Their averages are shown as follows: open circles: CTRL1; open squares: CTRL2; black triangles: KO1; black diamonds: KO2. Mean ± SD. Two-tailed unpaired *t* test. ns, nonsignificant, A.U., arbitrary unit. **(B)** CTRL and *SPG21* KO HeLa cells were transfected with GFP-TFEB for 24 h and treated during the last 3 h with 5 µM of FK506 (FK) and 10 µM of cyclosporin A (CsA) or with DMSO (vehicle). pTFEB/TFEB ratio was then analyzed by western blotting. α-Tubulin was used as a loading control. *n* = 6 (three independent experiments including two CTRL and two KO clones). Mean ± SD. Two-tailed unpaired *t* test. ns, nonsignificant; ***P < 0.001. Source data are available for this figure: SourceData FS1.

In spatacsin/SPG11-deficient cells, F. Darios’s group linked TFEB nuclear translocation to an accumulation of cholesterol in lysosomes and a concomitant depletion from the plasma membrane, leading to calcium entry into the cells and hyperactivation of calcineurin (a phosphatase that acts on TFEB) (Boutry et al., 2019). Therefore, to begin the search for the cause of decreased pTFEB level in our model, we analyzed cholesterol accumulation in lysosomes of control and *SPG21* KO cells by measuring the fluorescence intensity of filipin signal (which stains free cholesterol) in LAMP1-GFP–positive structures. LAMP1 is a marker of late endosomes and lysosomes. However, no accumulation of cholesterol was detected in these organelles (Fig. S1 A). Moreover, inhibition of calcineurin using a combination of FK506 and cyclosporin A (two known inhibitors of this phosphatase) did not rescue TFEB phosphorylation level in the KO cells (Fig. S1 B). Taken together, these results suggest that a different mechanism underlies TFEB translocation to the nucleus in *SPG21* KO cells.

**Figure 2.**
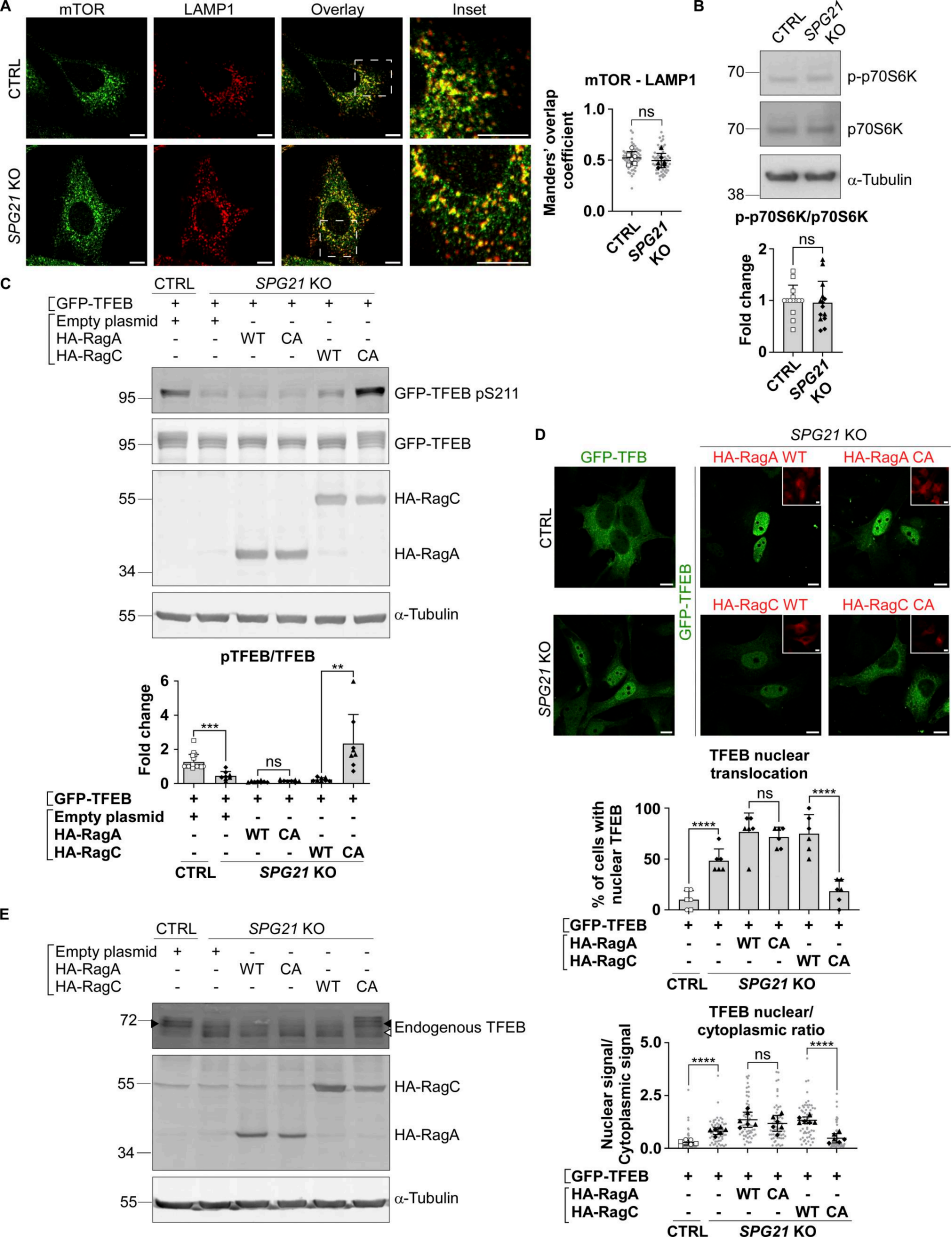
**The decreased pTFEB/TFEB ratio in *SPG21* KO HeLa cells is due to defective TFEB presentation to mTORC1 by RagC. (A)** Colocalization analysis between mTOR (green) and LAMP1 (red) in CTRL and *SPG21* KO HeLa cells. Scale bar = 10 µm. *n* = 6 (three independent experiments each, including two different CTRL and two different KO clones). 10 cells were analyzed per clone in each experiment. These values are shown in light gray. Their averages are shown as follows: open circles: CTRL1; open squares: CTRL2; black triangles: KO1; black diamonds: KO2. Data are represented as mean Manders’ coefficients ± SD. Two-tailed unpaired *t* test. ns, nonsignificant. **(B)** Western blotting detection of the phosphorylated form (on threonine 389) and total form of p70S6K in CTRL and *SPG21* KO HeLa cells. α-Tubulin is shown as a loading control. The graph shows the quantification of the mean fold change ratio of p-p70S6K/p70S6K ± SD in 7 independent experiments for both CTRL and both KO clones (*n* = 14). Two-tailed unpaired *t* test. ns, nonsignificant. **(C)** Western blotting analysis of pTFEB/TFEB ratio in CTRL and *SPG21* KO cells 24 h after transfection of GFP-TFEB with WT or CA forms of HA-RagA or HA-RagC constructs. Rag proteins were detected with an anti-HA antibody, and α-tubulin is shown as a loading control. *n* = 8 for the KO cells (four independent experiments, including two different KO clones and *n* = 16 for the CTRL cells [each of the eight blots contained the two CTRL co-transfected with GFP-TFEB and an empty plasmid. One of them is shown in the figure]). Mean fold change of pTFEB/TFEB ratio ± SD. Two-tailed unpaired *t* test. ns, nonsignificant; **P < 0.01; ***P < 0.001. **(D)** Confocal microscopy detection of GFP-TFEB in CTRL and KO clones after co-transfection with WT or CA HA-RagA or HA-RagC constructs (red). Scale bar = 10 µm. The graphs show the quantification of the percentage of cells with predominant nuclear signal for TFEB (top graph) or the TFEB nuclear/cytoplasmic signal ratio (bottom graph). Three independent experiments that included two CTRL and two KO clones (*n* = 6). 10 cells were analyzed per clone in each biological replicate. Mean ± SD. Two-tailed unpaired *t* test. ns, nonsignificant; ****P < 0.0001. **(E)** Western blotting detection of endogenously expressed TFEB in CTRL and *SPG21* KO cells (two clones each) transfected for 48 h with WT or CA HA-RagA or C. One representative set of four independent experiments is shown. The black arrowhead indicates the higher MW forms of TFEB (pTFEB). The white arrowhead indicates lower MW forms of TFEB (non-phosphorylated). Source data are available for this figure: SourceData F2.

It is well known that TFEB is a substrate of the mTOR kinase, which is active when localized to lysosomes (Medina et al., 2015). We therefore tested whether mTOR localization is altered in *SPG21* KO cells using confocal microscopy, but we did not observe any difference in the level of colocalization between this kinase and LAMP1 in maspardin-deficient cells compared with control cells (Fig. 2 A). We also tested mTOR activity by measuring the phosphorylation level of another of its targets, the kinase p70S6K, but found no change in the p-p70S6K/p70S6K ratio in *SPG21* KO HeLa cells (Fig. 2 B).

**Figure S2.**
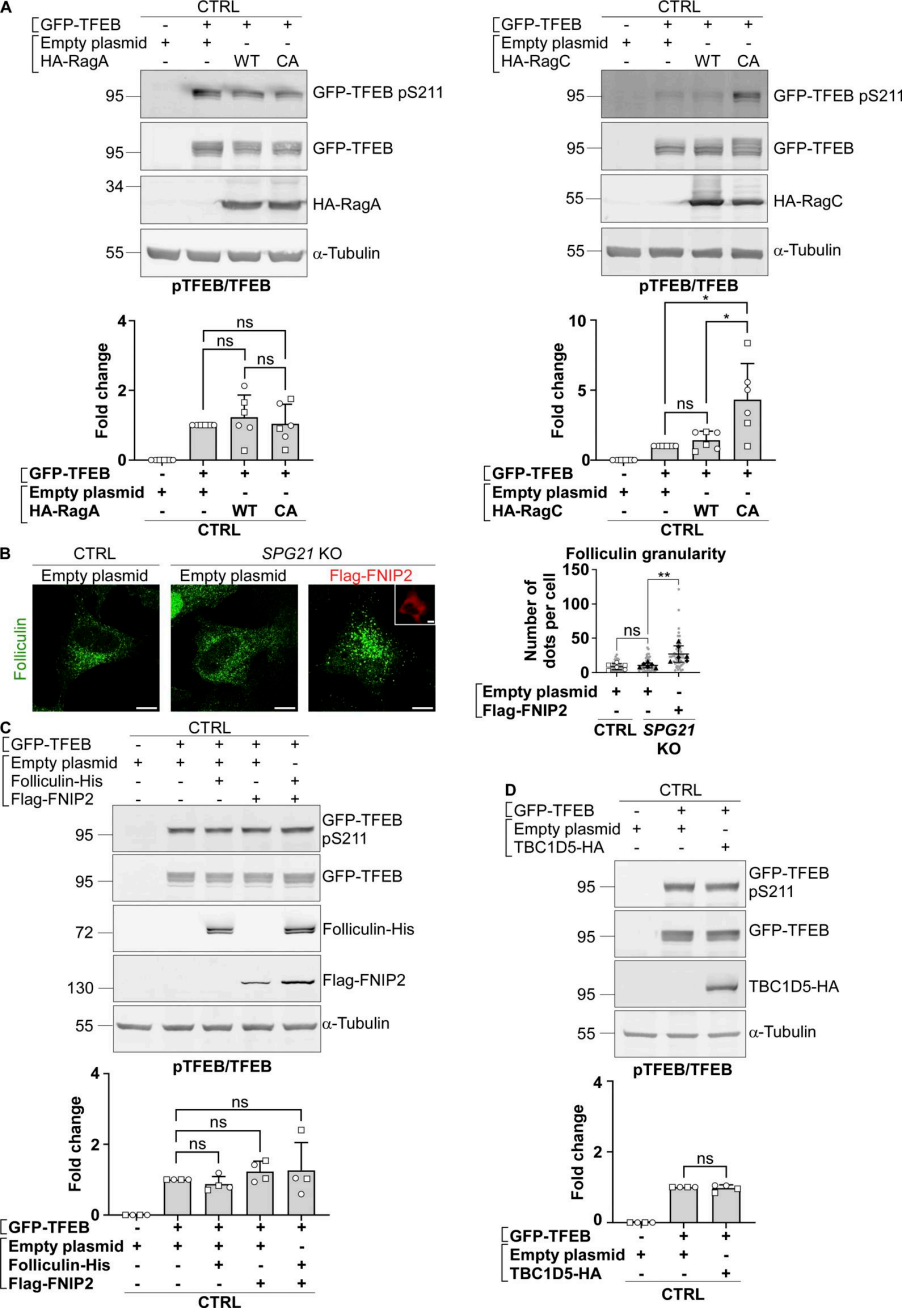
**pTFEB/TEFB ratios in control cells after transfection of RagA, RagC, folliculin/FNIP, or TBC1D5 constructs, and analysis of the localization of endogenous folliculin when Flag-FNIP2 is overexpressed. (A, C and D)** Western blotting analysis of pTFEB/TFEB ratio in CTRL HeLa cells 24 h (RagA/RagC) or 48 h (folliculin/FNIP; TBC1D5) after transfection of an empty plasmid or GFP-TFEB with either an empty plasmid, HA-RagA (WT or CA), HA-RagC (WT or CA), FLCN-His, and/or Flag-FNIP or TBC1D5-HA constructs. α-Tubulin was used as a loading control. The graphs show the mean fold change in pTFEB/TFEB ratio ± SD. For RagA and RagC overexpression experiments, *n* = 6 (three independent experiments with two different CTRL clones (open circles: CTRL1; open squares: CTRL2); for folliculin/FNIP and TBC1D5 overexpression experiments, *n* = 4 (two independent experiments with two different CTRL clones). Two-tailed unpaired *t* test. ns, nonsignificant; *P < 0.05. **(B)** Analysis of endogenous folliculin (green) distribution by confocal microscopy in CTRL and *SPG21* KO HeLa 48 h after the overexpression of a Flag-FNIP2 plasmid (red) or an empty plasmid. Scale bar = 10 µm. The graph shows the quantifications of folliculin granularity. *n* = 6 (three independent experiments, including two CTRL and two KO clones). 10 cells were analyzed per clone in each experiment. These values are shown in light gray. Their averages are shown as follows: open circles: CTRL1; open squares: CTRL2; black triangles: KO1; black diamonds: KO2. Mean ± SD. Two-tailed unpaired *t* test. ns, nonsignificant. **P < 0.01. Source data are available for this figure: SourceData FS2.

However, it is worth noting that the recruitment of TFEB and p70S6K to the lysosomal mTORC1 platform, conditioning their subsequent phosphorylation by the mTOR kinase, is mediated by different mechanisms (Napolitano et al., 2020; Napolitano et al., 2022). p70S6K contains a TOR signaling motif that allows direct recruitment by the Raptor subunit of mTORC1, whereas TFEB instead contains a Rag-binding region. It requires a RagA/B-GTP:RagC/D-GDP complex to be recruited and phosphorylated by mTOR. Therefore, we decided to investigate whether the decreased pTFEB/TFEB ratio in *SPG21* KO cells could result from defective presentation of TFEB to mTOR by Rag GTPases. To do so, a WT or a constitutively active (CA) form of RagA (GTP-bound) or RagC (GDP-bound) was co-transfected with GFP-TFEB in maspardin-deficient cells. Interestingly, GFP-TFEB phosphorylation level in *SPG21* KO cells was rescued by overexpression of the CA form of RagC (Fig. 2 C, P < 0.01 relative to WT RagC transfection). CA RagC also slightly increased phospho-GFP-TFEB levels in control cells (Fig. S2 A). By contrast, the CA form of RagA had no effect in control or KO cells (Fig. 2 C and Fig. S2 A), which is consistent with the reports of a predominant effect of RagC in controlling TFEB phosphorylation by mTOR (Alesi et al., 2021; Li et al., 2022). Indeed, GFP-TFEB was also relocalized to the cytoplasm of *SPG21* KO cells following CA RagC overexpression, but not CA RagA (Fig. 2 D, P < 0.0001 and ns, respectively). Furthermore, only the CA RagC construct rescued endogenous TFEB phosphorylation, as evidenced by the slight slowing down of its electrophoretic mobility in *SPG21* KO cells transfected with this construct (Fig. 2 E).

These findings support that maspardin deficiency disrupts the RagC-mediated presentation of TFEB to mTOR and suggest that the localization of RagC to the lysosomal membrane and/or its GDP/GTP ratio (active/inactive form ratio) is reduced in the absence of maspardin.

**Figure 3.**
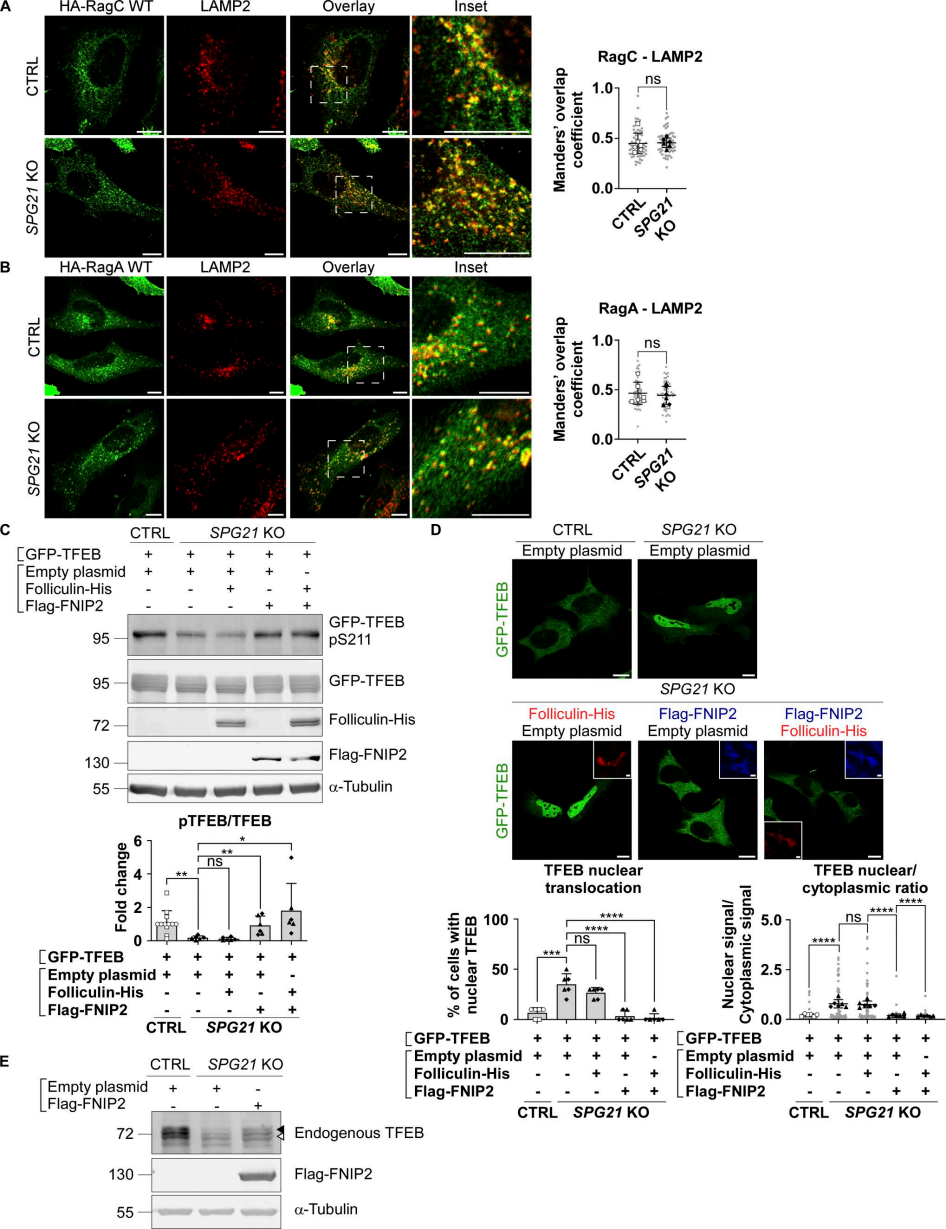
**Activation of RagC by transfection of its GAP FLCN-FNIP2 rescues TFEB phosphorylation and localization. (A)** Colocalization analysis between HA-RagC (green) and LAMP2 (red) 24 h after transfection in CTRL and *SPG21* KO HeLa cells. Scale bar = 10 µm. *n* = 6 (three independent experiments, including two clones per genotype). 10 cells were analyzed per clone in each experiment. These values are shown in light gray. Their averages are shown as follows: open circles: CTRL1; open squares: CTRL2; black triangles: KO1; black diamonds: KO2. Mean Manders’ coefficients ± SD. Two-tailed unpaired *t* test. ns, nonsignificant. **(B)** Colocalization analysis between HA-RagA and LAMP2 in CTRL and *SPG21* KO HeLa cells as described in A. **(C)** Western blotting analysis of pTFEB/TFEB ratio in CTRL and *SPG21* KO HeLa cells 48 h after transfection of GFP-TFEB with either an empty plasmid, a folliculin-His and/or a Flag-FNIP2 construct. α-Tubulin was used as a loading control. Folliculin and FNIP expressions were assessed using anti-His and anti-Flag antibodies, respectively. The graph shows the quantifications of the mean fold change in pTFEB/TFEB ratio ± SD in three independent experiments for two KO clones (*n* = 6). *n* = 12 for the CTRL cells (each of the six blots contained the two CTRL clones co-transfected with GFP-TFEB and an empty plasmid. One of them is shown in the figure). Two-tailed unpaired *t* test. ns, nonsignificant; *P < 0.05; **P < 0.01. **(D)** Confocal microscopy detection of GFP-TFEB distribution in CTRL and *SPG21* KO HeLa cells 72 h after transfection with folliculin-His (red) and/or Flag-FNIP2 (blue) constructs. Scale bar = 10 µm. The graphs show the percentage of cells with predominant nuclear localization of TFEB (left) or the TFEB nuclear/cytoplasmic signal ratio (right). Mean ± SD. *n* = 6 (three independent experiments for two CTRL and two KO clones). 10 cells were analyzed per clone in each experiment, as described in (B). Two-tailed unpaired *t* test. ns, non-significant; ****P < 0.0001. **(E)** Western blotting detection of endogenously expressed TFEB in CTRL and *SPG21* KO cells transfected with a Flag-FNIP2 construct for 48 h. One representative set of four independent experiments is shown. The black arrowhead indicates the higher MW forms of TFEB (pTFEB). The white arrowhead indicates lower MW forms of TFEB (non-phosphorylated). Source data are available for this figure: SourceData F3.

No difference in RagC (nor RagA) colocalization level with the late endosomal/lysosomal marker LAMP2 was found in *SPG21* KO HeLa cells (Fig. 3, A and B). Thus, it appears that the loss of maspardin expression does not affect RagC recruitment to lysosomes but rather its ability to present TFEB to mTOR for phosphorylation, suggesting that it may be predominantly in a GTP-bound (inactive) state in the maspardin-deficient cells. To test this hypothesis, we analyzed TFEB phosphorylation and localization after transfection of the *SPG21* KO cells with components of the folliculin:folliculin–interacting protein 2 (FLCN:FNIP2) complex, which acts as a GTPase-activating protein (GAP) for RagC (i.e., it enables RagC activation via FLCN-mediated hydrolysis of its GTP into GDP) (Tsun et al., 2013). These experiments revealed that the combined overexpression of FNIP2 and FLCN, as well as FNIP2 alone (which recruits endogenous FLCN on membranes, Fig. S2 B), rescued GFP-TFEB phosphorylation and localization in *SPG21* KO HeLa cells (Fig. 3, C and D). FNIP overexpression also increased the MW of TFEB expressed endogenously in these KO cells, consistent with a rescue of its phosphorylation (Fig. 3 E). N.B. Transfection of FLCN/FNIP constructs in controls cells are shown in Fig. S2 C (they do not significantly alter pTFEB levels in those cells).

### Defective TFEB presentation to mTOR by RagC in *SPG21* KO cells results from abnormal RAB7 recruitment to lysosomes

**Figure 4.**
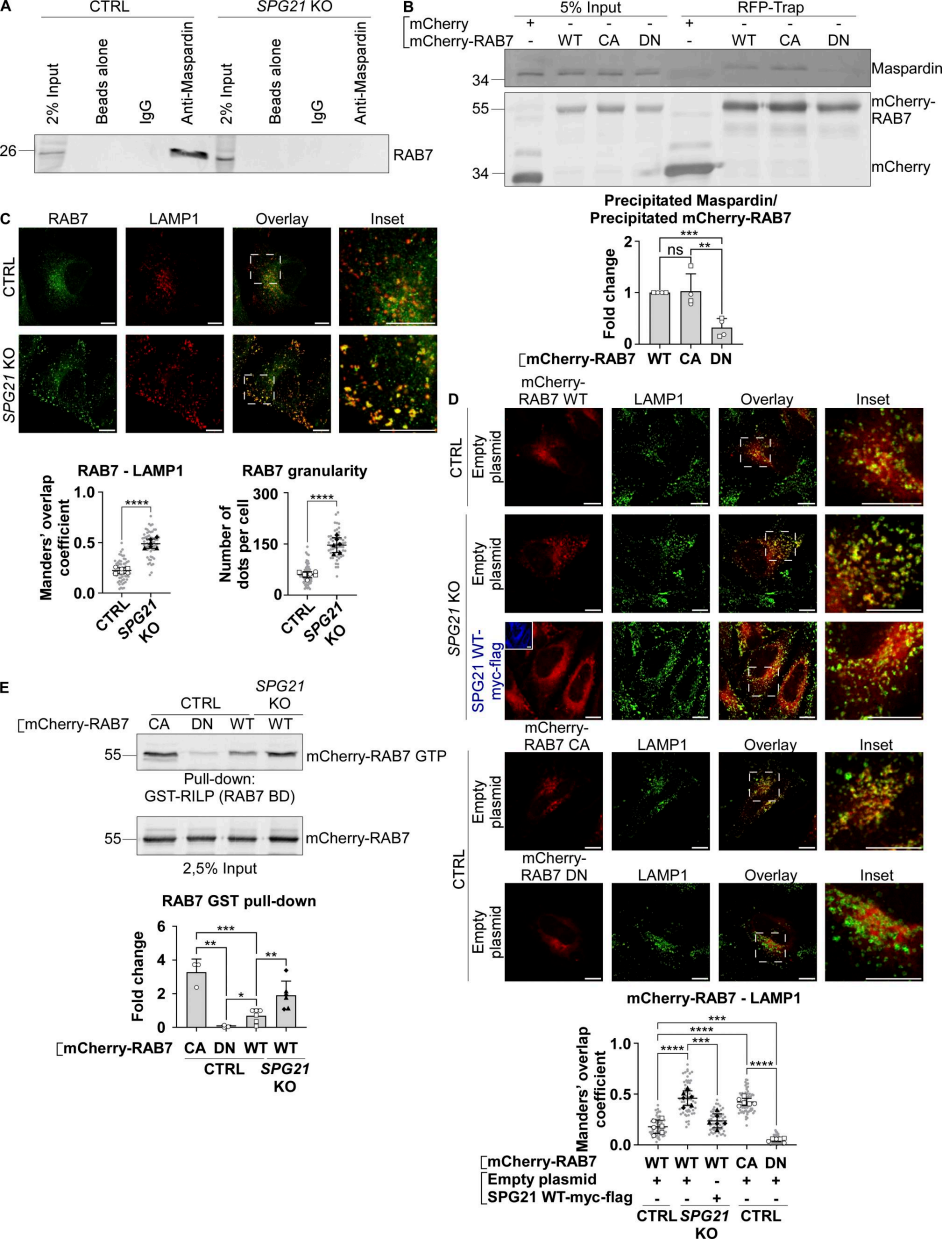
**Maspardin deficiency leads to RAB7 hyperactivation. (A)** Co-immunoprecipitation analysis of maspardin and RAB7 in CTRL and, as negative control, *SPG21* KO HeLa cells. Precipitation of maspardin using protein A-magnetic beads preincubated with an anti-maspardin antibody, followed by endogenous RAB7 detection by western blotting. Beads alone and beads incubated with non-targeting IgG were included as control conditions. **(B)** Expression of WT, CA, or DN forms of mCherry-RAB7 or mCherry alone in control cells followed by their precipitation using RFP-trap beads. The presence of endogenous maspardin among the coprecipitated proteins was detected by western blotting. *n* = 4 (two independent experiments, including two CTRL clones [open circles: CTRL1; open squares: CTRL2]). The graph shows mean fold changes ± SD of maspardin signals normalized to the amount of precipitated bait proteins. Two-tailed unpaired *t* test. ns, nonsignificant; **P < 0.01; ***P < 0.001. **(C)** Colocalization analysis between endogenously expressed RAB7 (green) and LAMP1 (red) in CTRL and *SPG21* KO HeLa cells. Scale bar = 10 µm. The graphs show the quantifications of RAB7 granularity (right graph) and RAB7-LAMP1 colocalization (left graph, Manders’ coefficients). *n* = 6 (three independent experiments, including two CTRL and two KO clones). 10 cells were analyzed per clone in each experiment (shown in light gray). The averaged values are shown as follows: open circles: CTRL1; open squares: CTRL2; black triangles: KO1; black diamonds: KO2. Mean ± SD. Two-tailed unpaired *t* test. ****P < 0.0001. **(D)** Colocalization analysis between WT, CA, and DN mCherry-RAB7 proteins and LAMP1 (green) in CTRL and *SPG21* KO HeLa cells (48 h after transfection). SPG21 WT-myc-flag (blue) was re-expressed in KO cells as a rescue control. Scale bar = 10 µm. The graph shows RAB7-LAMP1 colocalization (Mean Manders’ coefficients ± SD). *n* = 6 (three independent experiments, including two CTRL and two KO clones). 10 cells were analyzed per clone in each experiment, as described in B. Two-tailed unpaired *t* test. ***P < 0.001; ****P < 0.0001. **(E)** GST pull-down analysis of mCherry-RAB7 in CTRL and *SPG21* KO HeLa cells using GST-RILP (RAB7-binding domain [BD]) as bait. CA and DN RAB7 constructs were used as positive and negative controls, respectively. Western blotting was conducted 24 h after transfection using an anti-tdTomato tag antibody. The graph shows the ratios of bound RAB7 to total RAB7 signal calculated in three independent experiments with two CTRL and two KO clones transfected with WT RAB7 in each experiment (*n* = 6) and one CTRL clone transfected with CA and DN RAB7 (*n* = 3). Mean ± SD. Two-tailed unpaired *t* test. ns, nonsignificant; *P < 0.05; **P < 0.01; ***P < 0.001. RFP, red fluorescent protein. Source data are available for this figure: SourceData F4.

As mentioned in the introduction, the small GTPase RAB7 has been identified as a maspardin-binding partner in HEK293T cells (McCray et al., 2010). We validated this finding by co-immunoprecipitation of endogenously expressed maspardin and RAB7 in HeLa cells, using the *SPG21* KO cells as a negative control (Fig. 4 A). Additionally, when using CA or dominant-negative (DN) forms of RAB7 (tagged with mCherry) as bait, we found that maspardin preferentially binds to the GTP-bound (i.e., active) form of this GTPase (Fig. 4 B, P < 0.01). These data suggest that maspardin might act as a RAB7 effector on late endosomes or lysosomes.

**Figure S3.**
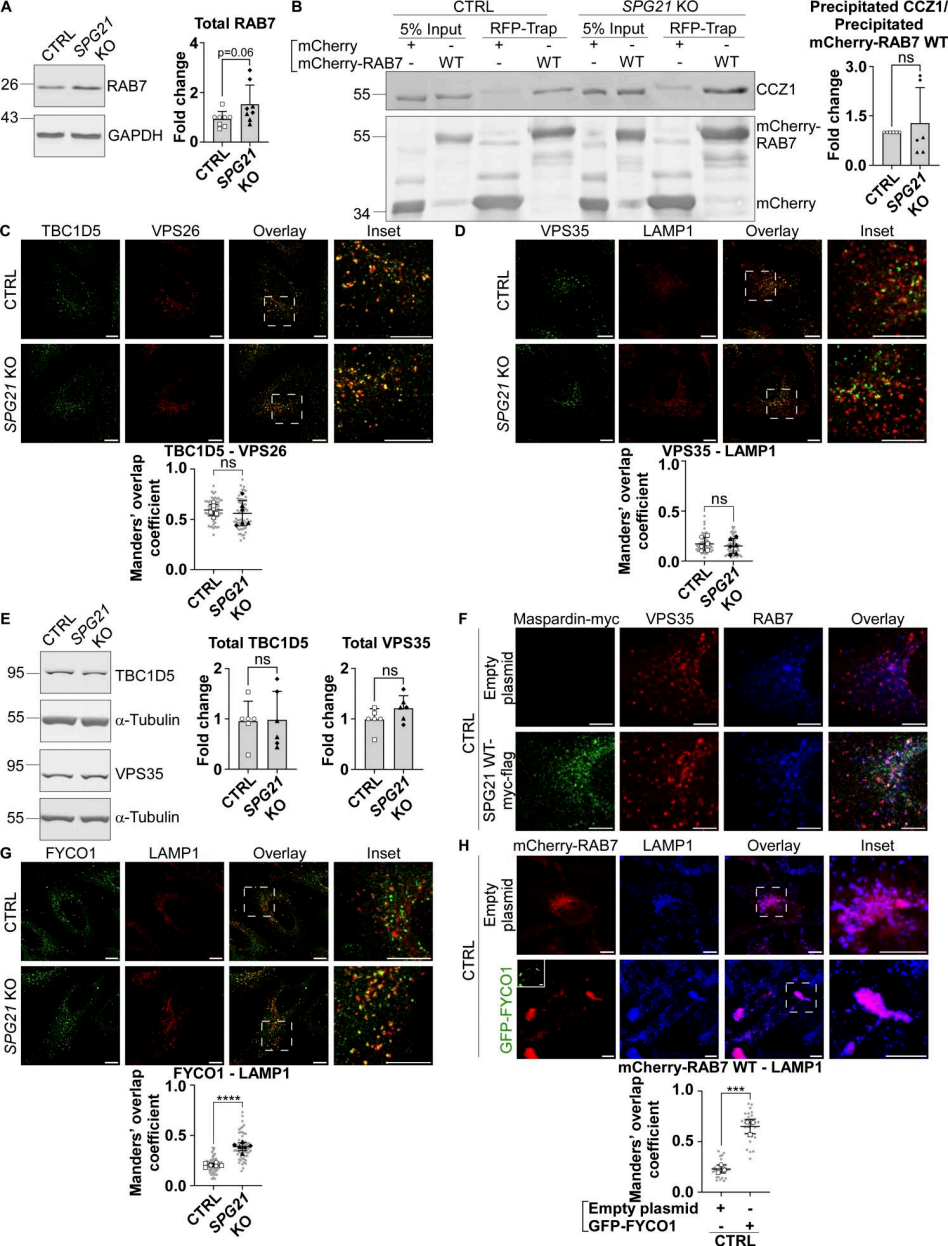
**Basal expression levels of RAB7, TBC1D5, and VPS35 in CTRL and *SPG21* KO cells; coprecipitation assay of CCZ1 with RAB7; colocalization analyses between TBC1D5 and VPS26, VPS35 and LAMP1, maspardin-myc, VPS35 and RAB7, and FYCO1 and LAMP1; and assessment of the presence of RAB7 on lysosomes when FYCO1 is overexpressed. (A and E)** Western blotting detection of endogenously expressed RAB7, TBC1D5, and VPS35 in CTRL and *SPG21* KO cells. α-Tubulin or GAPDH was used as a loading control. Of note, RAB7 and GAPDH signals are also presented in Fig. 5 B. The graphs show the mean fold change ± SD. For RAB7 expression experiment, *n* = 8 (four independent experiments, including two CTRL [open circles: CTRL1; open squares: CTRL2] and two KO clones [black triangles: KO1; black diamonds: KO2]); for TBC1D5 and VPS35 expression experiments, *n* = 6 (three independent experiments, including two CTRL and two KO clones). Two-tailed unpaired *t* test. ns, nonsignificant; P = 0.06. **(B)** Expression of WT mCherry RAB7 or mCherry alone in CTRL and *SPG21* KO cells followed by their precipitation using RFP-trap beads. The presence of endogenous CCZ1 proteins among the coprecipitated proteins was detected by western blotting. *n* = 6 (three independent experiments, including two CTRL and two KO clone). The graph shows mean fold changes ± SD of precipitated CCZ1 signals normalized to the amount of precipitated bait proteins. Two-tailed unpaired *t* test. ns, nonsignificant. **(C, D, F and G)** Colocalization analysis between endogenously expressed TBC1D5 (green) and VPS26 (red), between VPS35 (green) and LAMP1 (red), between maspardin-myc (green), VPS35 (red) and RAB7 (blue), and between FYCO1 (green) and LAMP1 (red) in CTRL and KO cells (micrographies of individual channels corresponding to the triple labellings shown in Fig. 7 F). Scale bar = 10 µm. The graph shows the Manders’ coefficient. *n* = 6 (three independent experiments, including two CTRL and two KO clones) except for the triple labelling, where *n* = 5 independent experiments. 10 cells were quantified per clone in each experiment. These values are shown in light gray. Their averages are shown as follows: open circles: CTRL1; open squares: CTRL2; black triangles: KO1; black diamonds: KO2. Mean ± SD. Two-tailed unpaired *t* test. ns, non-significant; ****P < 0.0001. Scale bar = 10 µm except for the triple labelling (scale bar = 5 µm). **(H)** Analysis of the colocalization level (48 h after transfection) between mCherry-RAB7 WT and LAMP1 (blue) after transfection of GFP-FYCO1 or of an empty plasmid. Scale bar = 10 µm. The graph shows the quantifications of *n* = 3 independent experiments. 10 cells were analyzed per condition in each experiment, as described above. Mean ± SD. Two-tailed unpaired *t* test. ****P < 0.0001. RFP, red fluorescent protein. Source data are available for this figure: SourceData FS3.

Based on these observations, and since it has been documented in the literature that an increased RAB7 recruitment to late endosomes/lysosomes can disrupt Rag function (Kvainickas et al., 2019), we decided to analyze the intracellular distribution of RAB7 by confocal microscopy in control and *SPG21* KO cells. Interestingly, the distribution pattern of endogenously expressed RAB7 was found to be more punctate in the KO cells compared with control cells (Fig. 4 C). This was reflected by an increase in the RAB7 granularity index (Fig. 4 C, P < 0.0001) and a concomitant increased colocalization of RAB7 with LAMP1-positive vesicles (Fig. 4 C, P < 0.0001). Total intracellular levels of RAB7 were found slightly elevated (1.5-fold) as a result (Fig. S3 A, P = 0.06).

These results raise the hypothesis that loss of maspardin may disrupt the activation and/or inactivation cycle of RAB7 in a way that favors its GTP-bound state (i.e., its active membrane-bound form). Indeed, the punctate pattern and increased presence of endogenous RAB7 on LAMP1 vesicles in *SPG21* KO cells, which is reproduced using transfected mCherry-RAB7 (Fig. 4 D), are reminiscent of the distribution of the CA form of mCherry-RAB7 (GTP-bound, included as a control in Fig. 4 D). In contrast, the DN form of mCherry-RAB7 (GDP-bound) appeared as a diffuse signal in the cytoplasm (Fig. 4 D). Of note, the punctate pattern of mCherry-RAB7 dissipated after re-expression of maspardin (Fig. 4 D). In addition, using the RAB7 binding domain of murine RILP, a RAB7 effector, as bait in a GST pull-down assay (Romero Rosales et al. 2009), we demonstrated that *SPG21* KO cells contain more GTP-bound RAB7 molecules than control cells, since RILP preferentially binds to this form (Fig. 4 E, P < 0.01).

**Figure 5.**
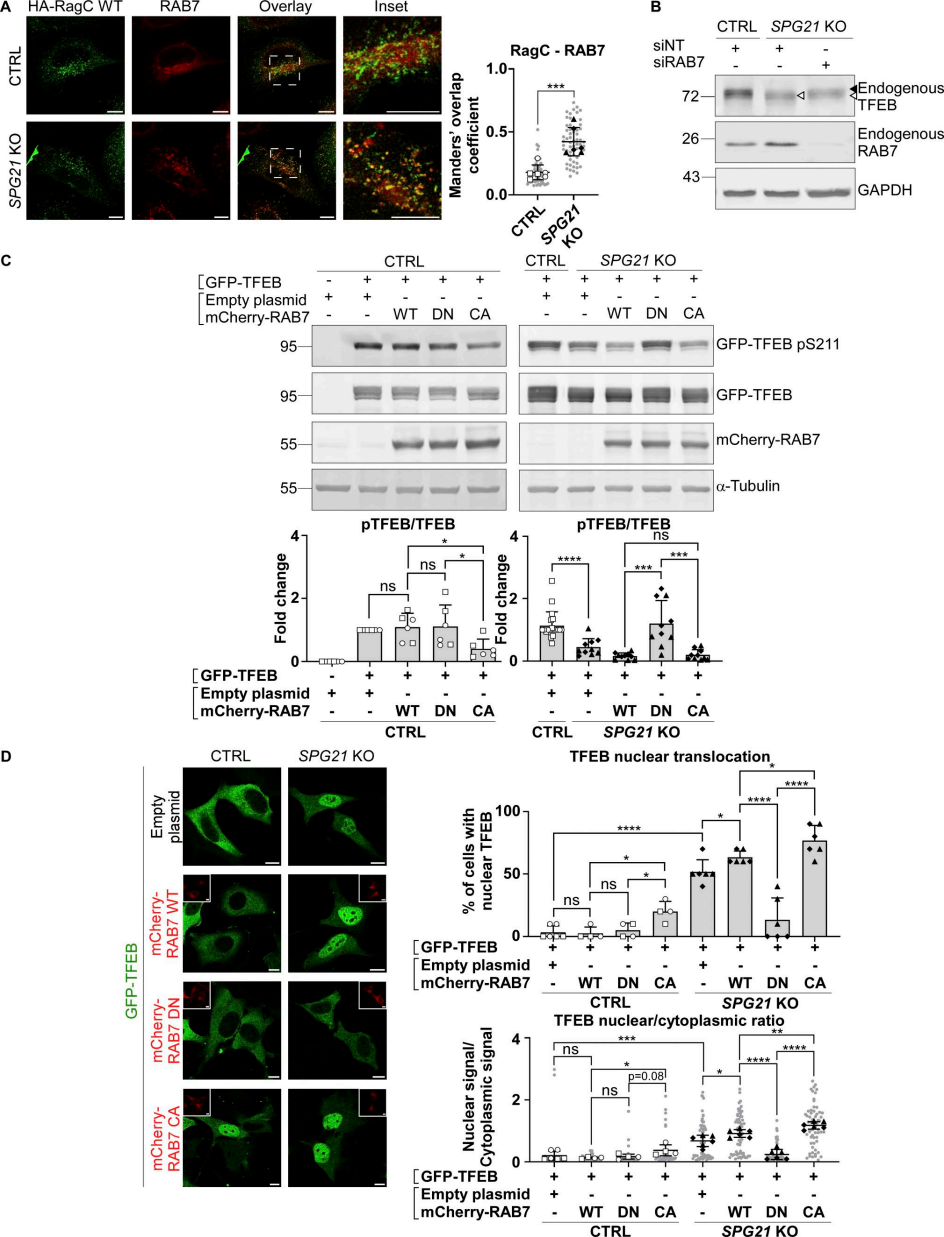
**Increased RAB7 GTP/GDP ratio in *SPG21* KO HeLa cells impairs TFEB phosphorylation by mTOR. (A)** Colocalization analysis between endogenously expressed RAB7 and transfected HA-RagC WT in CTRL and *SPG21* KO HeLa cells. Scale bar = 10 µm. The graphs show Mean Manders’ coefficients ± SD. *n* = 6 (three independent experiments, including two CTRL and two KO clones). 10 cells were analyzed per clone in each experiment (light gray). The averaged values are shown as follows: open circles: CTRL1; open squares: CTRL2; black triangles: KO1; black diamonds: KO2. Two-tailed unpaired *t* test. ***P < 0.001. **(B)** Western blotting detection of endogenously expressed TFEB in CTRL and *SPG21* KO cells transfected for 48 h with siRNAs targeting *RAB7*. Non-targeting siRNAs (siNT) were used as controls. One representative set of *n* = 4 independent experiments is shown. The black arrowhead indicates the higher MW forms of TFEB (pTFEB). The white arrowhead indicates lower MW forms of TFEB (non-phosphorylated). **(C)** Western blotting analysis of pTFEB/TFEB ratio 48 h after transfection of *SPG21* KO HeLa cells (right panel) or control cells (left panel) with GFP-TFEB and either WT, DN, or CA mCherry-RAB7. α-Tubulin and RAB7 chimeric proteins were detected as controls. Right panel: *n* = 6 (three independent experiments, including two CTRL clones); left panel: *n* = 10 for the KO cells (five independent experiments, including two different KO clones) and *n* = 20 for the CTRL cells (each of the 10 blots contained the two CTRL clones co-transfected with GFP-TFEB and an empty plasmid. One of them is shown in the figure). Mean fold change ± SD. Two-tailed unpaired *t* test. ns, nonsignificant; *P < 0.05; ***P < 0.001; ****P < 0.0001. **(D)** Investigation of GFP-TFEB localization by confocal microscopy in CTRL and *SPG21* KO HeLa cells after transfection with the same constructs. Scale bar = 10 µm. The graphs show the quantification of the percentage of cells with predominant nuclear localization for TFEB (upper graph) or the TFEB nuclear/cytoplasmic signal ratio (lower graph). Mean ± SD. For the control conditions transfected with RAB7 constructs, *n* = 4 (two independent experiments including two different CTRL clones). For the KO conditions, *n* = 6 (three independent experiments, including two different KO clones). 10 cells were analyzed per clone, as described in A. Two-tailed unpaired *t* test. ns, nonsignificant; *P < 0.05; **P < 0.01; ***P < 0.01; ****P < 0.0001. Source data are available for this figure: SourceData F5.

**Figure 6.**
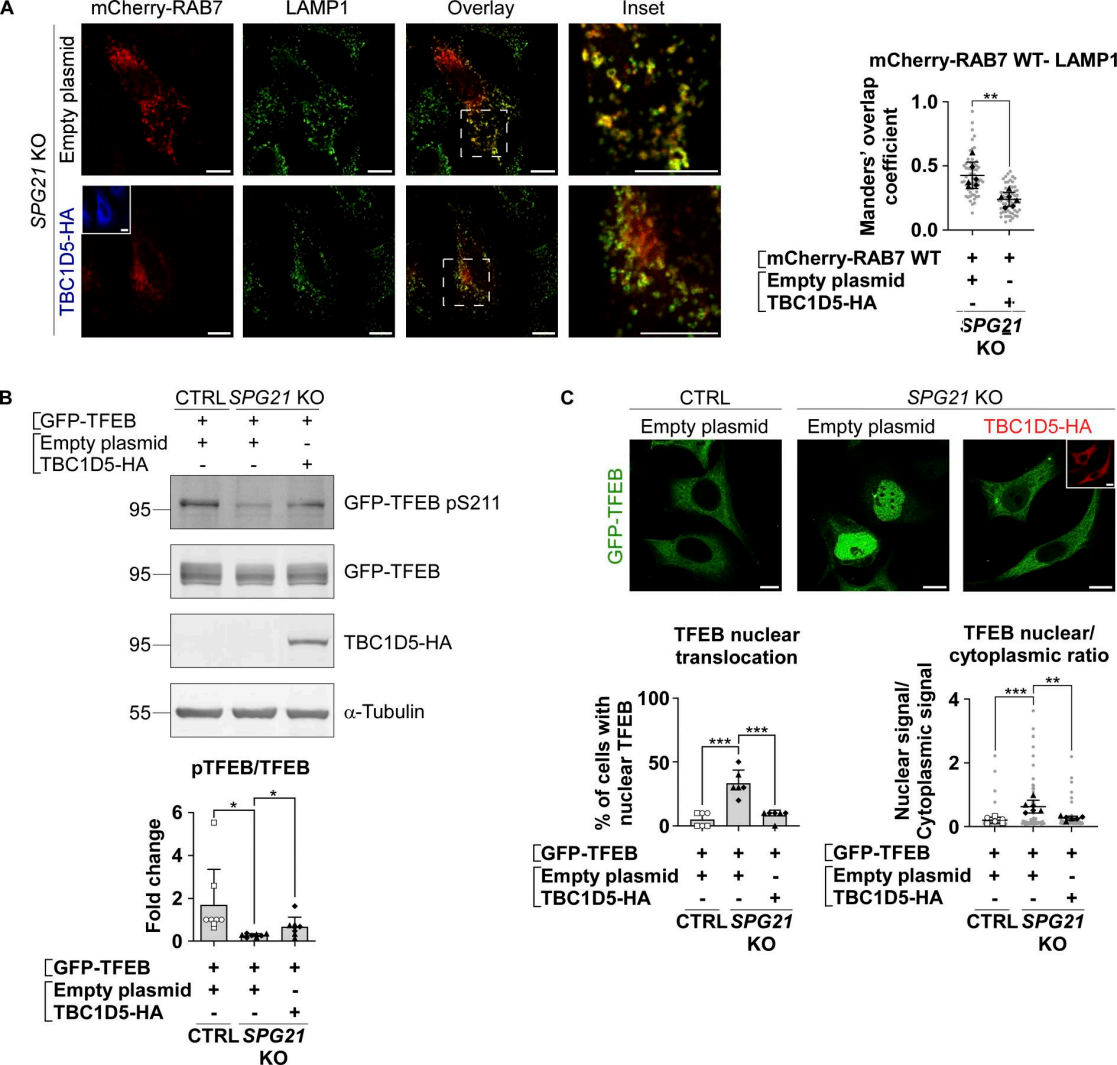
**Removal of RAB7 from lysosomal membranes rescues TFEB phosphorylation level and localization. (A)** Colocalization analysis between mCherry-RAB7 WT and LAMP1 (green) in *SPG21* KO HeLa cells 48 h after transfection with an empty plasmid or TBC1D5-HA (blue). Scale bar = 10 µm. The graphs show the quantifications (Manders’ coefficient) of three independent experiments with two different KO clones (*n* = 6). 10 cells were analyzed per clone in each experiment (shown in light gray). The average values for each clone are shown as follows: black triangles: KO1; black diamonds: KO2. Mean ± SD. Two-tailed unpaired *t* test. **P < 0.01. **(B)** Western blotting detection of the pTFEB/TFEB ratio in CTRL and *SPG21* KO HeLa cells 48 h after the co-transfection of GFP-TFEB with an empty plasmid or a TBC1D5-HA construct (detected with an anti-HA antibody). The graph shows mean fold changes in pTFEB/TFEB ratio ± SD. *n* = 8 (four independent experiments with two CTRL and two KO clones). Two-tailed unpaired *t* test. *P < 0.05. **(C)** Analysis of GFP-TFEB localization by confocal microscopy in CTRL and *SPG21* KO HeLa cells 72 h after co-transfection with an empty plasmid or a TBC1D5-HA (red) construct. Scale bar = 10 µm. The graphs show the percentage of cells with predominant nuclear TFEB (left graph) or the TFEB nuclear/cytoplasmic signal ratio (right graph). *n* = 6 (three independent experiments including two CTRL and two KO clones). 10 cells were analyzed per clone in each experiment as described in A. Open circles: CTRL1; open squares: CTRL2; black triangles: KO1; black diamonds: KO2. Mean ± SD. Two-tailed unpaired *t* test. **P < 0.01; ***P < 0.001. Source data are available for this figure: SourceData F6.

Next, we investigated whether this increased recruitment of RAB7 to late endosomes/lysosomes in maspardin-deficient cells could be responsible for the decreased RagC-mediated presentation of TFEB to mTOR, resulting in its decreased phosphorylation, as described in the previous section. To this end, we first analyzed the distribution of endogenous RAB7 relative to RagC-positive lysosomes (identified after transfection of WT RagC). In accordance with the increase in RAB7-LAMP1 colocalization shown above, we found that there was more RAB7 on RagC-positive vesicles (Fig. 5 A, P < 0.001). Next, we silenced *RAB7* expression with siRNAs in *SPG21* KO cells. Interestingly, this resulted in a slight increase in the MW of endogenous TFEB, consistent with a rescue of its phosphorylation (Fig. 5 B). We then analyzed the phosphorylation level and localization of GFP-TFEB in *SPG21* KO cells after co-transfection with the WT, DN (cytosolic), or CA (membrane-bound) forms of RAB7. This experiment revealed that the DN form of RAB7 can restore GFP-TFEB phosphorylation (Fig. 5 C, right panel, P < 0.001) and cytoplasmic distribution in the KO cells (Fig. 5 D, P < 0.0001). In contrast, overexpression of the CA form of RAB7 decreased phosphorylation and promoted nuclear translocation of GFP-TFEB in control cells (Fig. 5, C and D, P < 0.05). Furthermore, overexpression of TBC1D5 in the *SPG21* KO cells, a GAP that promotes GTP hydrolysis in RAB7 and thus triggers its detachment from membranes (Seaman et al., 2009), decreased RAB7 colocalization with LAMP1 (Fig. 6 A, P < 0.01), raised GFP-TFEB phosphorylation level (Fig. 6 B, P < 0.05), and rescued its localization (Fig. 6 C, P < 0.001 when assessing the percentage of cells with nuclear TFEB, and P < 0.01 for TFEB nuclear/cytoplasmic ratio). See Fig. S2 D for transfection of TBC1D5 in control cells.

These results support that maspardin is a RAB7 effector and that its loss induces RAB7 hyperactivation/retention in a GTP-bound form, which subsequently leads to defective TFEB presentation to mTOR by RagC and decreased phosphorylation.

### Maspardin controls the presence of RAB7 on retromer-positive vesicles

The high levels of GTP-bound RAB7 in maspardin-deficient cells could hypothetically result from increased GTP loading by the RAB7 GEF MON1-CCZ1, but co-immunoprecipitation experiments revealed similar levels of interaction between RAB7 and this GEF in *SPG21* KO cells and control cells (Fig. S3 B). Alternatively, GTP-hydrolysis induced by a GAP, such as TBC1D5, could be compromised.

**Figure 7.**
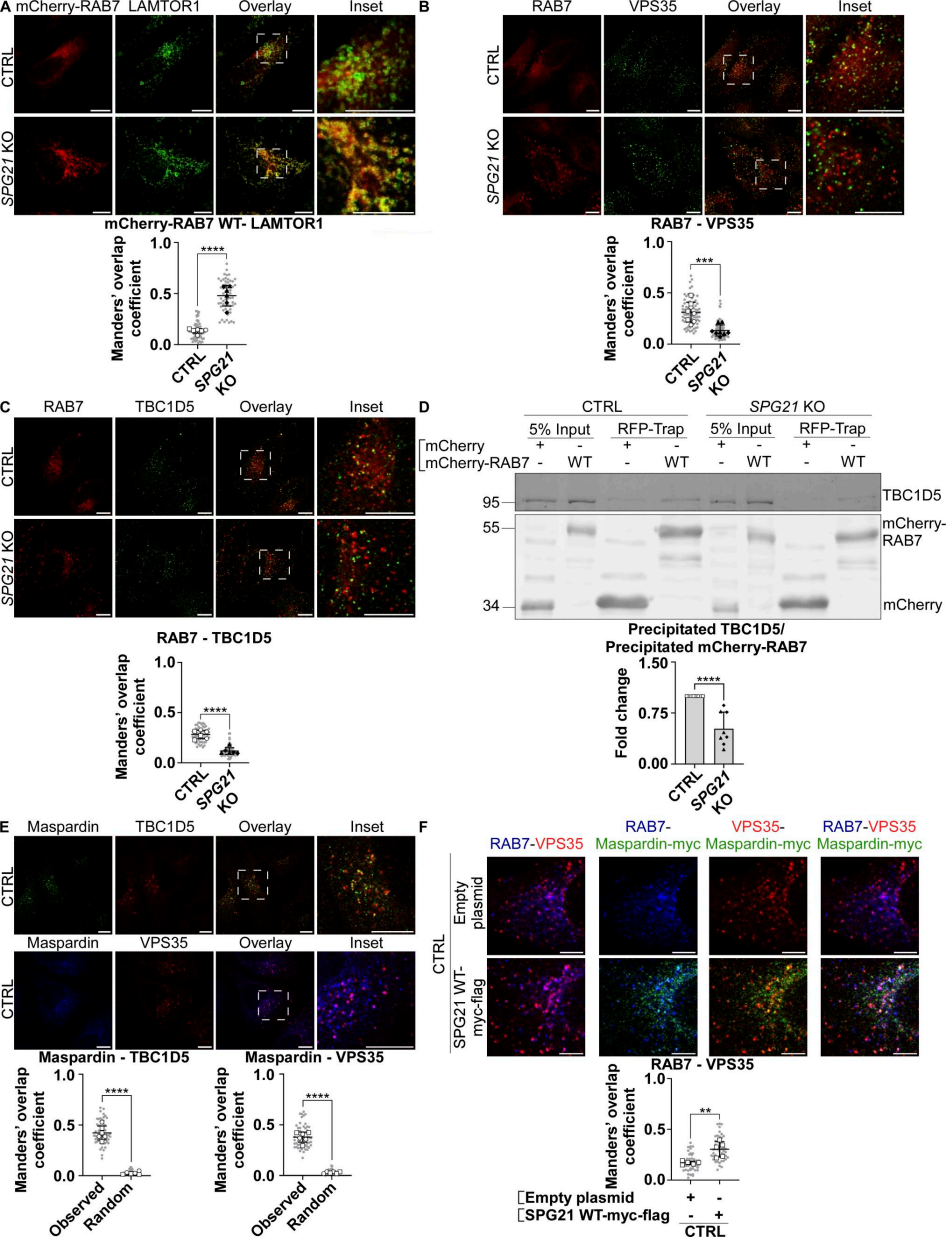
**Maspardin deficiency causes delocalization of RAB7 molecules from late endosomes to lysosomes. (A–C)** Colocalization analyses between mCherry-RAB7 WT and endogenous LAMTOR1 (green) (A), endogenous RAB7 (red) and VPS35 (green) (B), and endogenous RAB7 (red) and TBC1D5 (green) (C) in CTRL and *SPG21* KO HeLa cells. Scale bar = 10 µm. The graphs show mean Manders’ coefficients ± SD. For RAB7-LAMTOR1 and RAB7-TBC1D5 colocalization analyses, *n* = 6 (three independent experiments with two CTRL and two KO clones); for RAB7-VPS35 analyses, *n* = 8 (four independent experiments with two CTRL and two KO clones). 10 cells were analyzed per clone in each experiment (shown in light gray). Means of these values for each clone are indicated as follows: open circles: CTRL1; open squares: CTRL2; black triangles: KO1; black diamonds: KO2. Two-tailed unpaired *t* test. ***P < 0.001; ****P < 0.0001. **(D)** Expression of WT mCherry RAB7 or mCherry alone in CTRL and *SPG21* KO cells followed by their precipitation using RFP-trap beads. The presence of endogenous TBC1D5 proteins among the coprecipitated proteins was assessed by western blotting. *n* = 8 (four independent experiments, each including 2 CTRL and 2 KO clones). The graph shows mean fold changes ± SD of precipitated TBC1D5 signals normalized to the amount of precipitated bait proteins. Two-tailed unpaired *t* test. ****P < 0.0001. **(E)** Colocalization analysis between endogenous maspardin (green) and TBC1D5 (red) or between endogenous maspardin (blue) and VPS35 (red) in CTRL cells. The random overlap between signals was assessed as a negative control by rotating one of the images by 90°. Scale bar = 10 µm. Mean Manders’ coefficient ± SD. *n* = 6 (three independent experiments including two CTRL clones). 10 cells analyzed per biological replicate, as described above. Two-tailed unpaired *t* test. ****P < 0.0001. **(F)** Analysis of endogenous RAB7 distribution (blue) relative to endogenous VPS35 (red) 48 h after transfection of SPG21-myc-flag (green) or a mock construct in control cells (micrographies of individual channels are provided in Fig. S3 F). Scale bar = 5 µm. Mean Manders’ coefficient ± SD. *n* = 5 independent experiments. 10 cells analyzed per control clone (shown in light gray). The means for each CTRL clone are indicated with different white symbols. Two-tailed unpaired *t* test. **P < 0.01. RFP, red fluorescent protein. Source data are available for this figure: SourceData F7.

TBC1D5 is reported to be primarily active on late endosomal compartments. It is known to associate with RAB7 and retromer complex (mainly VPS29 and VPS35), which increases its activity toward RAB7 (Jimenez-Orgaz et al., 2018; Kvainickas et al., 2019) We documented above that there is an increased colocalization of RAB7 (endogenous or transfected) with the late endosomal/lysosomal marker LAMP1 and with RagC-positive lysosomes after *SPG21* KO (see Figs. 4, C and D; and 5 A). An increase of colocalization level was also detected between mCherry-RAB7 and LAMTOR1 (a component of the Ragulator complex that supports mTORC1 assembly) (Fig. 7 A, P < 0.0001), further supporting that maspardin deficiency increases the presence of RAB7 on lysosomes. Most interestingly, we detected a concomitant decrease in the presence of RAB7 on TBC1D5- and VPS35-positive organelles in the KO cells (Fig. 7, B and C, P < 0.001). This translated into reduced interaction between RAB7 and TBC1D5 in co-immunoprecipitation experiments (Fig. 7 D, P < 0.0001). Notably, there was no change in the level of colocalization between TBC1D5 and the retromer complex, or between VPS35 and LAMP1 in the KO cells, and no change of their expression levels either (see Fig. S3, C–E). Therefore, we infer that maspardin is a RAB7 effector required to maintain this GTPase on late endosomes, where the TBC1D5 GAP is reported to be active. This limits contact of RAB7 with the mTORC1 signaling platform. This inference is further supported by the finding that maspardin is present on TBC1D5- and VPS35-positive vesicles (Fig. 7 E) and by the observation that RAB7 and maspardin colocalize on VPS35-positive vesicles in control cells (Fig. 7 F, white dots in this triple labelling). Note that separate micrographies of the individual channels of this triple labelling are provided in Fig. S3 F. Furthermore, overexpressing maspardin in these control cells increases the colocalization level between RAB7 and VPS35 (Fig. 7 F, P < 0.01).

### Abnormal recruitment of RAB7 on lysosomes of maspardin-deficient cells slightly alters the expression of some CLEAR network genes and causes FYCO1-dependent redistribution of lysosomes to the cell periphery

**Figure 8.**
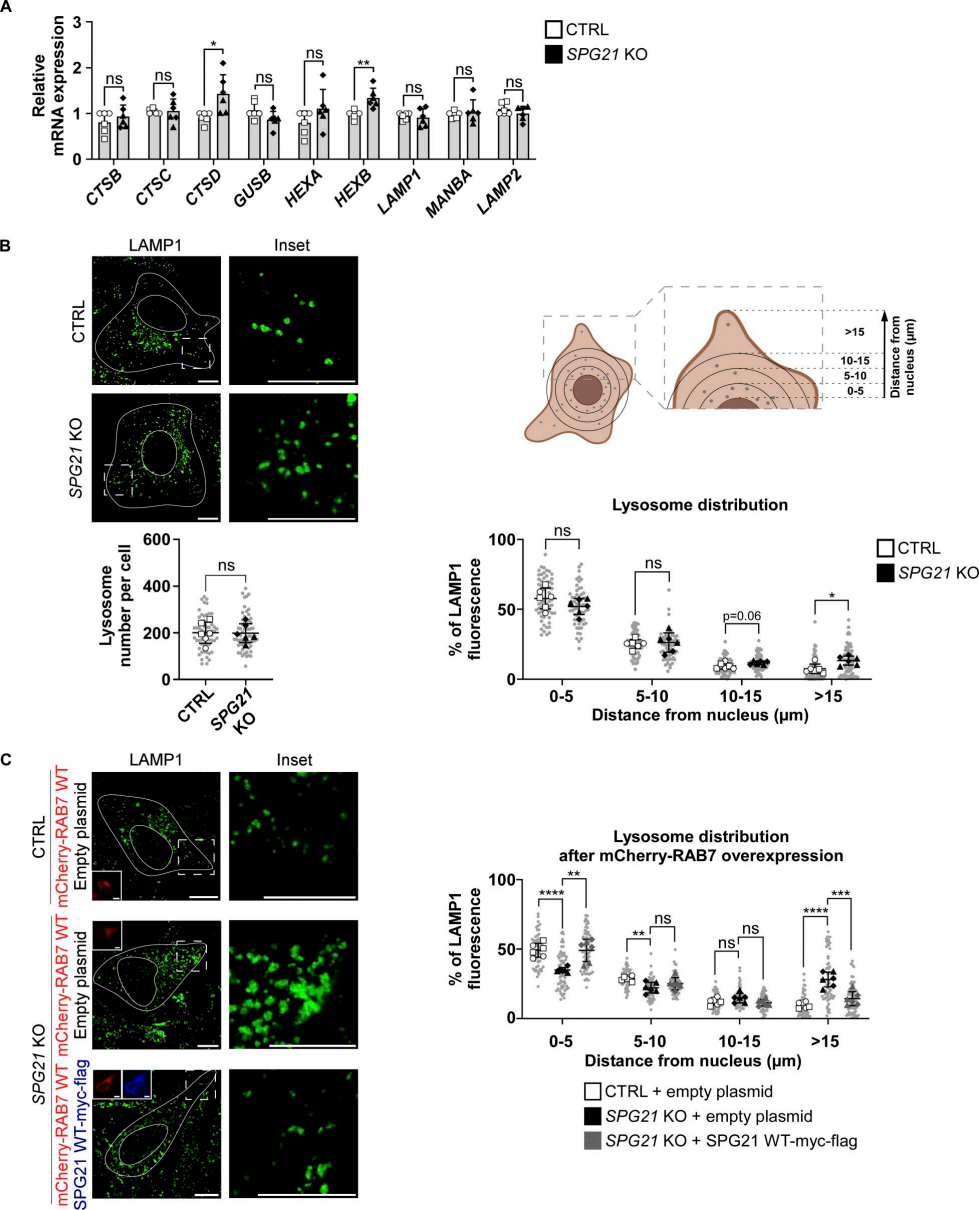
**The KO of *SPG21* slightly increases the expression of some CLEAR genes and promotes the anterograde transport of lysosomes in HeLa cells. (A)** RT-qPCR analysis of the expression of genes belonging to the CLEAR network in CTRL and *SPG21* KO cells: *CTSB*, *CTSC*, *CTSD*, *GUSB*, *HEXA*, *HEXB*, *LAMP1*, and *MANBA. LAMP2* was included as a control. The graph shows the relative mRNA expression of these genes normalized to *GAPDH* expression. *n* = 6 (three independent experiments with two CTRL [open circles: CTRL1; open squares: CTRL2] and two KO clones [black triangles: KO1; black diamonds: KO2]). Mean fold changes ± SD. Two-tailed unpaired *t* test. ns, nonsignificant; *P < 0.05; **P < 0.01. **(B)** Investigation of lysosome number and distribution in CTRL and *SPG21* KO HeLa cells. Lysosomes were detected with an anti-LAMP1 antibody (green). Scale bar = 10 µm. Graphs represent the number of lysosomes per cell (left graph) and lysosome distribution (right graph). Lysosome distribution was determined by drawing four circles around the nucleus at 5-µm intervals and by calculating the relative LAMP1 fluorescence intensity in each interval as shown in the schematic representation. *n* = 6 (three independent experiments with two CTRL and two KO clones). 10 cells were analyzed per clone in each experiment. These values are shown in light gray. Their averages are shown as follows: open circles: CTRL1; open squares: CTRL2; black triangles: KO1; black diamonds: KO2. Mean ± SD. Two-tailed unpaired *t* test. ns, nonsignificant; *P < 0.05. **(C)** Analysis of lysosome distribution in CTRL and *SPG21* KO cells 48 h after co-transfection with an mCherry-RAB7 construct and with an empty plasmid or an SPG21 WT-myc-flag construct (blue) (note that this is the same experiment presented in Figure 4D, but the data is now analyzed for LAMP distribution). Lysosomes were detected with an anti-LAMP1 antibody (green). Scale bar = 10 µm. The graph shows the quantifications of lysosome distribution in cells overexpressing mCherry-RAB7 as explained in B. *n* = 6 (three independent experiments, including two CTRL and two KO clones). 10 cells were analyzed per clone, as described in B. Mean ± SD. Two-tailed unpaired *t* test. ns, nonsignificant; **P < 0.01; ***P < 0.001; ****P < 0.0001.

Since TFEB is known to regulate the expression of genes belonging to the CLEAR network, thereby inducing lysosomal biogenesis and autophagy-mediated clearance, we analyzed the expression of several of these genes by qPCR (*CTSB [cathepsin B]*, *CTSC [cathepsin C]*, *CTSD* [cathepsin D], *GUSB [β-glucuronidase]*, *HEXA* and *HEXB* [β-hexosaminidase subunits], *LAMP1*, and *MANBA [β-mannosidase]*) (Fig. 8 A). Only those encoding the lysosomal protease cathepsin D and the β-subunit of lysosomal β-hexosaminidase showed a slight, statistically relevant increase of ∼1.5-fold in KO cells compared with control cells. Of note, *LAMP2* was analyzed as a control since it is not included in the list of CLEAR network genes identified by Ballabio’s group (Sardiello et al., 2009). These results suggest that the KO of *SPG21* has only moderate consequences, if any, on lysosomal biogenesis, at least not in the long term. Indeed, the number of LAMP1-positive vesicles was found to be similar in control and KO cells (Fig. 8 B).

Most interestingly, however, an analysis of the distribution of lysosomes within 5-µm circular increments drawn from the nucleus, as shown in the schematic in Fig. 8 B, revealed that lysosomes tended to localize more peripherally in maspardin-deficient cells (Fig. 8 B, P = 0.06 in the 10–15 µm area and P < 0.05 in the region >15 µm away from the nucleus). This difference in distribution was exacerbated after transfection of the WT-RAB7 construct and was rescued by the overexpression of SPG21-myc-flag (Fig. 8 C, P < 0.001 in the region >15 µm from the nucleus).

**Figure 9.**
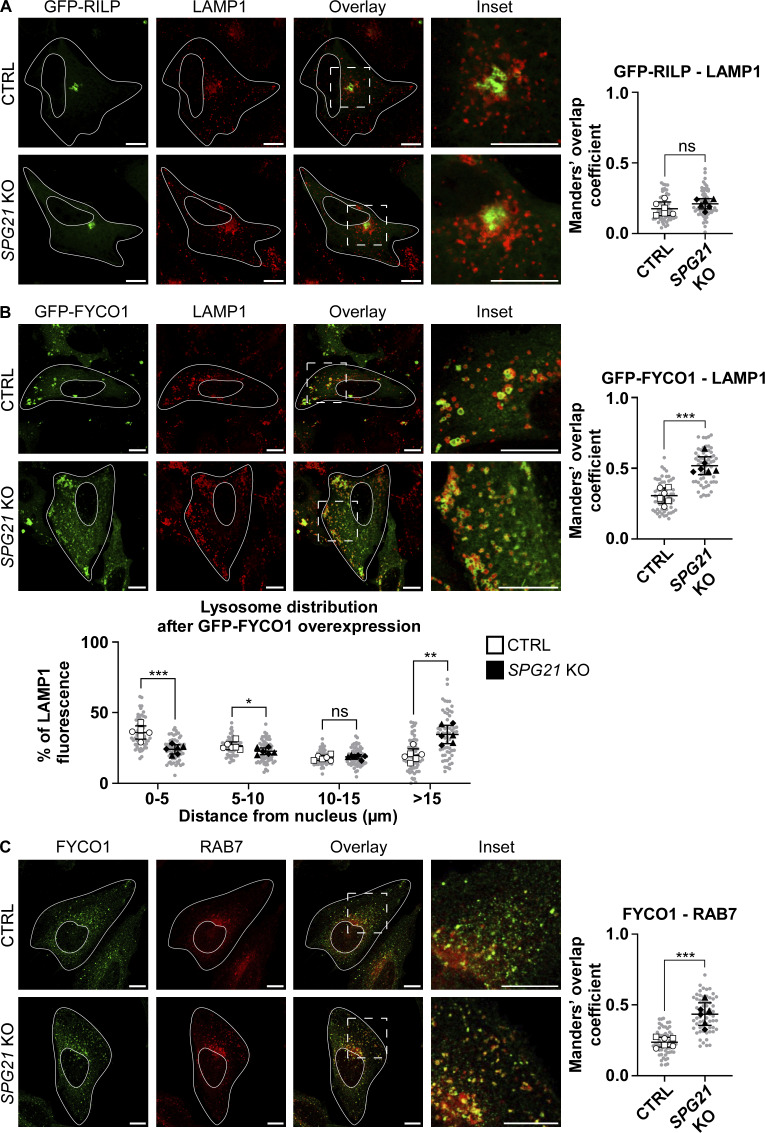
**Maspardin deficiency increases FYCO1 recruitment to lysosomes, resulting in their delocalization to the cell periphery. (A)** Analysis of lysosome distribution and colocalization between GFP-RILP and LAMP1 (red) 48 h after GFP-RILP overexpression in CTRL and *SPG21* KO HeLa cells. Scale bar = 10 µm. The graph shows RILP-LAMP1 colocalization levels (Mean Manders’ coefficient ± SD). *n* = 6 (three independent experiments including two CTRL and two KO clones). 10 cells were analyzed per clone in each experiment. These values are shown in light gray. Their averages are shown as follows: open circles: CTRL1; open squares: CTRL2; black triangles: KO1; black diamonds: KO2. Two-tailed unpaired *t* test. ns, nonsignificant. **(B)** Analysis of lysosome distribution and colocalization (Manders’ coefficients) between GFP-FYCO1 and LAMP1 (red) in CTRL and *SPG21* KO HeLa cells 48 h after transfection with GFP-FYCO1. Scale bar = 10 µm. *n* = 6 (three independent experiments with two CTRL and two KO clones). 10 cells were analyzed per clone in each experiment, as described in A. Mean ± SD. Two-tailed unpaired *t* test. ns, nonsignificant; *P < 0.05; **P < 0.01; ***P < 0.001. **(C)** Analysis of colocalization levels between endogenous FYCO1 (green) and RAB7 (red) in CTRL and *SPG21* KO HeLa cells. Scale bar = 10 µm. Mean Manders’ coefficient ± SD. *n* = 6 (three independent experiments, including two CTRL and two KO clones). 10 cells were analyzed per clone in each replicate, as described in A. Two-tailed unpaired *t* test; ***P < 0.001.

Since RAB7 is hyperactivated in the KO cells, and considering that some RAB7 effectors bind to motor machineries that mediate lysosomes transport along microtubules (Jordens et al., 2001; Pankiv et al., 2010; Wang et al., 2011), we wondered whether the recruitment to lysosomes of the effector FYCO1, which is involved in anterograde transport of lysosomes (Pankiv et al., 2010), might be increased in *SPG21* KO cells. Alternatively, the membrane presence of RILP, a RAB7 effector involved in retrograde motility, might be reduced in the absence of maspardin expression. Transfection of the cells with a GFP-tagged RILP construct resulted in a similar clustering of lysosomes in the perinuclear region of both control and KO cells (Fig. 9 A). This suggests that RILP can be recruited to lysosomes regardless of whether maspardin is expressed or not. Indeed, we found no decrease in colocalization level between RILP and LAMP1 in maspardin-deficient cells (Fig. 9 A, ns).

Most interestingly, expression of GFP-FYCO1, generously provided by Prof. Terje Johansen (The Arctic University of Norway, Tromsø, Norway) (Pankiv et al., 2010), induced peripheral delocalization of lysosomes in both cell types, but to a greater extent in *SPG21* KO cells compared with control cells (Fig. 9 B, P < 0.001 in the 0–5 µm area and P < 0.01 in the region >15 µm away from nucleus). This coincided with an increased recruitment of FYCO1 on LAMP1-positive structures in maspardin-deficient cells (as shown for GFP-FYCO1 and endogenous FYCO1 in Fig. 9 B and Fig. S3 G, respectively). Moreover, we detected an increase in the colocalization level of FYCO1 with RAB7 (both detected at endogenous levels) in the KO cells (Fig. 9 C, P < 0.001). Taken together, these data indicate that the membrane recruitment of FYCO1 is enhanced by the loss of maspardin, which potentiates the anterograde transport of lysosomes.

## Discussion

It has been known for >20 years that mutations in the *SPG21* gene, which encodes maspardin, cause progressive SPG accompanied by cognitive impairment and ataxia (Simpson et al., 2003). However, without knowing the function of maspardin, the disease mechanisms at the molecular and cellular level have remained unclear.

Our research shows that maspardin deficiency decreases the presentation of the TFEB to the mTOR kinase by the RagC GTPase, resulting in decreased phosphorylation and subsequent nuclear translocation of TFEB. Indeed, overexpression of CA RagC (GDP-bound) corrected TFEB phosphorylation and relocalized it to the cytoplasm in *SPG21* KO cells, as did overexpression of the FLCN/FNIP2 complex (or FNIP2 alone) that activates GTP hydrolysis in RagC and thus activates it. These findings support that the RagC GDP/GTP cycle is perturbed in a way that favors its GTP-bound (inactive) form in *SPG21* KO cells. Interestingly, Kvainickas et al. (2019) found that delocalization of the RAB7 GTPase to nutrient-sensing domains in the lysosomal membrane (induced by retromer dysfunction in this study) alters Rag–Ragulator interaction, impairing mTORC1 activity as a result (Kvainickas et al., 2019). In the present study, we found that maspardin binds to RAB7 in control cells (as previously reported in HEK293T cells (McCray et al., 2010)) and that loss of maspardin expression results in the delocalization of RAB7 molecules from VPS35- and TBC1D5-positive endosomes to LAMP1-, RagC-, and LAMTOR1-positive lysosomes. Similar RAB7 redistribution has been reported in response to a loss of retromer complex or TBC1D5 (RAB7 GAP) expression (Jimenez-Orgaz et al., 2018; Kvainickas et al., 2019), but we did not find any interaction between maspardin and VPS35 or TBC1D5 under conditions showing maspardin’s binding with RAB7. It is unclear whether maspardin belongs to a complex containing these proteins that dissociated during these co-immunoprecipitation experiments or if maspardin independently controls RAB7 localization. Nevertheless, we found that the increased presence of RAB7 on lysosomes in the *SPG21* KO cells led to decreased RAB7 binding with its GAP (likely favoring the GTP-bound state of RAB7) and impaired TFEB phosphorylation by mTOR (which is correctable with a DN form of RAB7 or by silencing *RAB7*). A likely explanation is that RAB7 hyperactivation disrupts the Rag–Ragulator association and subsequent presentation of TFEB to mTOR by RagC. Indeed, Ragulator is known to open the nucleotide-binding pocket of RagC, allowing the release of its GTP (Shen and Sabatini, 2018). Thus, disruption of this release due to the abnormally high presence of RAB7 on the lysosomal membrane could result in RagC remaining in its GTP-bound/inactive form. Of note, we found no interaction between maspardin and RagC in co-immunoprecipitations analyses (data not shown), suggesting that maspardin loss affects RagC GTP/GDP cycle by acting on RAB7 rather than directly on this Rag GTPase. Indeed, overexpression of a DN form of RAB7 or of TBC1D5 in the KO cells provided a rescue effect, consistent with RAB7 being an upstream actor relative to RagC in the regulation of TFEB phosphorylation by mTOR on lysosomes.

Regarding the rescue effect observed after overexpression of FLCN with its partner FNIP2 (or FNIP2 alone, resulting in endogenous FLCN recruitment to lysosomes), it should be noted that FLCN has also been reported to act as a GAP for RAB7, promoting RAB7 inactivation (Laviolette et al., 2017). Therefore, we considered that this rescue might be related to FLCN-dependent GTP hydrolysis in RAB7, rather than in RagC, since RAB7 inactivation and detachment from the membrane in *SPG21* KO cells would cause less disruption of RagC function. However, RAB7 distribution remained punctate/membrane-bound in *SPG21* KO cells after transfection of either FLCN or FNIP2. These results indicate that the rescue effect of FLCN/FNIP2 on TFEB is mainly due to its action on RagC.

It is worth noting that while our article was under revision, the group of Juan Bonifacino published a study also showing that maspardin deficiency, induced by siRNA silencing in HeLa cells, specifically disrupts TFEB phosphorylation by mTOR (not of its other substrates), and that the p.A108P and p.T201Nfs (i.e., c.601insA) mutations in maspardin decrease its expression level (Kunselman et al., 2025). In addition, they reported that folliculin expression is decreased after *SPG21* knockdown, which could result in decreased levels of active RagC. In our *SPG21* KO model, we did not detect changes in folliculin expression or its localization relative to LAMP2 (data not shown). However, it is worth considering that an increased presence of RAB7 on lysosomes could create some competition with RagC for binding to a limited pool of folliculin molecules (even if folliculin does not appear to promote GTP hydrolysis in RAB7).

Interestingly, the delocalization of a pool of GTP-RAB7 to the lysosomes of maspardin-deficient cells also increased the presence of FYCO1, a RAB7 effector involved in anterograde transport, on the lysosomal membrane. The association of this effector with RAB7 might help stabilize it on the lysosomal membrane, as it has been shown that RILP, another RAB7 effector, can stabilize RAB7 in a GTP-bound/active state (Jordens et al., 2001). Whether FYCO1 has a similar stabilizing effect on RAB7 is unclear, but in support of this possibility, RAB7 and FYCO1 co-expression in control HeLa cells increased the colocalization level between RAB7 and LAMP1 compared with cells transfected with RAB7 only (Fig. S3 H). Furthermore, the substantial increase in FYCO1 levels on lysosomes in the maspardin-deficient cells promoted the anterograde trafficking of these vesicles. Together with the recently published finding that maspardin and RAB7 colocalize on immobile and retrograde-moving vesicles in neuronal axons (Kunselman et al., 2025) and with the fact that maspardin preferentially binds to active RAB7 (also recently documented by others (Kunselman et al., 2025; Yan et al., 2022), this observation supports the idea that maspardin is a RAB7 effector that favors retrograde trafficking.

An enhancement of lysosomal transport toward + ends of microtubules has been reported in neurons deficient in SPG48/AP5Z1 (Pierga et al., 2023). This protein and SPG15/spastizin interact with the dynein/dynactin complex and the kinesin KIF13A, respectively. Reduced binding of endo-lysosomes to retrograde trafficking molecules and consequently impaired axonal movement have also been reported in SPG51/AP4E1-deficient neurons (Majumder et al., 2022). Furthermore, anterograde transport of vesicles positive for the late endosomal marker VAMP7 was found to be enhanced in *SPG4* KO cells due to increased kinesin-1–mediated transport (Plaud et al., 2017). These trafficking defects are reminiscent of some aspects of the Charcot-Marie-Tooth type 2B disease, a peripheral axonal neuropathy caused by missense mutations in the *RAB7* gene (Spinosa et al., 2008; McCray et al., 2010; Zhang et al., 2013). Most of the identified mutations underlying this disease are located in the GTP-binding and hydrolysis domains of RAB7, leading to an increase in RAB7 GTP-bound forms due to a deregulated nucleotide exchange rate (Spinosa et al., 2008; McCray et al., 2010). Although the precise mechanisms leading to Charcot-Marie-Tooth type 2B are still debated in the literature, it has been found that this increased association of RAB7 with membranes promotes the anterograde trafficking of lysosomes along axons (Zhang et al., 2013). Moreover, it induces the premature degradation of the NGF/TrkA complexes, which are known to promote neuronal survival and differentiation (Hendry et al., 1974; Grimes et al., 1996; Mulligan and Winckler, 2023). Thus, an alteration of the RAB7 activity cycle can lead to axonal degeneration (Zhang et al., 2013), raising the possibility that it may contribute to the axonal branching defects reported in neuronal models of SPG 21.

Moreover, while TFEB hyperactivation in *SPG21* KO cells only slightly affected the expression of a few lysosomal genes (which is also observed after *SPG21* KD by [Kunselman et al., 2025]), it cannot be excluded that these changes and/or additional consequences of TFEB deregulation would participate to the neuronal defects observed in SPG 21. Indeed, it has been shown that TFEB downregulation in neural stem-progenitor cells leads to their premature differentiation, whereas overexpression of an active form of TFEB maintains these cells in an undifferentiated state (Yuizumi et al., 2021). While the precise mechanisms underlying these effects remain unclear, it is noteworthy that TFEB has been found to have roles in many processes besides lysosomal biogenesis and autophagy, including in cell cycle regulation, senescence, lipid metabolism, and endoplasmic stress response (Franco-Juárez et al., 2022).

**Figure 10.**
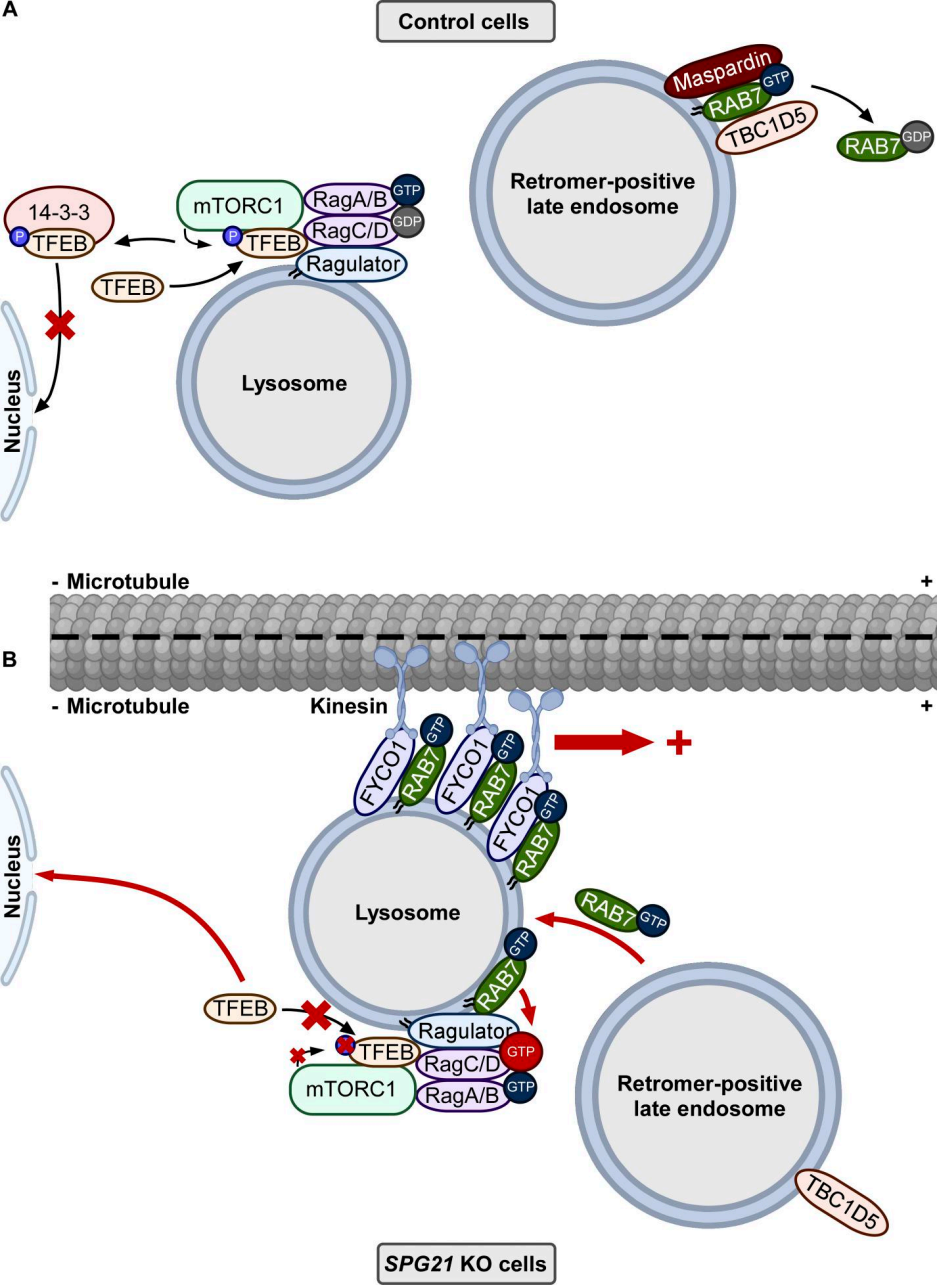
**Proposed working model of the consequences of maspardin deficiency. (A)** In control cells, under basal conditions, Rag GTPases dimer (RagA/B-GTP:RagC/D-GDP) linked to the Ragulator complex is active and enables the recruitment of mTORC1 and TFEB to the lysosomal membrane. The latter is then phosphorylated by the mTOR kinase and trapped in the cytosol by binding to 14-3–3 proteins. In these conditions, RAB7-GTP binds to maspardin on retromer-positive late endosomes where it can be inactivated by association with its GAP TBC1D5, leading to the release of RAB7-GDP into the cytosol. **(B)** In *SPG21* KO HeLa cells, active GTP-bound RAB7 delocalizes from late endosomes to lysosomes. This increases the association of RAB7 with FYCO1 on lysosomes and enhances their microtubule plus-end-directed transport. This increased recruitment of RAB7 also impairs the RagC switch from a GTP-inactive form to a GDP-active form, possibly by disturbing its association with Ragulator. Consequently, TFEB cannot be presented and phosphorylated by the mTOR kinase and thus translocate to the nucleus of the cell. This figure was created using a few elements downloaded from BioRender.com.

In summary, maspardin/SPG21 is a newly discovered RAB7 effector whose deficiency impairs the mTOR–TFEB axis and lysosomal motility (as summarized in the schematics provided in Fig. 10), both of which may potentially contribute to the disease mechanism of SPG 21.

## Materials and methods

### Genetic constructions

pCMV6-entry WT SPG21-myc-flag (NM_016630) was obtained from OriGene. The SPG21 sequence was amplified by PCR using In-Fusion primers (primer sequences provided in Table S1) and inserted using the In-Fusion HD Cloning Kit (Takara Bio) into an EcoRI-digested pcDNA3.1(+) plasmid.

The c.601insA and c.322 C>G (p.A108P) mutations were introduced into the SPG21 sequence by triple PCR (primer sequences provided in Table S1), and the mutated sequences were then integrated using the In-Fusion HD Cloning Kit (Takara Bio) into the pcDNA3.1(+) plasmid linearized using BamHI and BsiWI restriction sites or EcoRI restriction sites, respectively.

pcDNA3.1 Folliculin-His (NM_144997.7), pcDNA3.1 TBC1D5-HA (NM_014744.2), and pcDNA3.1 GFP-TFEB (NM_007162.2) were bought from GenScript.

The following plasmids were obtained from Addgene (https://www.addgene.org/): pRK5 Flag-FNIP2 (RRID:Addgene_72294) from David Sabatini (Czech Academy of Sciences, Prague, Czech Republic) (Tsun et al., 2013); LAMP1-mGFP (RRID: Addgene_34831) from Esteban Dell’Angelica (University of California, Los Angeles, California, USA) (Falcón-Pérez et al., 2005); pRK5 HA-RagA WT (RRID: Addgene_99710), pRK5 HA-RagA CA Q66L (RRID: Addgene_99712), pRK5 HA-RagC WT (RRID: Addgene_99718), and pRK5 HA-RagC CA S75N (RRID: Addgene_99719) were deposited by David Sabatini (Czech Academy of Sciences, Prague, Czech Republic) and Kuang Shen (Whitehead Institute for Biomedical Research and MIT, Cambridge, Massachusetts, USA) (Shen et al., 2017); EGFP-RILP (RRID: Addgene_110498) from Terence Dermody (University of Pittsburgh School of Medicine, Pittsburgh, Pennsylvania, USA) and Bernardo Mainou (Emory University School of Medicine, Atlanta, Georgia, USA) (Mainou and Dermody, 2012); and pGEX-4T-3-RILP (RRID: Addgene_79149) from Aimee Edinger (University of California, Irvine, California, USA) (Romero Rosales et al., 2009). The sequence of RILP was amplified by PCR using In-Fusion primers (primer sequences provided in Table S1) and then inserted into a pET-41b(+) plasmid using In-Fusion HD Cloning Kit (Takara Bio). The pET-41b(+) plasmid was obtained from Novagen and was linearized with BamHI restriction sites.

pDest-EGFP-FYCO1 was a kind gift from Prof. Terje Johansen (The Arctic University of Norway, Tromsø, Norway) (Pankiv et al., 2010), and pCMV6-mCherry-RAB7 WT, mCherry-RAB7 DN (i.e., with a T22N mutation), and mCherry-RAB7 CA (with a Q67L substitution) were generously provided by Dr Stéphanie Miserey (Institut Curie, PSL Research University, Sorbonne Université, Paris, France). These were obtained from GFP-tagged RAB7 constructs (Miserey-Lenkei et al., 2021; Girard et al., 2014) using the Invitrogen Gateway method.

### Engineering of *SPG21* KO HeLa cells

The knockout of *SPG21* in HeLa cells (CCL-2, ATCC, RRID:CVCL_0030) was performed using the CRISPR–Cas9 system. The cells were co-transfected with a puromycin resistance gene (pTERT) and a pX330-U6-Chimeric_BB-CBh-hSpCas9 plasmid obtained from Feng Zhang (Broad Institute of MIT and Harvard, Cambridge, Massachusetts, USA) (RRID: Addgene_42230) (Cong et al., 2013), into which we inserted the sequence encoding a guide RNA targeting exon 2 of *SPG21* (5′-ATA​TGG​TCG​CTC​TAT​GAC​GC-3′ or 5′-CCG​AAG​TAT​CAG​GTG​TCC​TC-3′). The transfected cells were treated with 1.5 µg/ml puromycin for 10 days prior to cloning and screening by sequencing. Two KO clones were selected (one for each guide RNA) to conduct experiments. Two control clones, which did not receive the guide RNA but otherwise underwent the same transfection and selection steps, were also selected.

### Cell culture, transfections, and treatments

HeLa cells were cultured in Dulbecco’s Modified Eagle Medium (DMEM, Biowest) supplemented with 8% Fetal Bovine Serum (FBS, Merck), 100 units/ml of penicillin, and 100 µg/ml of streptomycin (Biowest) at 37°C in a humidified atmosphere with 5% CO_2_. Transfections of plasmids were conducted with FuGENE HD (Promega). Transfection of siRNAs targeting *RAB7* (target sequences: 5′-CUA​GAU​AGC​UGG​AGA​GAU​G-3′; 5′-AAA​CGG​AGG​UGG​AGC​UGU​A-3′; 5′-GAU​GGU​GGA​UGA​CAG​GCU​A-3′; 5′-GGG​AAG​ACA​UCA​CUC​AUG​A-3′, ON-TARGETplus Human RAB7A siRNA, L-018225-00, Horizon), or non-targeting siRNAs (target sequences: 5′-UGG​UUU​ACA​UGU​CGA​CUA​A-3′; 5′-UGG​UUU​ACA​UGU​UGU​GUG​A-3′; 5′-UGG​UUU​ACA​UGU​UUU​CUG​A-3′; 5′-UGG​UUU​ACA​UGU​UUU​CCU​A-3′, ON-TARGETplus Non-targeting Pool, Horizon), was conducted using Lipofectamine RNAiMax (Invitrogen). Analyses were conducted 24, 48, or 72 h after transfection, as described in the figure legends.

When indicated, the cells were treated for 3 h with 5 µM of FK506 (Bio-Techne) and 10 µM of Cyclosporin A (Merck) or with 1 µM of MG132 (Merck) for 16 h.

### Subcellular fractionation

Cells were homogenized in ice-cold 0.25 M sucrose with six passages in a tight Dounce homogenizer. A sample of the cell homogenate was kept for further analysis, while the remaining volume was centrifuged at 1,400 *g* during 2 min at 4°C in a Optima TLX Ultracentrifuge (Beckman) using a TLA100.3 rotor. The supernatant, i.e., the postnuclear supernatant fraction was collected, and the pellet representing the nuclear fraction (N) was resuspended in 0.25 M sucrose. Equal amounts of proteins for the homogenate and for the two subcellular fractions were then mixed with Laemmli’s sample buffer containing 100 mM dithiothreitol, heated at 95°C during 5 min and analyzed by western blotting as described below.

### Co-immunoprecipitation

When endogenous maspardin was used as bait, cells were lysed in ice-cold buffer containing 120 mM NaCl, 50 mM Tris-HCl, pH 7.4, 0.05% NP40, and 5 mM EDTA supplemented with protease inhibitors (cOmplete Tablets, Mini, Roche) for 40 min at 4°C on a rotating wheel and incubated with 1 µg of anti-maspardin or non-targeting antibodies for 4 h at 4°C on a rotating wheel as well. Antibody references are provided in Table S2. Protein A–coated magnetic beads (Invitrogen) were used for the precipitation step. Washes were conducted with PBS Tween 0.05% (without protease inhibitors). Proteins were eluted using Laemmli’s sample buffer containing 200 mM dithiothreitol and heated at 95°C during 5 min. Samples were analyzed by western blotting as described below.

When using mCherry-RAB7 as the bait, the cells were pretreated with a cross-linking agent (500 µM DSP, Merck) for 30 min at RT and then lysed in 150 mM NaCl, 30 mM Tris-HCl, pH 7.5, 0.5% NP40, and 1 mM MgCl_2_ (containing protease inhibitors). The lysates were then incubated with red fluorescent protein–trap beads (Proteintech) for 1 h 30 at 4°C on a rotating wheel. Afterward, the beads were washed three times and eluted as described, except for maspardin coprecipitation experiments, for which the NaCl concentration was increased to 500 mM in the wash buffer.

### GST pull-down assay

For the production of GST-RILP proteins, GST-RILP in pET-41b(+) was transformed into *Escherichia coli* strain BL21. Bacteria were cultured in LB broth at 37°C and grown to an OD of 0.6–0.8. Protein production was then induced by incubating the bacterial culture with isopropyl β-d-thiogalactoside (Merck) to a final concentration of 0.5 mM for 4–5 h at 30°C. After a centrifugation step, bacteria were lysed in ice-cold PBS containing 0.5% NP-40, 0.5% Triton X-100, and proteases inhibitor cocktail (cOmplete Tablets, Mini, Roche) and then sonicated for 40 s. Incubation on a rotating wheel with glutathione-agarose beads for 2 h 30 at RT was implemented to isolate the GST fusion protein. After four washes with PBS and one wash with Tris-HCl 0.05 M (pH 8), GST-RILP proteins were eluted using 10 mM L-glutathione reductase (Merck). Proteins were dialyzed with ice-cold PBS before use.

For the RAB7 pull-down assay, HeLa cells were lysed in an ice-cold pull-down buffer containing 20 mM HEPES, pH 7.4, 100 mM NaCl, 5 mM MgCl_2,_ 1% Triton X-100, and proteases inhibitor cocktail (cOmplete Tablets, Mini, Roche) for 45 min at 4°C on a rotating wheel. 30 µg of GST-RILP–purified proteins were bound to 80 μl of glutathione-agarose beads for 1 h at RT on a rotating wheel, prior to washes with the pull-down buffer and addition of the cell lysates. After 3 h 30 incubation at 4°C on a rotating wheel, several washes were conducted, and proteins were eluted with Laemmli’s sample buffer containing 200 mM dithiothreitol and by heating at 95°C for 5 min. Input material and the elution fraction were analyzed by western blotting as detailed below.

### Western blotting

Cellular proteins were extracted using ice-cold PBS–Triton X-100 1% supplemented with protease (cOmplete Tablets, Mini, Roche) and phosphatase (PhosSTOP, Roche) inhibitors, then mixed with Laemmli’s sample buffer containing 100 mM dithiothreitol, and heated at 95°C during 5 min. Proteins were then resolved by sodium dodecyl sulfate-polyacrylamide gel electrophoresis (SDS-PAGE) under reducing conditions and transferred on a low-fluorescence polyvinylidene fluoride membrane (Immobilon-FL, Merck) using a Mini-PROTEAN Tetra system (BIO-RAD). The membrane was then incubated with PBS-0.1% Tween 20 containing 5% fat-free milk or in TBS-0.1% Tween 20 containing either 5% fat-free milk or 5% BSA depending on the antibody, as documented in Table S2. Proteins of interest were then detected with primary antibodies diluted in the same solution (references and dilution factors are listed in Table S2), followed by incubation with corresponding IRDye-coupled secondary antibodies (references and dilution factors are listed in Table S2). Infrared signals were detected by Amersham Typhoon Biomolecular Imager. For p70S6K and p-p70S6K detection, the membrane was incubated with an HRP-coupled secondary antibody (references and dilution factors are listed in Table S2), and signals were revealed using chemiluminescence (Western Lightning Plus–ECL, Perkin-Elmer). Signals were quantified using Fiji (RRID:SCR_002285) software (version 1.54f), and data were expressed as fold change. When two control and two KO clones were included in the analyses, one of the control clones was set to 1.

### Immunofluorescence analyses

Cells were grown on 15-mm glass coverslips and then fixed for 10 min in 4% paraformaldehyde diluted in PBS, pH 7.4, at RT. After blocking and cell permeabilization for 1 h at RT using PBS containing 0.02% saponin and 3% BSA, the cells were incubated for 1 h at RT with a primary antibody diluted in the same buffer (references and dilution factors provided in Table S2). The cells were then washed with PBS and incubated for 50 min in the dark at RT with Alexa Fluor–coupled secondary antibodies (references and dilution factors are listed in Table S2). When indicated, nuclei were stained with DAPI (Merck). Lastly, the coverslips were mounted using Mowiol 40–88 (Merck). For filipin staining, the cells were fixed in 3% paraformaldehyde diluted in PBS, pH 7.4, at RT and then incubated for 2 h with a 40 µg/μl filipin III solution (Merck) prior to mounting with Mowiol 40–88 (Merck).

Micrographies were acquired at RT using a Leica TCS SP5 Broadband Confocal Laser Scanning Microscope, operated with the Leica Application Suite software. Imaging was performed using either a 40× or a 63× oil immersion objective, with 1.3 or 1.4 numerical apertures, respectively. Type F immersion liquid (Leica) was used as imaging medium. In the same experiment, micrographies were similarly processed using FIJI software (version 1.54f) to optimize brightness.

### Micrography analyses and quantifications

The four clones (two controls and two KO) were included in all experiments. Micrographies were quantified using the (Fiji Is Just) ImageJ software (version 1.54f), as described hereafter. Ten cells per clone were quantified in each biological replicate, with at least three independent experiments. In each experiment, the average value for each clone was calculated, and statistical analyses were performed on these averages.

#### Colocalization quantification

Quantification of colocalization between two channels was performed by measuring the Manders’ overlap coefficient using the JACoP plugin. For the quantification of filipin in LAMP1-GFP–positive structures, a mask was created for LAMP1-GFP–positive structures. Relative filipin fluorescence intensity in lysosomes (expressed as a percentage) was calculated by dividing the fluorescence intensity of filipin detected in the LAMP1-GFP mask by the total fluorescence intensity of filipin in the whole cell.

#### RAB7 granularity quantification

RAB7 granularity was determined by counting the number of particles in each cell (using the “Analyze particle” function of Fiji) after setting a threshold to distinguish membrane RAB7 from cytosolic RAB7 to exclude cytosolic signal.

#### TFEB nuclear translocation quantification

TFEB nuclear translocation was quantified by two different methods. First, it was quantified by determining the number of cells (expressed as a percentage) in which TFEB was predominantly localized in the nucleus.

TFEB nuclear translocation was also quantified by calculating the TFEB nuclear/cytoplasmic signal ratio after measuring TFEB fluorescence intensity in the nucleus and cytoplasm.

#### Lysosome number quantification

The number of lysosomes was determined by counting the number of particles in each cell after creating a binary mask of LAMP1 signals and applying the watershed separation technique to divide connected components into individual ones.

#### Lysosome distribution quantification

Quantification of lysosome distribution was performed by measuring LAMP1 fluorescence intensity in four areas of the cell. After drawing a region of interest (ROI) around the nucleus and removing the potential LAMP1 signal present in the nuclear area, this ROI was enlarged by 5 µm to define an area from 0 to 5 µm from the nucleus. This ROI was then enlarged two more times at 5-µm intervals to define areas 5–10, 10–15, and >15 µm from the nucleus. LAMP1 fluorescence intensity was then measured in each ROI. Lysosome distribution (expressed as a percentage of total LAMP1 fluorescence intensity in the cell) was determined by dividing the LAMP1 fluorescence intensity of each ROI by the LAMP1 fluorescence intensity of the whole cell.

### RNA extraction and RT–quantitative PCR analyses

Total RNA was extracted from HeLa cells with NucleoSpin RNA Plus kit (Macherey-Nagel), following the manufacturer’s instructions and reverse transcribed into cDNA using RevertAid H Minus First Strand cDNA Synthesis Kit (Thermo Fisher Scientific). A mixture containing 1 ng/μl cDNA, 2.4 µM of forward and reverse primers (Eurogentec, Table S1), and Takyon No ROX SYBR MasterMix blue dTTP (Eurogentec) was prepared for quantitative PCR (qPCR). The qPCR was carried out using a CFX96 real-time PCR detection system (Bio-Rad). *GAPDH* expression was used as a housekeeping gene, and the 2^−ΔΔCq^ method was used to calculate the relative gene expression.

### Statistical analyses

Data are represented as means ± SD. GraphPad Prism software (version 10.3.1) (RRID:SCR_002798) was used to perform the statistical analysis using two-tailed paired or unpaired *t* test, as described in figure legends. Data distribution was assumed to be normal, but this was not formally tested. A P value <0.05 was considered as statistically significant (*P < 0.05; **P < 0.01; ***P < 0.001; ****P < 0.0001).

### Online supplemental material

Fig. S1 shows cholesterol labelling in lysosomes of control and *SPG21* KO HeLa cells and the unchanged pTFEB/TFEB ratio when these cells were treated with calcineurin inhibitors. Fig. S2 shows pTFEB/TFEB ratios in control cells after transfection of RagA, RagC, FLCN/FNIP, or TBC1D5 constructs, as well as the membrane recruitment of endogenous folliculin when Flag-FNIP2 is overexpressed. Fig. S3 shows the basal expression levels of RAB7, VPS35, and TBC1D5 in control and *SPG21* KO cells; several colocalization analyses (TBC1D5-VPS26, VPS35-LAMP1, and FYCO1-LAMP1 in control and KO cells; RAB7-VPS35 in control cells after overexpression of maspardin-myc-flag; RAB7-LAMP1 in control cells after overexpression of GFP-FYCO1); and the coprecipitation analysis of CCZ1 by RAB7 in control and *SPG21* KO cells. Table S1 lists the primers used for the generation of genetic constructs and for qPCR analyses. Table S2 lists all the antibodies used in this study and dilution factors.

## Supplementary Material

Table S1 shows the list of primers used for the generation of new genetic constructs and for qPCR analyses.

Table S2 shows the list of primary and secondary antibodies used in this study.

SourceData F1 is the source file for Fig. 1.

SourceData F2 is the source file for Fig. 2.

SourceData F3 is the source file for Fig. 3.

SourceData F4 is the source file for Fig. 4.

SourceData F5 is the source file for Fig. 5.

SourceData F6 is the source file for Fig. 6.

SourceData F7 is the source file for Fig. 7.

SourceData FS1 is the source file for Fig. S1.

SourceData FS2 is the source file for Fig. S2.

SourceData FS3 is the source file for Fig. S3.

## Data Availability

All data used in this article are included within the figures and supplementary material files.
