# Supplementary material for: Maspardin/SPG21 controls lysosome motility and TFEB phosphorylation through RAB7 positioning
Source: J Cell Biol. 2025 Dec 16;225(2):e202501135. doi: 10.1083/jcb.202501135 (PMC12707310; doi:10.1083/jcb.202501135)
Supplement: Table S1 — shows the list of primers used for the generation of new genetic constructs and for qPCR analyses. [file jcb_202501135_tables1.docx]

|  | **Forward primer** | **Reverse primer** |
| --- | --- | --- |
| SPG21_InFusion | 5'-CAGTGTGGTGGAATTCATGGGAGAGATTAAAGTCTCT-3' | 5'-GATATCTGCAGAATTCGTTTAAACCTTATCGTCGTCA-3' |
| SPG21_601insA_InFusion | 5'-GGTACCGAGCTCGGATCCACTAGTCCAGTGTGGTGG-3' | 5'-CGAGCGGCCGCGTACGTGAGGTTCCACATAAGAATT-3' |
| SPG21_601insA | 5'-CTTCAAGACTTAACCTTGAATTG-3' | 5'-CAATTCAAGGTTAAGTCTTGAAG-3' |
| SPG21_A108P | 5'-TCTTTTTGGCCCTTCTTTGG-3' | 5'-CCAAAGAAGGGCCAAAAAGA-3' |
| RILP_InFusion | 5’-TACAGAATTCGGATCCTCAGCCATCCTCATCCTCACTG-3’ | 5’-TGGATATCGGGGATCCATGTGGATTCAGCAGGGAAGAGC-3’ |
| *CTSB* | 5'-AGTGGAGAATGGCACACCCTA-3' | 5'-AAGAAGCCATTGTCACCCCA-3' |
| *CTSC* | 5'-AGGAGGTTGTGTCTTGTAGCC-3' | 5'-AGTGCCTGTGTAGGGGAAGC-3' |
| *CTSD* | 5'-ATCTCCGTCAACAACGTGCT-3' | 5'-TGGGTCCCTGCTCAGGTAGA-3' |
| *GAPDH* | 5'-GAAGGTGAAGGTCGGAGT-3' | 5'-GAAGATGGTGATGGGATTTC-3' |
| *GUSB* | 5'-GAAAATATGTGGTTGGAGAGCTCATT-3' | 5'-CCGAGTGAAGATCCCCTTTTTA-3' |
| *HEXA* | 5'-TTCCCATATGAGAGCTTCAC-3' | 5'-TATTGAGACTGGGATTCACT-3' |
| *HEXB* | 5'-TTGGGAGGAGATGAAGTGG-3' | 5'-AAACCTCCTGCCAGACAATG-3' |
| *LAMP1* | 5'-ATGTGTTAGTGGCACCCAGG-3' | 5'-TGTTCACAGCGTGTCTCTCC-3' |
| *LAMP2* | 5'-GCAGCTGAACATCACTCAGG-3' | 5'-CACAGCAAAGACAAAGTCTAG-3' |
| *MANBA* | 5'-TGAATGCCATGTCAACTTTGT-3' | 5'-TGTGTTTGCAACTTAGGGAT-3' |

Supplementary Table 1: **List of primers used for the generation of new genetic constructs and for qPCR analyses.**
