# Supplementary material for: Maspardin/SPG21 controls lysosome motility and TFEB phosphorylation through RAB7 positioning
Source: J Cell Biol. 2025 Dec 16;225(2):e202501135. doi: 10.1083/jcb.202501135 (PMC12707310; doi:10.1083/jcb.202501135)
Supplement: Table S2 — shows the list of primary and secondary antibodies used in this study. [file jcb_202501135_tables2.docx]

| **Antibody** | **Supplier** | **Reference** | **Dilution** | |
| --- | --- | --- | --- | --- |
|  |  |  | **Western blotting** | **Immunofluorescence (dilution in PBS, 3% BSA, 0.02% Saponin)** |
| **Primary antibodies** | | | | |
| CCZ1 | Santa Cruz | SC-514290 | 1/250 in PBS-0.1% Tween 20 with 5% fat-free milk | / |
| Flag tag | Merck | F1804 (RRID:AB_262044) | 1/1000 in TBS-0.1% Tween 20 containing 5% fat-free milk | 1/100 |
| FYCO1 | Abnova | ABNOH00079443-A01 | / | 1/200 |
| 6X His tag | Invitrogen | MA5 33032 (RRID:AB_2810125) | / | 1/100 |
| 6X His tag | Abcam | ab18184 (RRID:AB_444306) | 1/1000 in PBS-0.1% Tween 20 with 5% fat-free milk | / |
| GAPDH | Merck | G8795 (RRID:AB_1078991) | 1/3000 in PBS-0.1% Tween 20 with 5% fat-free milk | / |
| Folliculin | Cell Signaling | 3697 | / | 1/100 |
| HA tag | Cell Signaling Technology | 3724 (RRID:AB_1549585) | / | 1/100 |
| HA tag | Cell Signaling Technology | 2367 (RRID:AB_10691311) | 1/1000 in TBS-0.1% Tween 20 containing 5% fat-free milk | / |
| Histone H1 | Abcam | ab125027 (RRID:AB_11000797) | 1/1000 in PBS-0.1% Tween 20 with 5% fat-free milk | / |
| LAMP1 | Cell Signaling Technology | 9091 (RRID:AB_2687579) | / | 1/100 |
| LAMP1 | DSHB | H4A3 (RRID:AB_2296838) | / | 1/50 |
| LAMP2 | DSHB | H4B4 (RRID:AB_2134755) | / | 1/100 |
| LAMTOR1 | Cell Signaling | 8975S | / | 1/100 |
| Maspardin | Abcam | ab220229 | 1/2000 in PBS-0.1% Tween 20 with 5% fat-free milk | 1/100 |
| mCherry tag | Cell Signaling | 43590 (RRID:AB_2799246) | 1/1000 in TBS-0.1% Tween 20 containing 5% BSA | / |
| mTOR | Cell Signaling Technology | 2983 (RRID:AB_2105622) | / | 1/50 |
| Myc tag | Cell Signaling Technology | 2272 (RRID:AB_10692100) | 1/1000 in TBS-0.1% Tween 20 containing 5% fat-free milk | / |
| Myc tag | Cell Signaling Technology | 2276 (RRID:AB_331783) | 1/1000 in TBS-0.1% Tween 20 containing 5% fat-free milk | 1/100 |
| p70S6K | Cell Signaling Technology | 9202 (RRID:AB_331676) | 1/1000 in TBS-0.1% Tween 20 containing 5% BSA | / |
| p-p70 S6K | Cell Signaling Technology | 9206 (RRID:AB_2285392) | 1/750 in TBS-0.1% Tween 20 containing 5% fat-free milk | / |
| pTFEB S211 | Cell Signaling Technology | 37681 (RRID:AB_2799117) | 1/500 in TBS-0.1% Tween 20 containing 5% BSA | / |
| RAB7 | Santa Cruz | sc-376362 | 1/250 in PBS-0.1% Tween 20 with 5% fat-free milk | / |
| RAB7 | Abcam | ab137029 (RRID:AB_2629474) | 1/1000 in TBS-0.1% Tween 20 containing 5% fat-free milk | 1/100 |
| tdTomato tag | SICGEN | ab8181-200 | 1/1000 in PBS-0.1% Tween 20 with 5% fat-free milk | / |
| TBC1D5 | Santa Cruz | SC-376296 (RRID:AB_10988434) | 1/500 in PBS-0.1% Tween 20 with 5% fat-free milk | 1/100 |
| TFEB | Bethyl Laboratories | A303-673A | 1/2000 in PBS-0.1% Tween 20 with 5% fat-free milk | / |
| α-Tubulin | Merck | T5168 (RRID:AB_477579) | 1/3000 in PBS-0.1% Tween 20 with 5% fat-free milk | / |
| VPS35 | Abcam | ab10099 (RRID:AB_296841) | 1/500 in PBS-0.1% Tween 20 with 5% fat-free milk | 1/75 |
| VPS26 | Abcam | ab181352 (RRID:AB_2665924) | / | 1/100 |
| **Secondary antibodies** | | | | |
| Alexa Fluor 488 goat anti-rabbit IgG | Thermo Fisher Scientific | A-11034  (RRID:AB_2576217) | / | 1/1000 |
| Alexa Fluor 488 goat anti-mouse IgG | Thermo Fisher Scientific | A-11001  (RRID:AB_2534069) | / | 1/1000 |
| Alexa Fluor 488 donkey anti-mouse IgG | Thermo Fisher Scientific | A-21202  (RRID:AB_141607) | / | 1/1000 |
| Alexa Fluor 488 donkey anti-goat IgG | Thermo Fisher Scientific | A-11055  (RRID:AB_2534102) | / | 1/1000 |
| Alexa Fluor 568 goat anti-rabbit IgG | Thermo Fisher Scientific | A-11011  (RRID:AB_143157) | / | 1/1000 |
| Alexa Fluor 568 goat anti-mouse IgG | Thermo Fisher Scientific | A-11004  (RRID:AB_2534072) | / | 1/1000 |
| Alexa Fluor 568 donkey anti-goat IgG | Thermo Fisher Scientific | A-11057  (RRID:AB_2534104) | / | 1/400 |
| Alexa Fluor 594 donkey anti-rabbit IgG | Thermo Fisher Scientific | A-21207  (RRID:AB_141637) | / | 1/400 |
| Alexa Fluor 633 goat anti-mouse IgG | Thermo Fisher Scientific | A-21050  (RRID:AB_2535718) | / | 1/400 |
| Alexa Fluor 647 donkey anti-rabbit IgG | Thermo Fisher Scientific | A-31573  (RRID:AB_2536183) | / | 1/400 |
| IRDYE 800CW goat anti-rabbit IgG | LICORbio | 926-32211  (RRID:AB_621843) | 1/10000 in the same buffer as primary antibody | / |
| IRDYE 800CW donkey anti-goat IgG | LICORbio | 926-32214  (RRID:AB_621846) | 1/10000 in the same buffer as primary antibody | / |
| IRDYE 800CW goat anti-mouse IgG | LICORbio | 926-32210  (RRID:AB_621842) | 1/10000 in the same buffer as primary antibody | / |
| IRDYE 680RD goat anti-mouse IgG | LICORbio | 926-68070  (RRID:AB_10956588) | 1/10000 in the same buffer as primary antibody | / |
| IRDYE 680RD goat-anti-rabbit IgG | LICORbio | 926-68071  (RRID:AB_10956166) | 1/10000 in the same buffer as primary antibody | / |
| HRP-linked anti-rabbit IgG | Cell Signaling Technology | 7074  (RRID:AB_2099233) | 1/2000 in the same buffer as primary antibody | / |
| HRP-linked anti-mouse IgG | Cell signaling Technology | 7076  (RRID:AB_330924) | 1/2000 in the same buffer as primary antibody | / |

Supplementary Table 2: **List of primary and secondary antibodies used in this study.**
