# Supplementary material for: Maspardin/SPG21 controls lysosome motility and TFEB phosphorylation through RAB7 positioning
Source: J Cell Biol. 2025 Dec 16;225(2):e202501135. doi: 10.1083/jcb.202501135 (PMC12707310; doi:10.1083/jcb.202501135)
Supplement: SourceData F1 — is the source file for Fig. 1. [file jcb_202501135_sourcedataf1.pdf]

Figure 1A

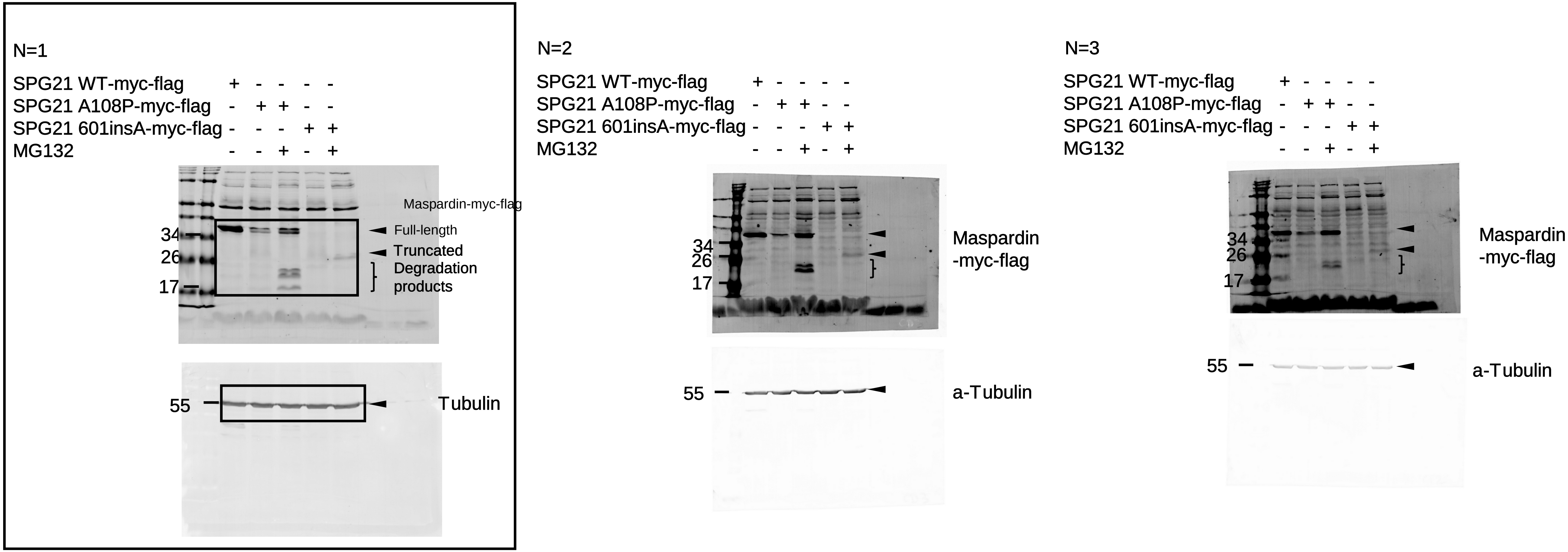

Set shown in the article

Figure 1B

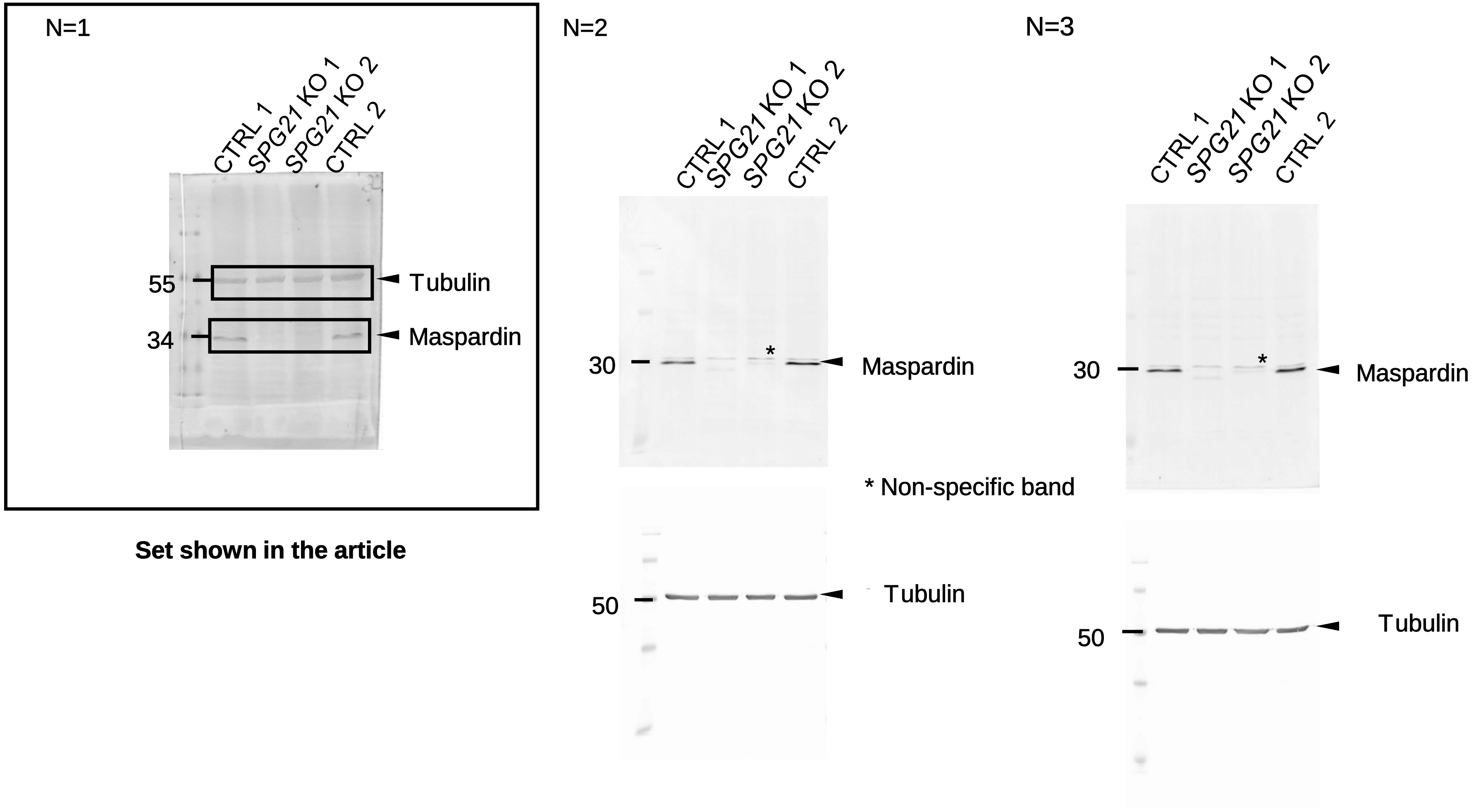

Set shown in the article

Figure 1D N= 1

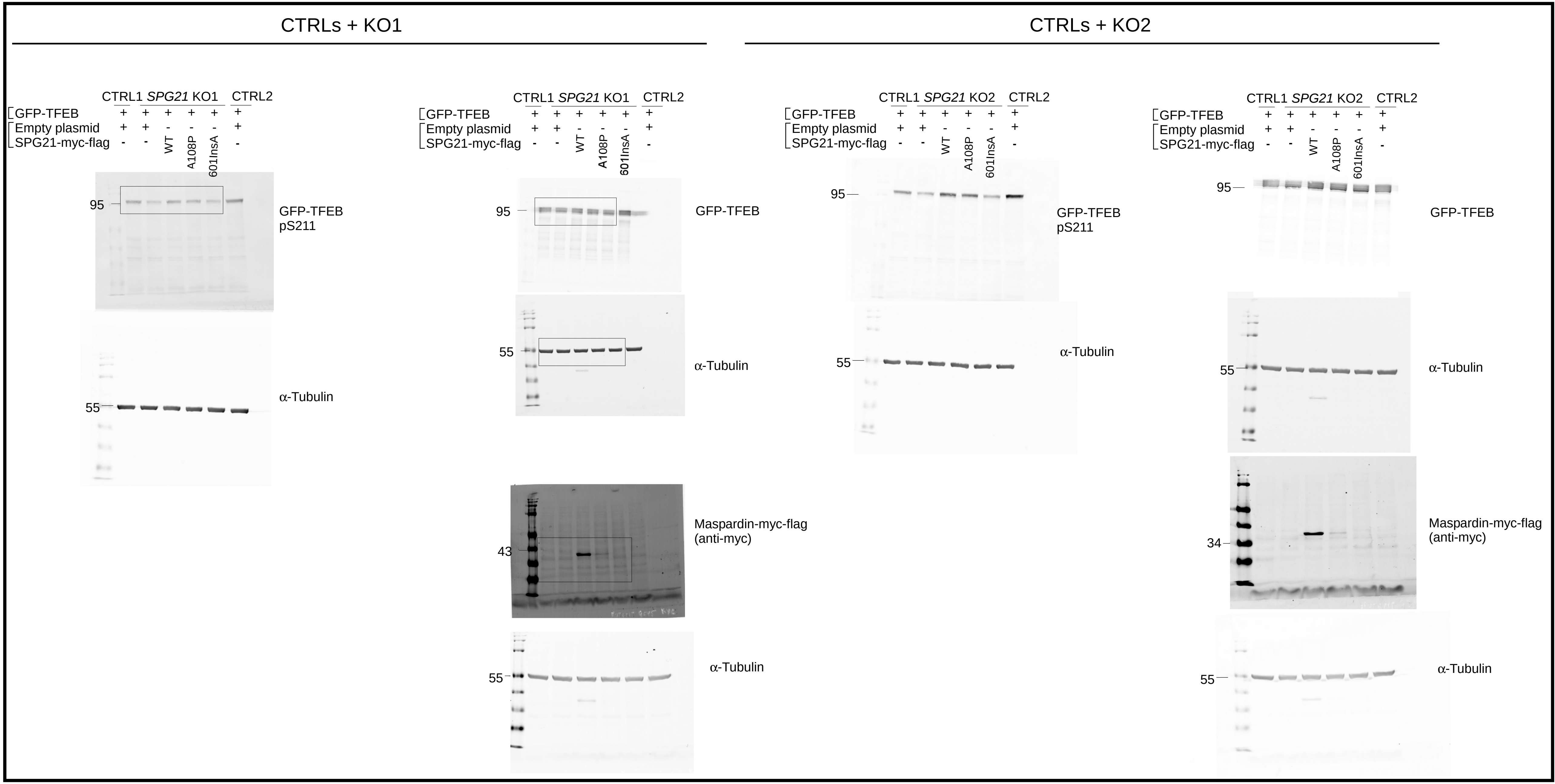

Data shown in the article extracted from this set

Figure 1D N= 2

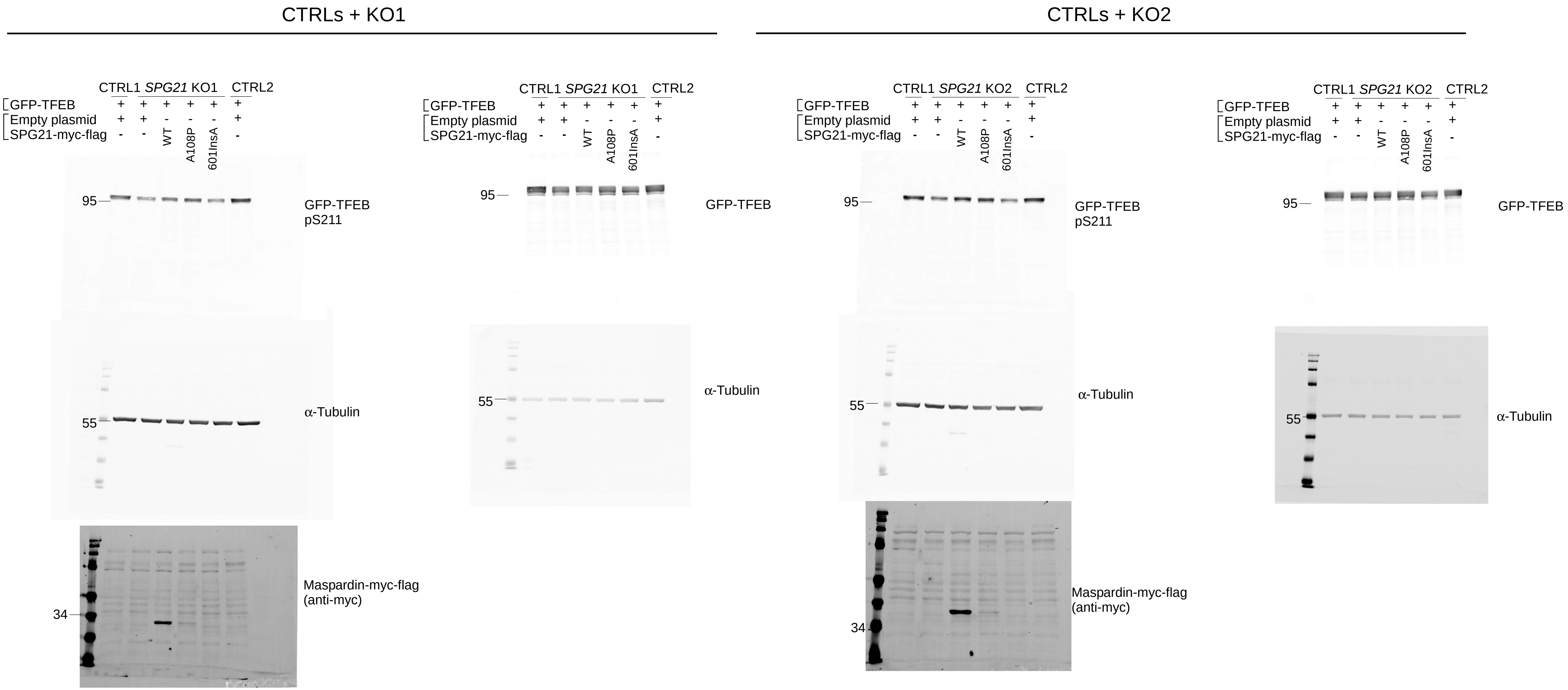

CTRLs + KO1
CTRLs + KO2
CTRL1 SPG21 KO1 CTRL2
GFP-TFEB + + + + + +
Empty plasmid + + - - - +
SPG21-myc-flag - - WT A108P 601InsA -
95
GFP-TFEB pS211
55
α-Tubulin
34
Maspardin-myc-flag (anti-myc)
CTRL1 SPG21 KO1 CTRL2
GFP-TFEB + + + + + +
Empty plasmid + + - - - +
SPG21-myc-flag - - WT A108P 601InsA -
95
GFP-TFEB
55
α-Tubulin
CTRL1 SPG21 KO2 CTRL2
GFP-TFEB + + + + + +
Empty plasmid + + - - - +
SPG21-myc-flag - - WT A108P 601InsA -
95
GFP-TFEB pS211
55
α-Tubulin
34
Maspardin-myc-flag (anti-myc)
CTRL1 SPG21 KO2 CTRL2
GFP-TFEB + + + + + +
Empty plasmid + + - - - +
SPG21-myc-flag - - WT A108P 601InsA -
95
GFP-TFEB
55
α-Tubulin

Figure 1D N= 3

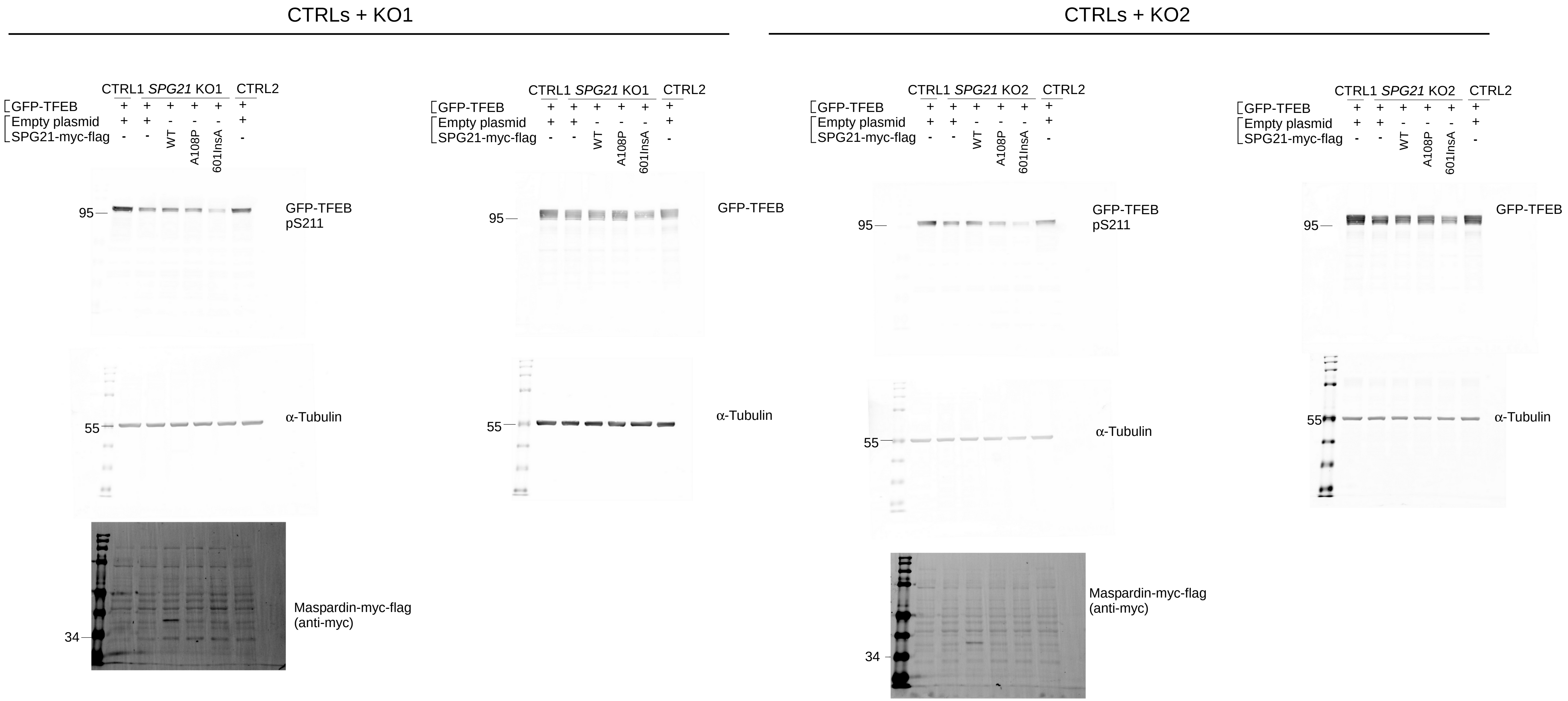

CTRLs + KO1
CTRLs + KO2
CTRL1 SPG21 KO1 CTRL2
CTRL1 SPG21 KO2 CTRL2
GFP-TFEB + + + + + +
Empty plasmid + + - - - +
SPG21-myc-flag - - WT A108P 601InsA -
95
55
34
GFP-TFEB pS211
GFP-TFEB
α-Tubulin
Maspardin-myc-flag (anti-myc)

Figure 1F

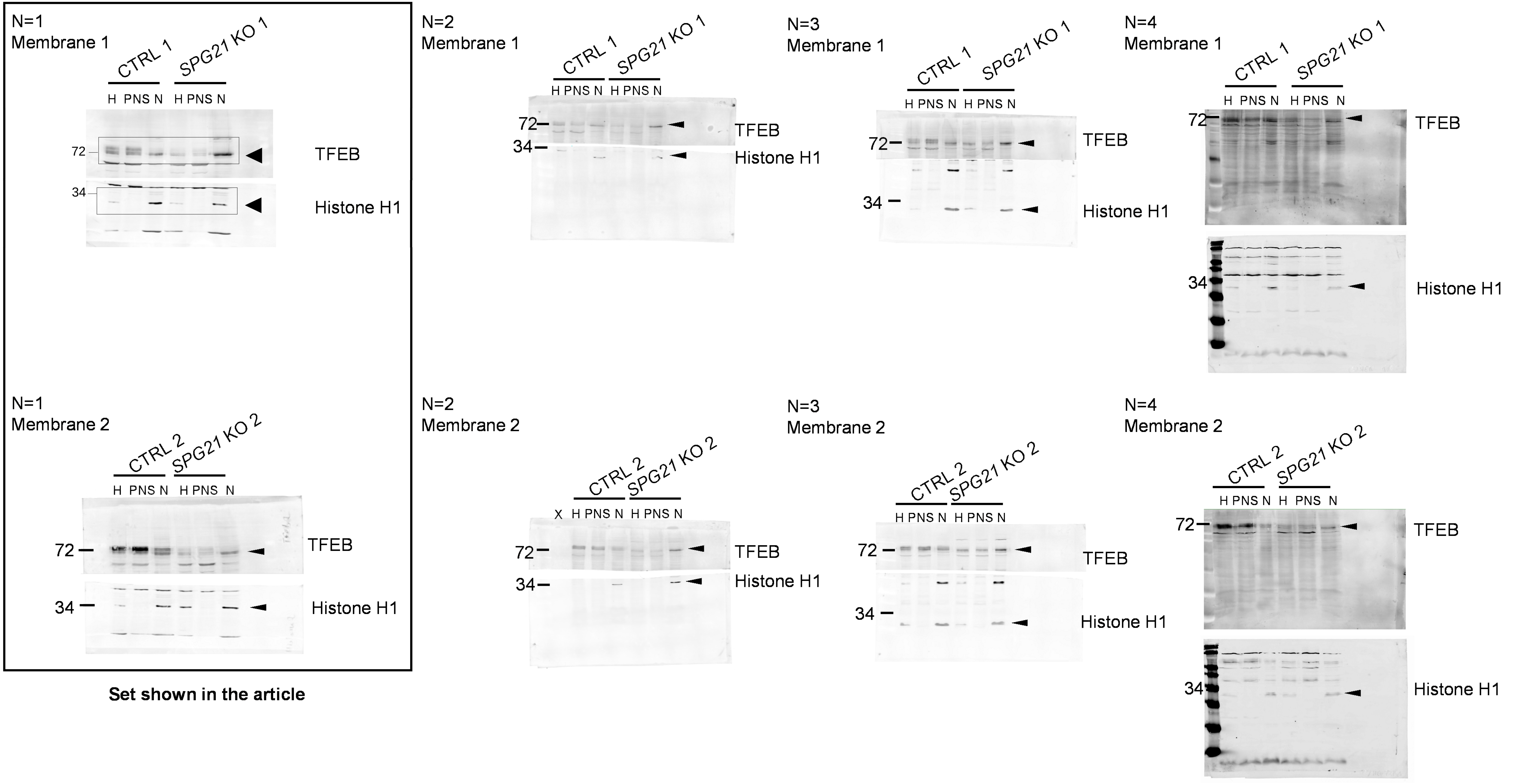

Set shown in the article

Figure 1F

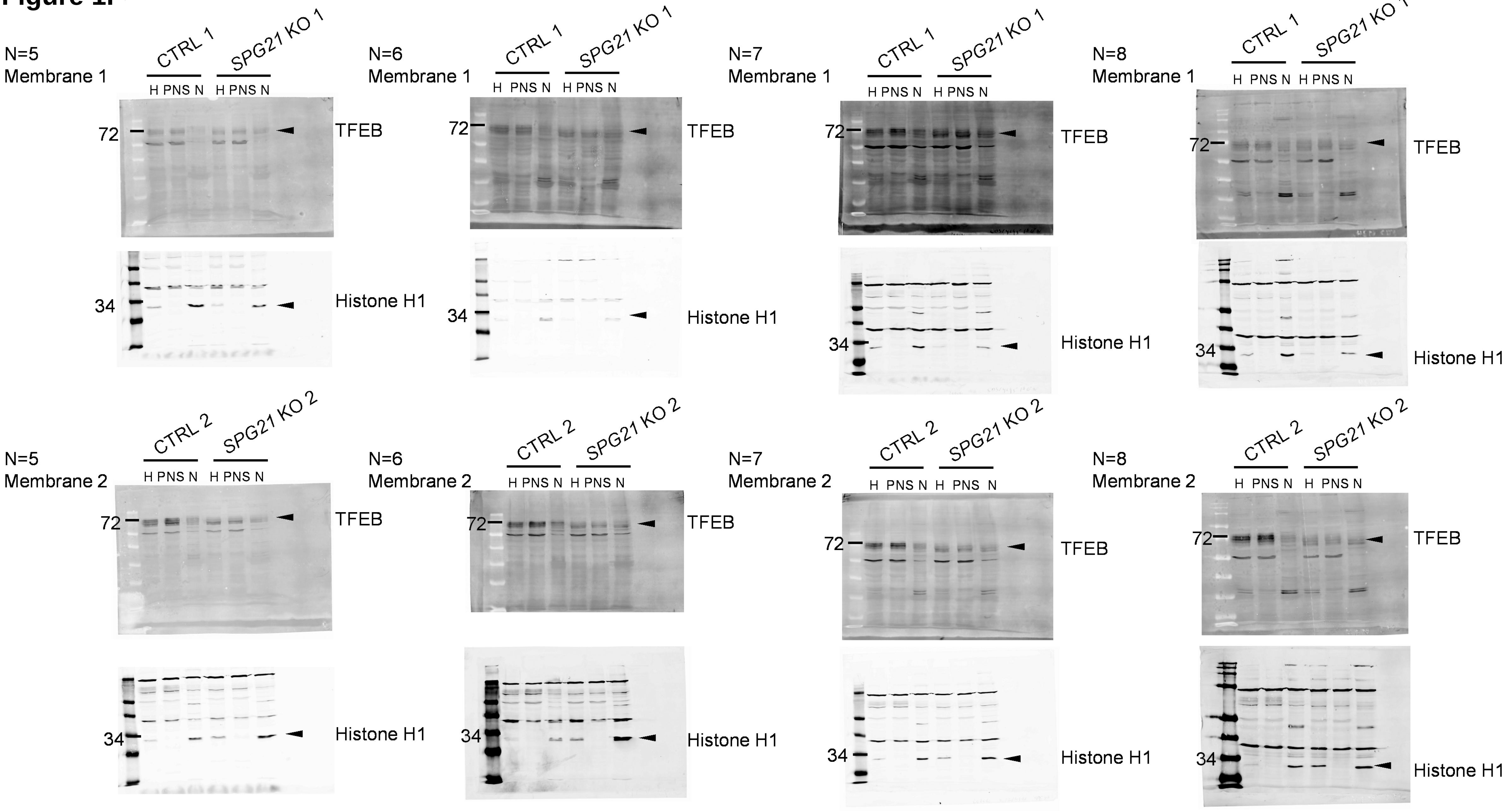
N=5
Membrane 1
CTRL 1
SPG21 KO 1
H PNS N H PNS N
72
TFEB
34
Histone H1
N=6
Membrane 1
CTRL 1
SPG21 KO 1
H PNS N H PNS N
72
TFEB
34
Histone H1
N=7
Membrane 1
CTRL 1
SPG21 KO 1
H PNS N H PNS N
72
TFEB
34
Histone H1
N=8
Membrane 1
CTRL 1
SPG21 KO 1
H PNS N H PNS N
72
TFEB
34
Histone H1
N=5
Membrane 2
CTRL 2
SPG21 KO 2
H PNS N H PNS N
72
TFEB
34
Histone H1
N=6
Membrane 2
CTRL 2
SPG21 KO 2
H PNS N H PNS N
72
TFEB
34
Histone H1
N=7
Membrane 2
CTRL 2
SPG21 KO 2
H PNS N H PNS N
72
TFEB
34
Histone H1
N=8
Membrane 2
CTRL 2
SPG21 KO 2
H PNS N H PNS N
72
TFEB
34
Histone H1
