# Supplementary material for: Maspardin/SPG21 controls lysosome motility and TFEB phosphorylation through RAB7 positioning
Source: J Cell Biol. 2025 Dec 16;225(2):e202501135. doi: 10.1083/jcb.202501135 (PMC12707310; doi:10.1083/jcb.202501135)
Supplement: SourceData F2 — is the source file for Fig. 2. [file jcb_202501135_sourcedataf2.pdf]

Figure 2B

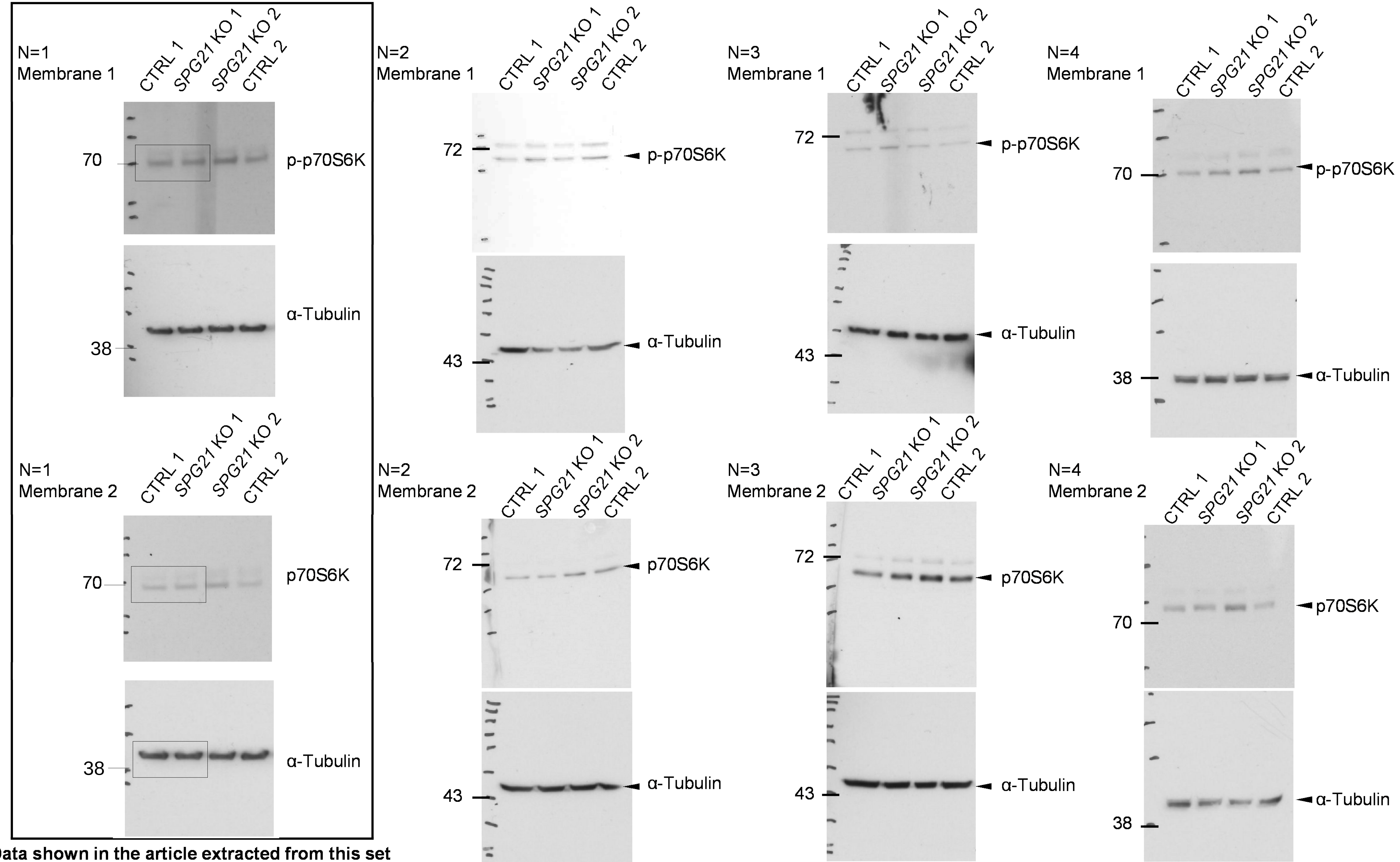

Data shown in the article extracted from this set

Figure 2B

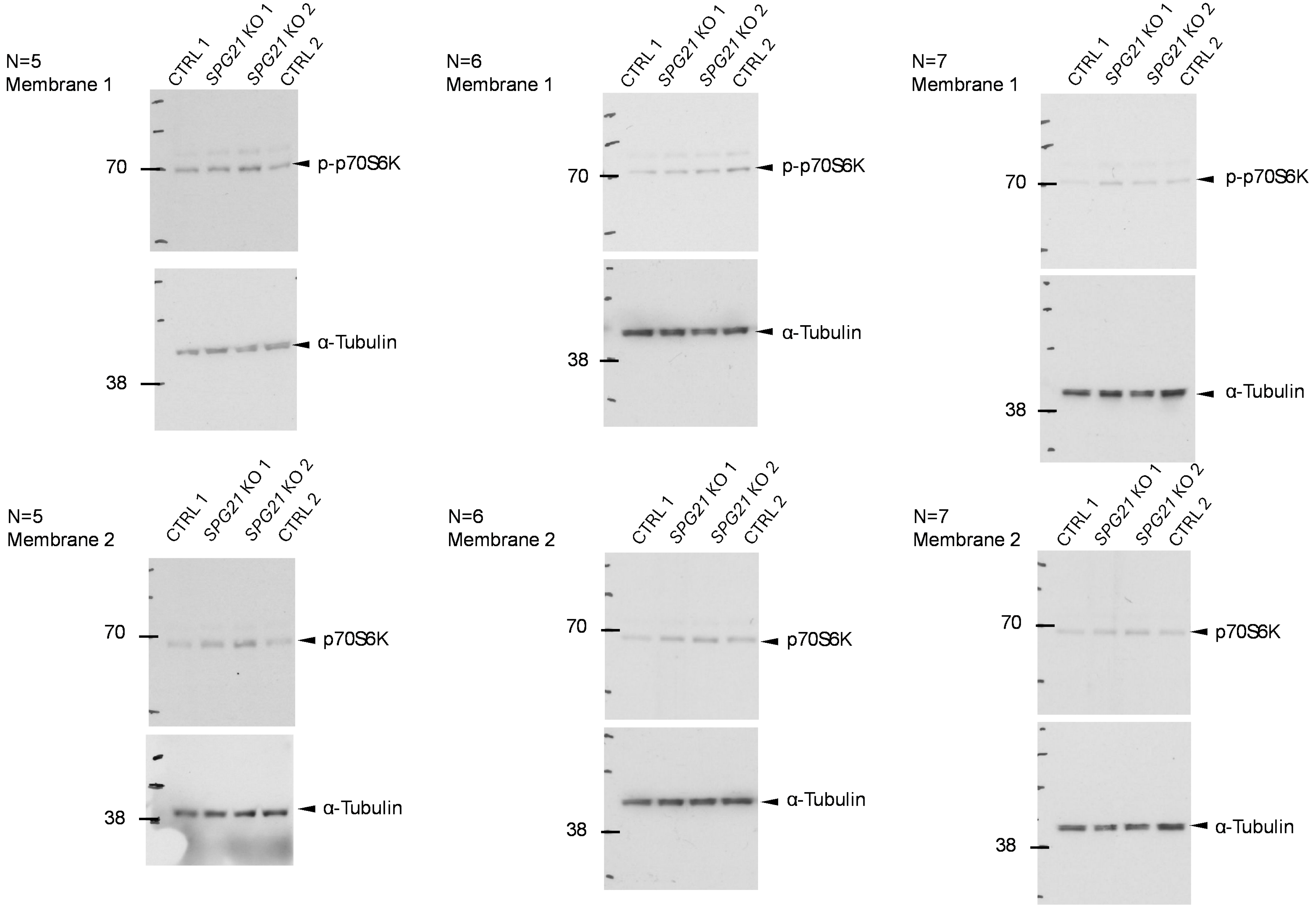
N=5
Membrane 1
CTRL 1
SPG21 KO 1
SPG21 KO 2
CTRL 2
70
p-p70S6K
α-Tubulin
38
N=6
Membrane 1
CTRL 1
SPG21 KO 1
SPG21 KO 2
CTRL 2
70
p-p70S6K
α-Tubulin
38
N=7
Membrane 1
CTRL 1
SPG21 KO 1
SPG21 KO 2
CTRL 2
70
p-p70S6K
α-Tubulin
38
N=5
Membrane 2
CTRL 1
SPG21 KO 1
SPG21 KO 2
CTRL 2
70
p70S6K
α-Tubulin
38
N=6
Membrane 2
CTRL 1
SPG21 KO 1
SPG21 KO 2
CTRL 2
70
p70S6K
α-Tubulin
38
N=7
Membrane 2
CTRL 1
SPG21 KO 1
SPG21 KO 2
CTRL 2
70
p70S6K
α-Tubulin
38

Figure 2C

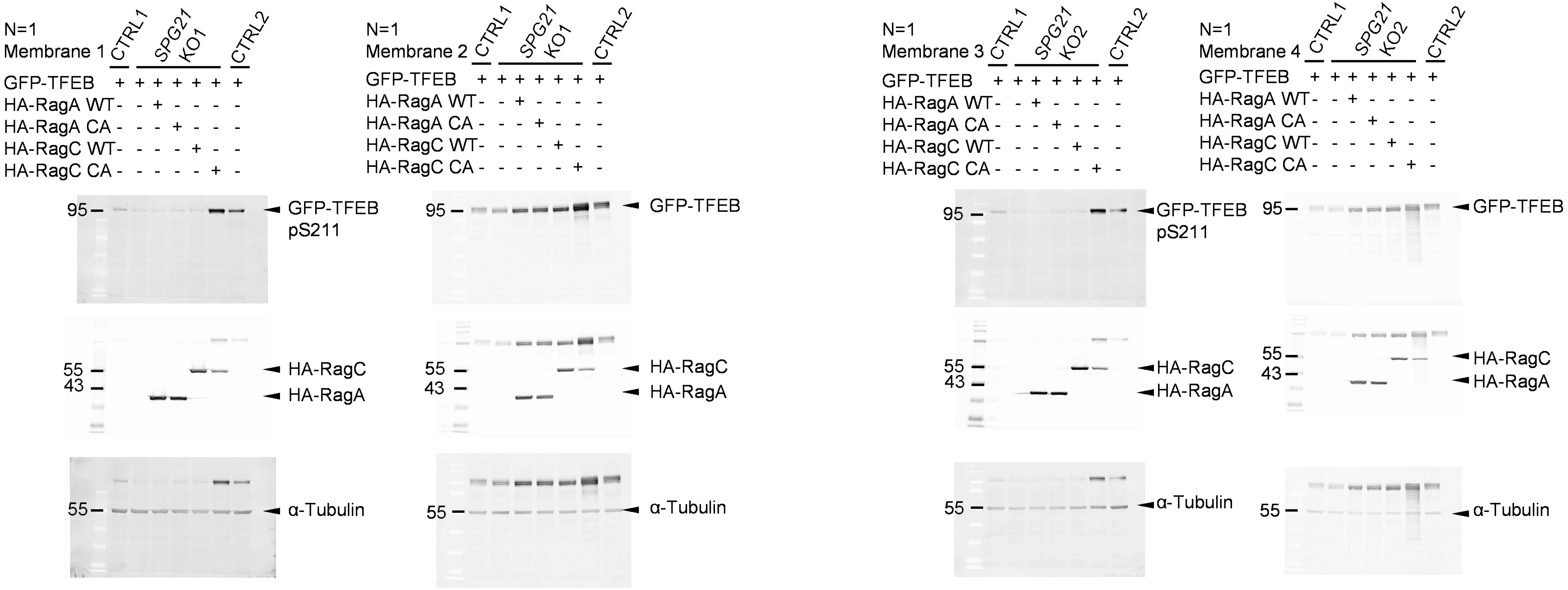

N=1
Membrane 1
CTRL1
SPG21 KO1
CTRL2
GFP-TFEB
HA-RagA WT
HA-RagA CA
HA-RagC WT
HA-RagC CA
95
GFP-TFEB pS211
55
43
HA-RagC
HA-RagA
55
α-Tubulin
N=1
Membrane 2
CTRL1
SPG21 KO1
CTRL2
GFP-TFEB
HA-RagA WT
HA-RagA CA
HA-RagC WT
HA-RagC CA
95
GFP-TFEB
55
43
HA-RagC
HA-RagA
55
α-Tubulin
N=1
Membrane 3
CTRL1
SPG21 KO2
CTRL2
GFP-TFEB
HA-RagA WT
HA-RagA CA
HA-RagC WT
HA-RagC CA
95
GFP-TFEB pS211
55
43
HA-RagC
HA-RagA
55
α-Tubulin
N=1
Membrane 4
CTRL1
SPG21 KO2
CTRL2
GFP-TFEB
HA-RagA WT
HA-RagA CA
HA-RagC WT
HA-RagC CA
95
GFP-TFEB
55
43
HA-RagC
HA-RagA
55
α-Tubulin

Figure 2C

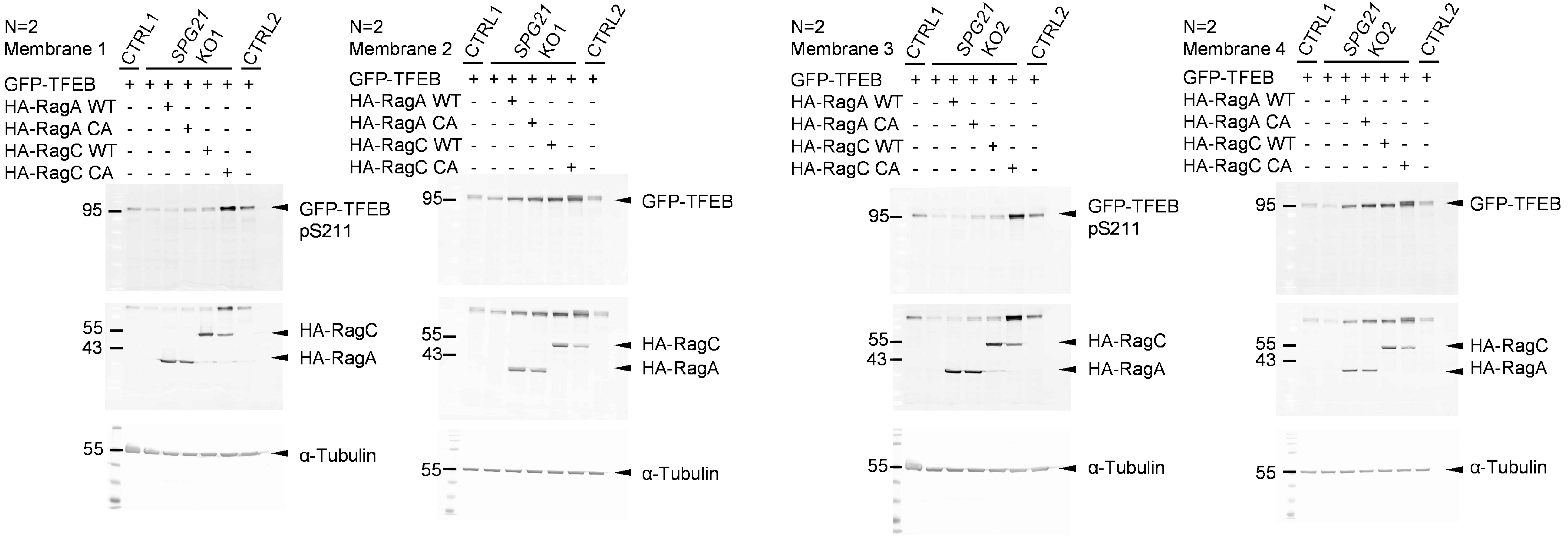
N=2
Membrane 1
CTRL1
SPG21 KO1
CTRL2
GFP-TFEB + + + + + + +
HA-RagA WT - - + - - - -
HA-RagA CA - - - + - - -
HA-RagC WT - - - - + - -
HA-RagC CA - - - - - + -
95
GFP-TFEB pS211
55
43
HA-RagC
HA-RagA
55
α-Tubulin
N=2
Membrane 2
CTRL1
SPG21 KO1
CTRL2
GFP-TFEB + + + + + + +
HA-RagA WT - - + - - - -
HA-RagA CA - - - + - - -
HA-RagC WT - - - - + - -
HA-RagC CA - - - - - + -
95
GFP-TFEB
55
43
HA-RagC
HA-RagA
55
α-Tubulin
N=2
Membrane 3
CTRL1
SPG21 KO2
CTRL2
GFP-TFEB + + + + + + +
HA-RagA WT - - + - - - -
HA-RagA CA - - - + - - -
HA-RagC WT - - - - + - -
HA-RagC CA - - - - - + -
95
GFP-TFEB pS211
55
43
HA-RagC
HA-RagA
55
α-Tubulin
N=2
Membrane 4
CTRL1
SPG21 KO2
CTRL2
GFP-TFEB + + + + + + +
HA-RagA WT - - + - - - -
HA-RagA CA - - - + - - -
HA-RagC WT - - - - + - -
HA-RagC CA - - - - - + -
95
GFP-TFEB
55
43
HA-RagC
HA-RagA
55
α-Tubulin

Figure 2C

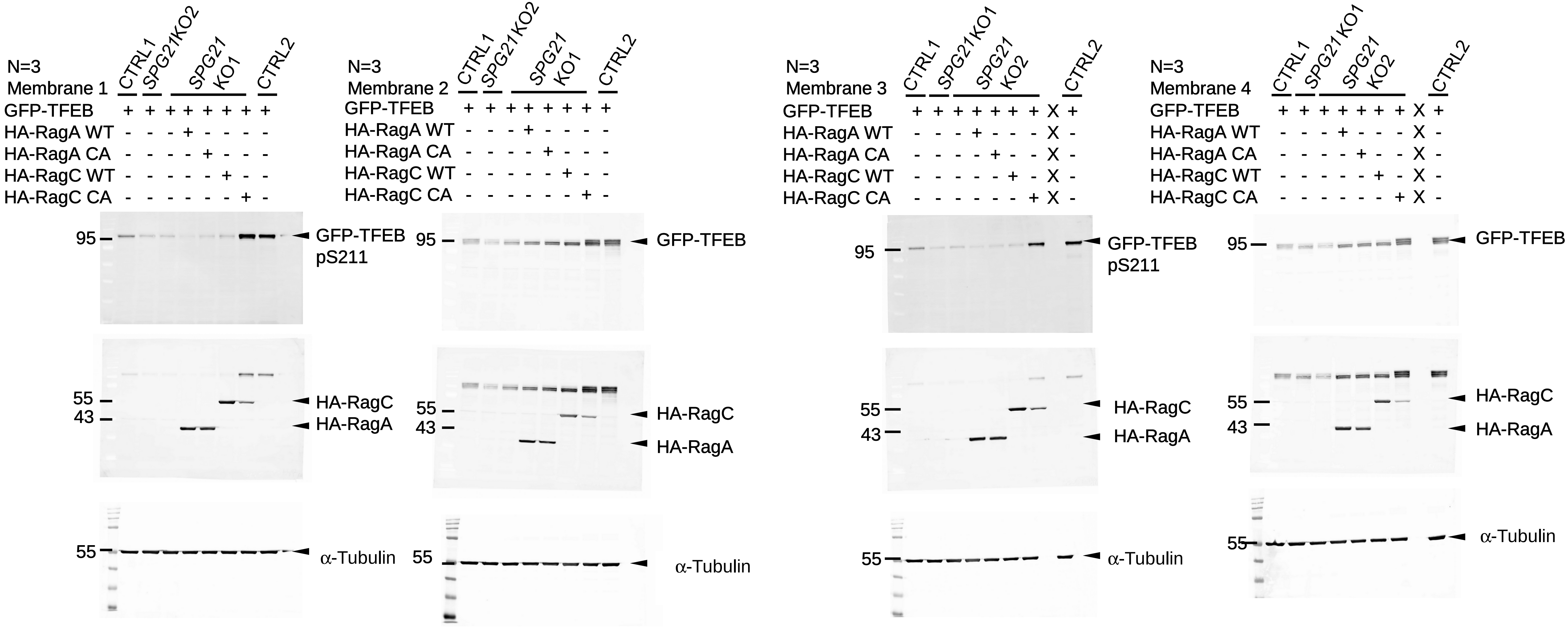
N=3
Membrane 1
CTRL1
SPG21KO2
SPG21 KO1
CTRL2
GFP-TFEB + + + + + + + +
HA-RagA WT - - - + - - - -
HA-RagA CA - - - - + - - -
HA-RagC WT - - - - - + - -
HA-RagC CA - - - - - - + -
95
GFP-TFEB pS211
55
43
HA-RagC
HA-RagA
55
α-Tubulin
N=3
Membrane 2
CTRL1
SPG21KO2
SPG21 KO1
CTRL2
GFP-TFEB + + + + + + + +
HA-RagA WT - - - + - - - -
HA-RagA CA - - - - + - - -
HA-RagC WT - - - - - + - -
HA-RagC CA - - - - - - + -
95
GFP-TFEB
55
43
HA-RagC
HA-RagA
55
α-Tubulin
N=3
Membrane 3
CTRL1
SPG21KO1
SPG21 KO2
CTRL2
GFP-TFEB + + + + + + + X +
HA-RagA WT - - - + - - - X -
HA-RagA CA - - - - + - - X -
HA-RagC WT - - - - - + - X -
HA-RagC CA - - - - - - + X -
95
GFP-TFEB pS211
55
43
HA-RagC
HA-RagA
55
α-Tubulin
N=3
Membrane 4
CTRL1
SPG21KO1
SPG21 KO2
CTRL2
GFP-TFEB + + + + + + + X +
HA-RagA WT - - - + - - - X -
HA-RagA CA - - - - + - - X -
HA-RagC WT - - - - - + - X -
HA-RagC CA - - - - - - + X -
95
GFP-TFEB
55
43
HA-RagC
HA-RagA
55
α-Tubulin

Figure 2C

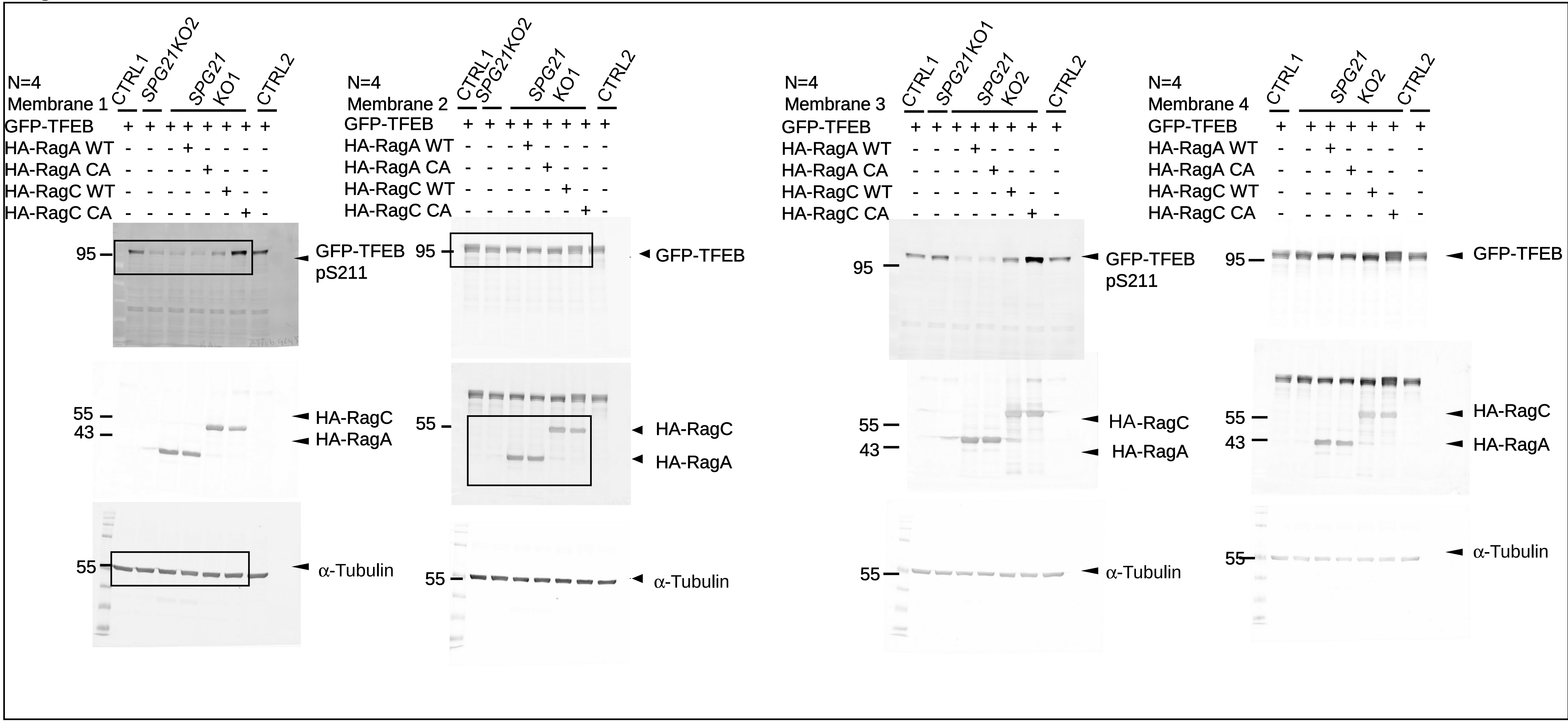

Data shown in the article extracted from this set

Figure 2E N=1

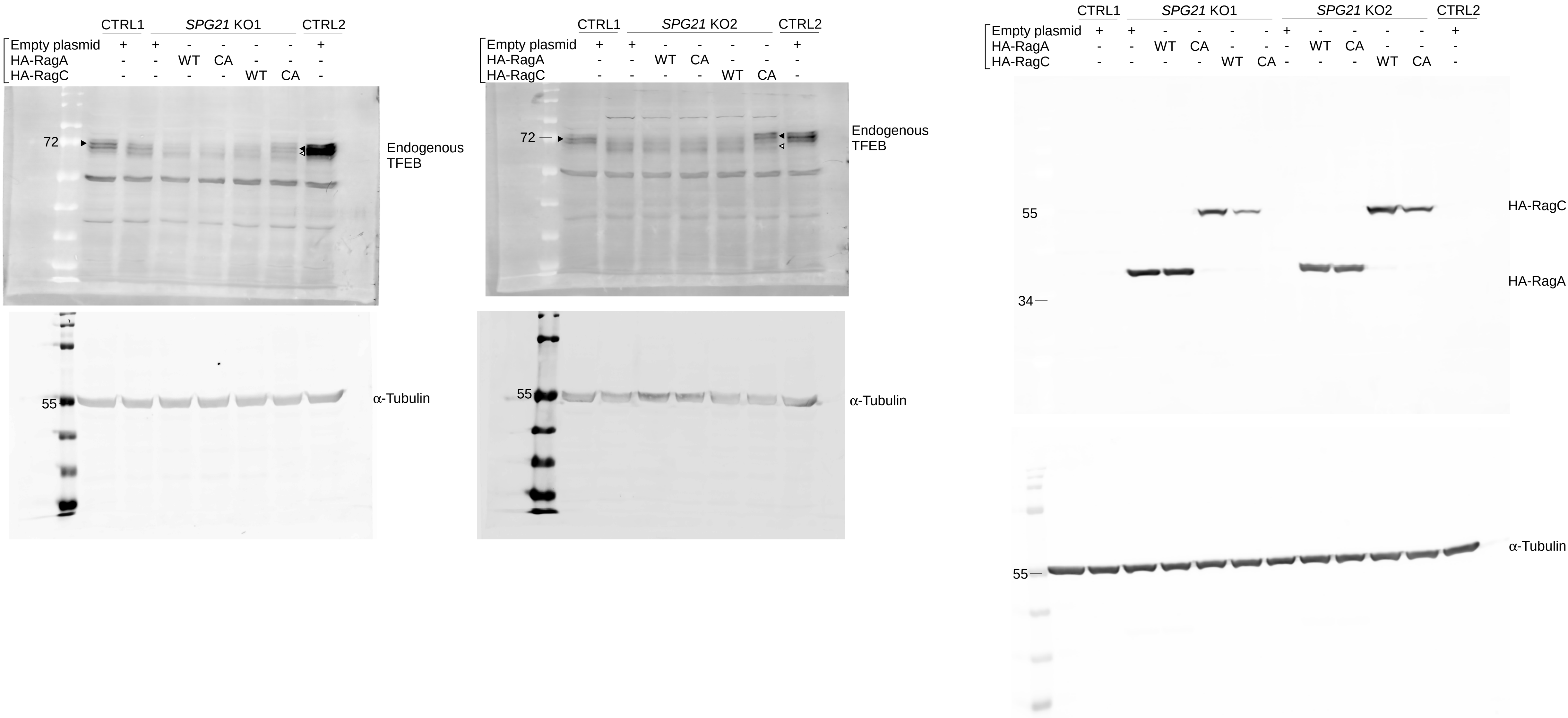
CTRL1
SPG21 KO1
CTRL2
Empty plasmid + + - - - - +
HA-RagA - - WT CA - - -
HA-RagC - - - - WT CA -
72
Endogenous TFEB
55
α-Tubulin
CTRL1
SPG21 KO2
CTRL2
Empty plasmid + + - - - - +
HA-RagA - - WT CA - - -
HA-RagC - - - - WT CA -
72
Endogenous TFEB
55
α-Tubulin
CTRL1
SPG21 KO1
SPG21 KO2
CTRL2
Empty plasmid + + - - - - + - - - - +
HA-RagA - - WT CA - - - WT CA - - -
HA-RagC - - - - WT CA - - - WT CA -
55
34
HA-RagC
HA-RagA
55
α-Tubulin

Figure 2E N=2

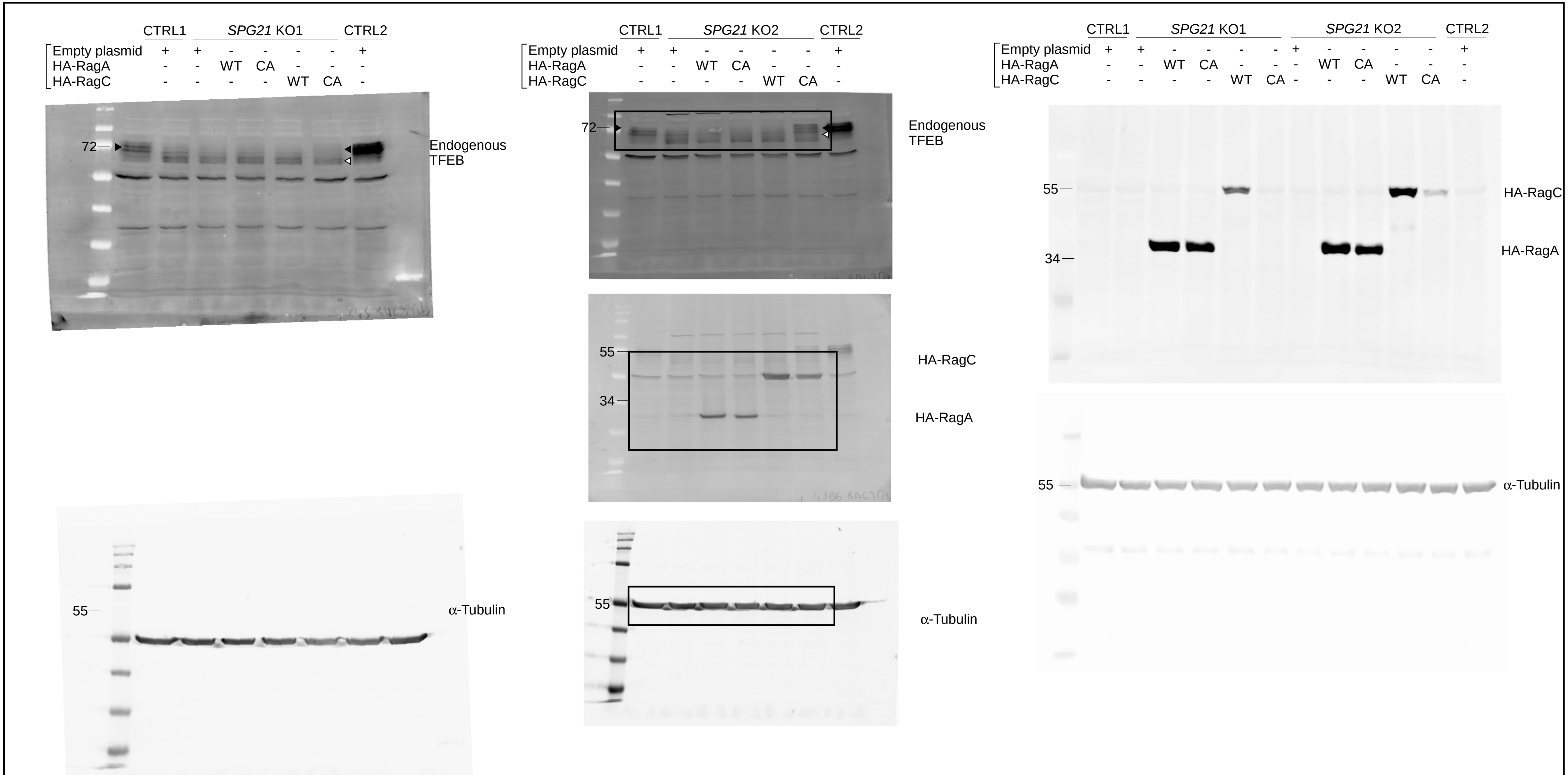

Data shown in the article extracted from this set

Figure 2E N=3

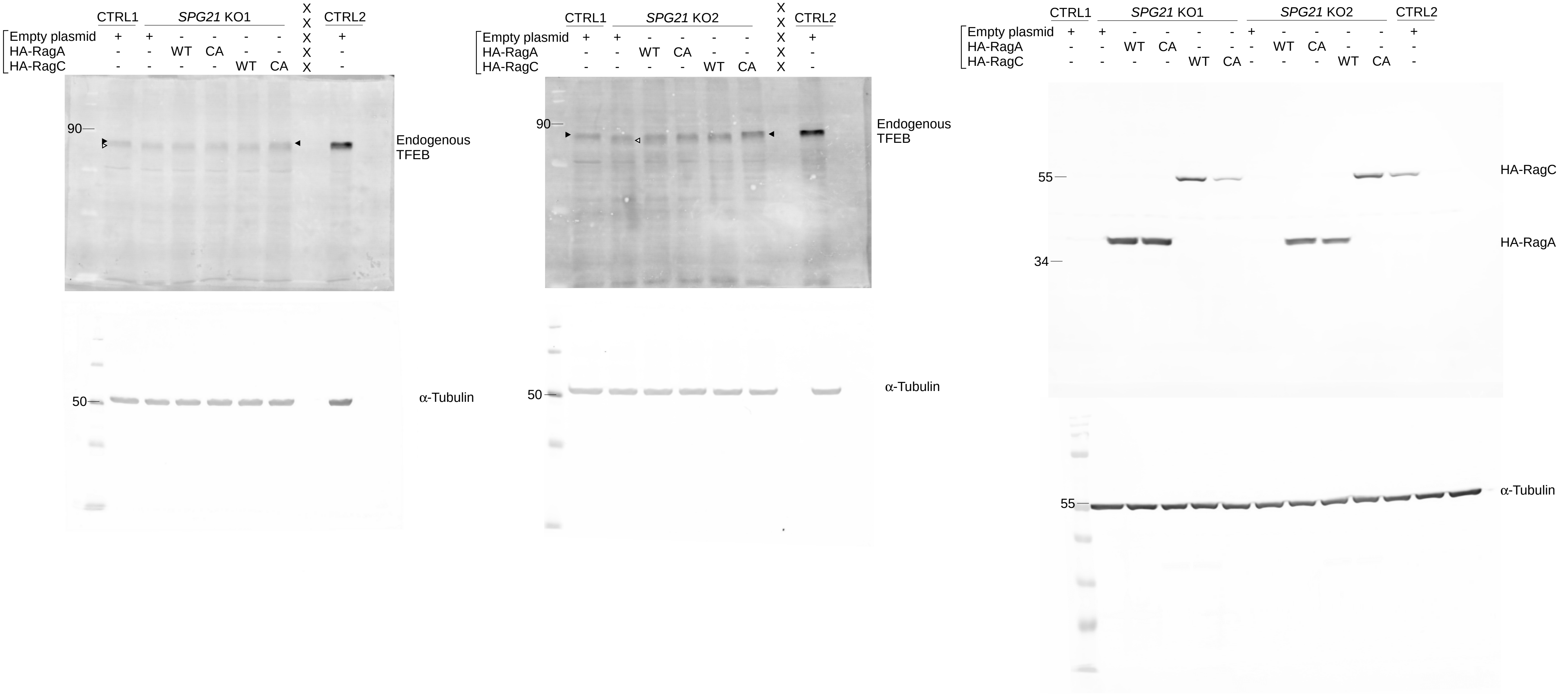
CTRL1
SPG21 KO1
CTRL2
Empty plasmid
HA-RagA
HA-RagC
WT
CA
90
50
Endogenous TFEB
α-Tubulin
CTRL1
SPG21 KO2
CTRL2
90
50
Endogenous TFEB
α-Tubulin
CTRL1
SPG21 KO1
SPG21 KO2
CTRL2
55
34
HA-RagC
HA-RagA
55
α-Tubulin

Figure 2E N=4

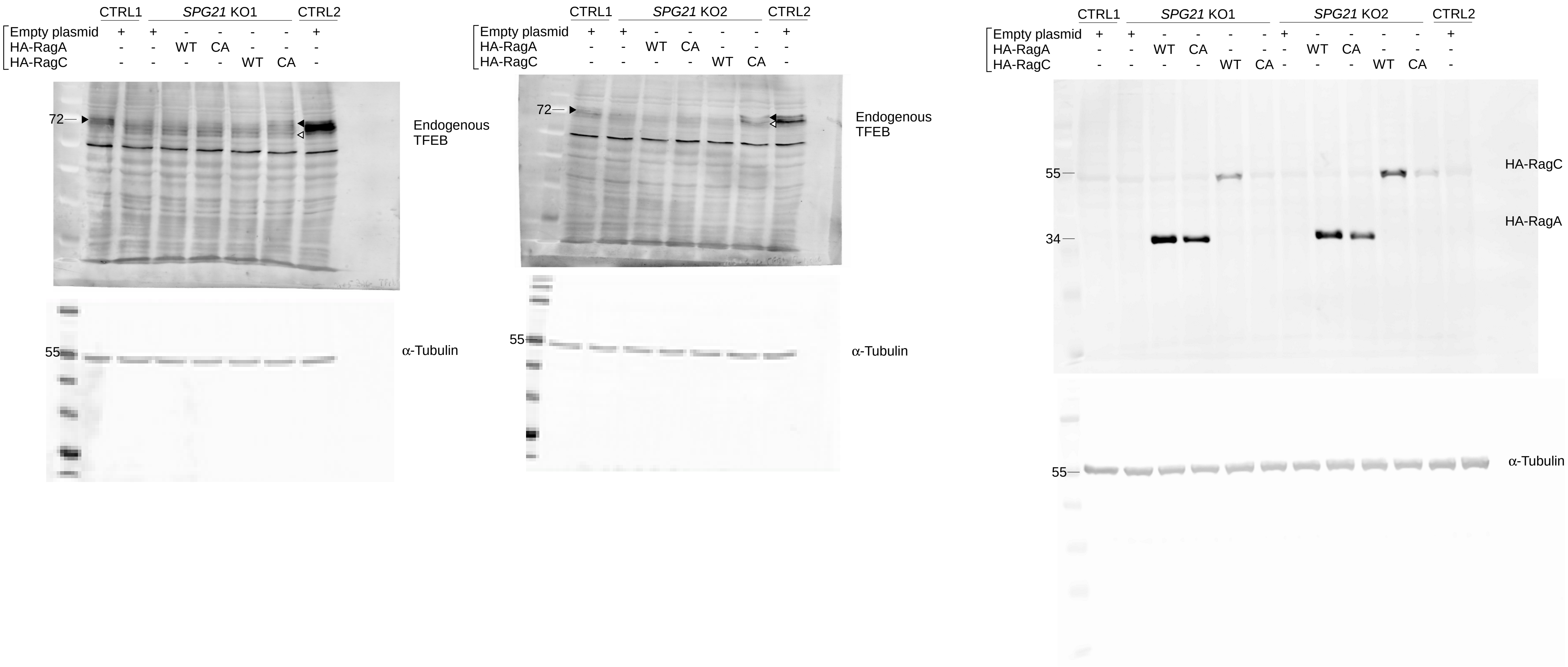
CTRL1
SPG21 KO1
CTRL2
Empty plasmid + + - - - - +
HA-RagA - - WT CA - - -
HA-RagC - - - - WT CA -
72
Endogenous TFEB
55
α-Tubulin
CTRL1
SPG21 KO2
CTRL2
Empty plasmid + + - - - - +
HA-RagA - - WT CA - - -
HA-RagC - - - - WT CA -
72
Endogenous TFEB
55
α-Tubulin
CTRL1
SPG21 KO1
SPG21 KO2
CTRL2
Empty plasmid + + - - - - + - - - - +
HA-RagA - - WT CA - - - WT CA - - -
HA-RagC - - - - WT CA - - - WT CA -
55
34
HA-RagC
HA-RagA
55
α-Tubulin
