# Supplementary material for: Maspardin/SPG21 controls lysosome motility and TFEB phosphorylation through RAB7 positioning
Source: J Cell Biol. 2025 Dec 16;225(2):e202501135. doi: 10.1083/jcb.202501135 (PMC12707310; doi:10.1083/jcb.202501135)
Supplement: SourceData F3 — is the source file for Fig. 3. [file jcb_202501135_sourcedataf3.pdf]

Figure 3C

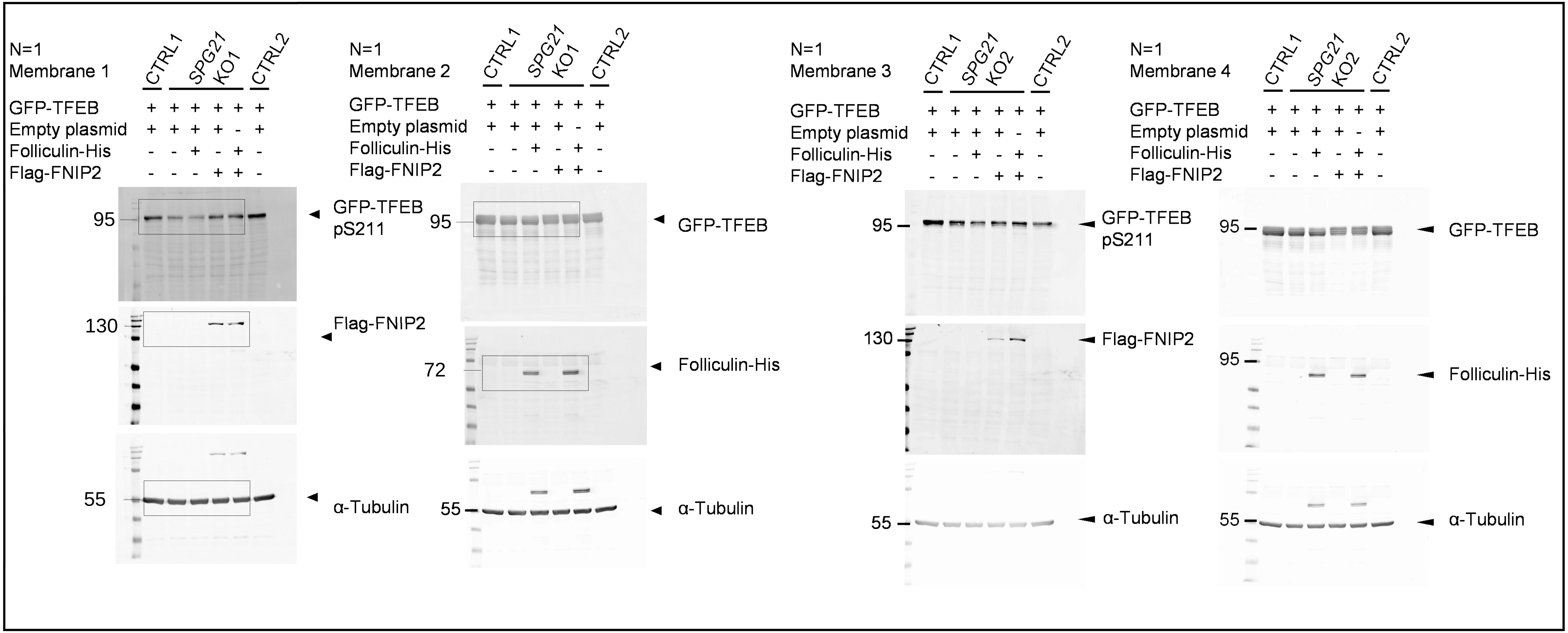

Data shown in the article extracted from this set

Figure 3C

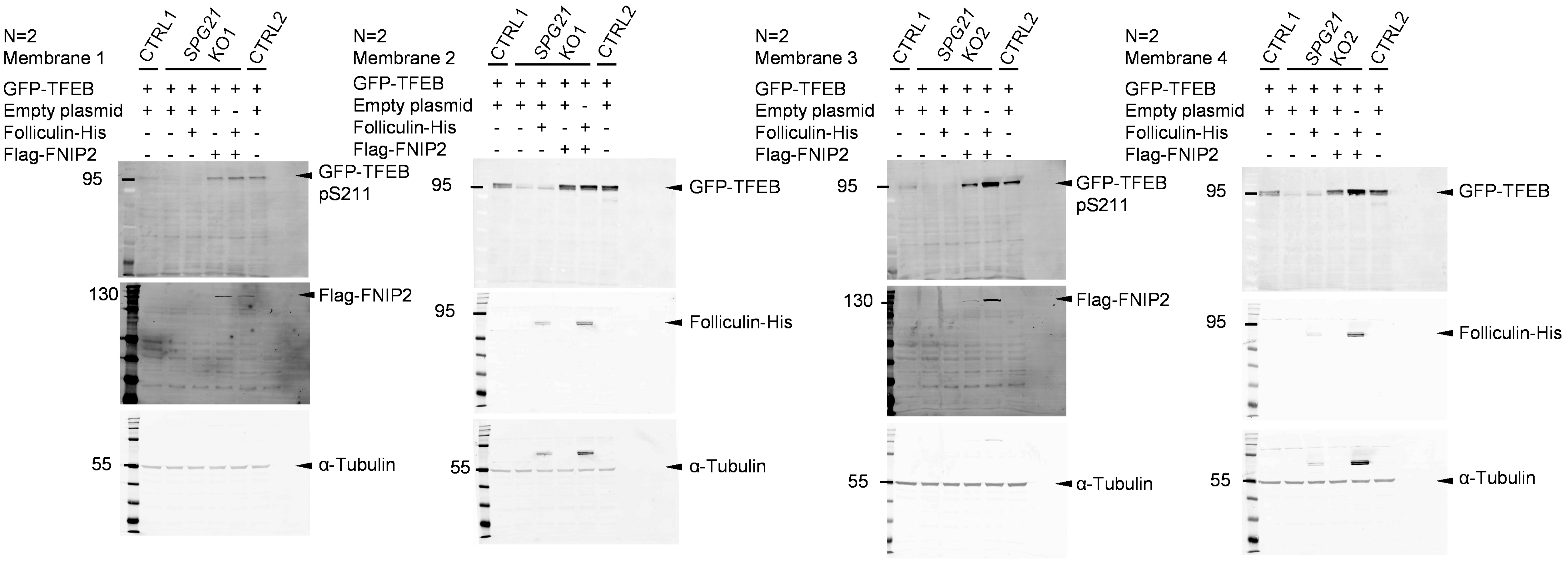
N=2
Membrane 1
CTRL1
SPG21 KO1
CTRL2
GFP-TFEB + + + + + +
Empty plasmid + + + + - +
Folliculin-His - - + - + -
Flag-FNIP2 - - - + + -
95
GFP-TFEB pS211
130
Flag-FNIP2
55
α-Tubulin
N=2
Membrane 2
CTRL1
SPG21 KO1
CTRL2
GFP-TFEB + + + + + +
Empty plasmid + + + + - +
Folliculin-His - - + - + -
Flag-FNIP2 - - - + + -
95
GFP-TFEB
95
Folliculin-His
55
α-Tubulin
N=2
Membrane 3
CTRL1
SPG21 KO2
CTRL2
GFP-TFEB + + + + + +
Empty plasmid + + + + - +
Folliculin-His - - + - + -
Flag-FNIP2 - - - + + -
95
GFP-TFEB pS211
130
Flag-FNIP2
55
α-Tubulin
N=2
Membrane 4
CTRL1
SPG21 KO2
CTRL2
GFP-TFEB + + + + + +
Empty plasmid + + + + - +
Folliculin-His - - + - + -
Flag-FNIP2 - - - + + -
95
GFP-TFEB
95
Folliculin-His
55
α-Tubulin

Figure 3C

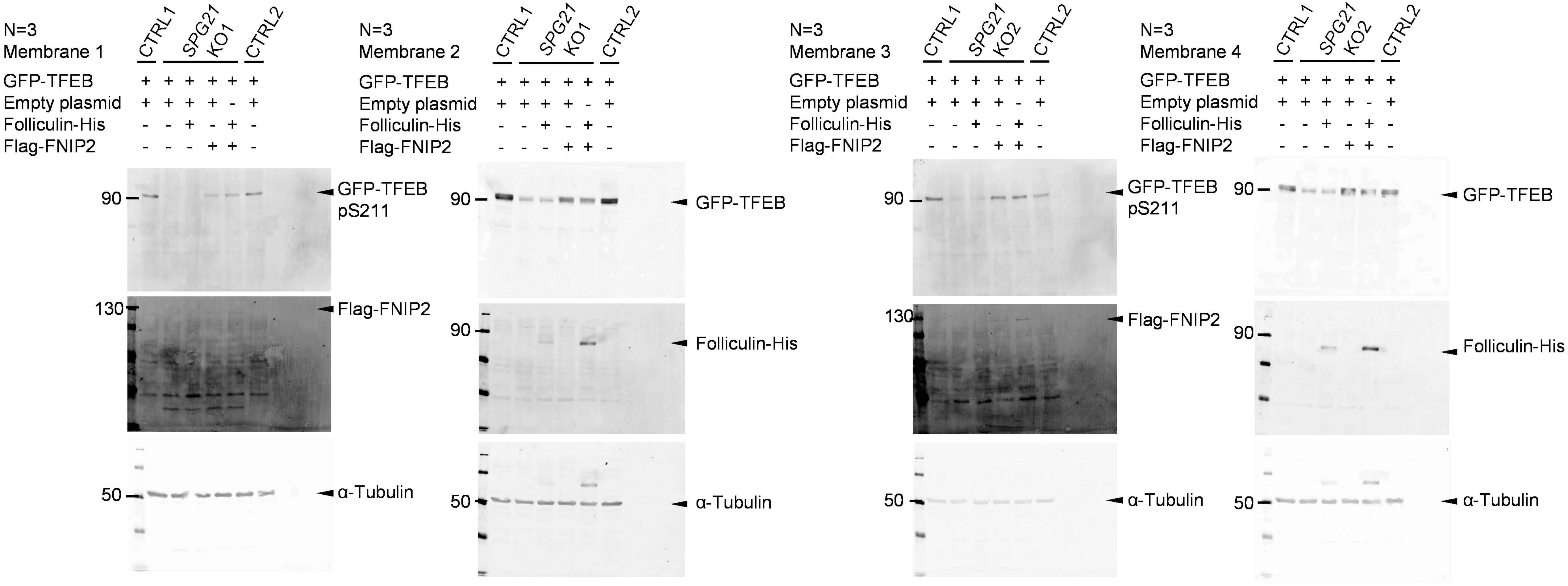
N=3
Membrane 1
CTRL1
SPG21 KO1
CTRL2
GFP-TFEB + + + + + +
Empty plasmid + + + + - +
Folliculin-His - - + - + -
Flag-FNIP2 - - - + + -
90
GFP-TFEB pS211
130
Flag-FNIP2
50
α-Tubulin
N=3
Membrane 2
CTRL1
SPG21 KO1
CTRL2
GFP-TFEB + + + + + +
Empty plasmid + + + + - +
Folliculin-His - - + - + -
Flag-FNIP2 - - - + + -
90
GFP-TFEB
90
Folliculin-His
50
α-Tubulin
N=3
Membrane 3
CTRL1
SPG21 KO2
CTRL2
GFP-TFEB + + + + + +
Empty plasmid + + + + - +
Folliculin-His - - + - + -
Flag-FNIP2 - - - + + -
90
GFP-TFEB pS211
130
Flag-FNIP2
50
α-Tubulin
N=3
Membrane 4
CTRL1
SPG21 KO2
CTRL2
GFP-TFEB + + + + + +
Empty plasmid + + + + - +
Folliculin-His - - + - + -
Flag-FNIP2 - - - + + -
90
GFP-TFEB
90
Folliculin-His
50
α-Tubulin

Figure 3E

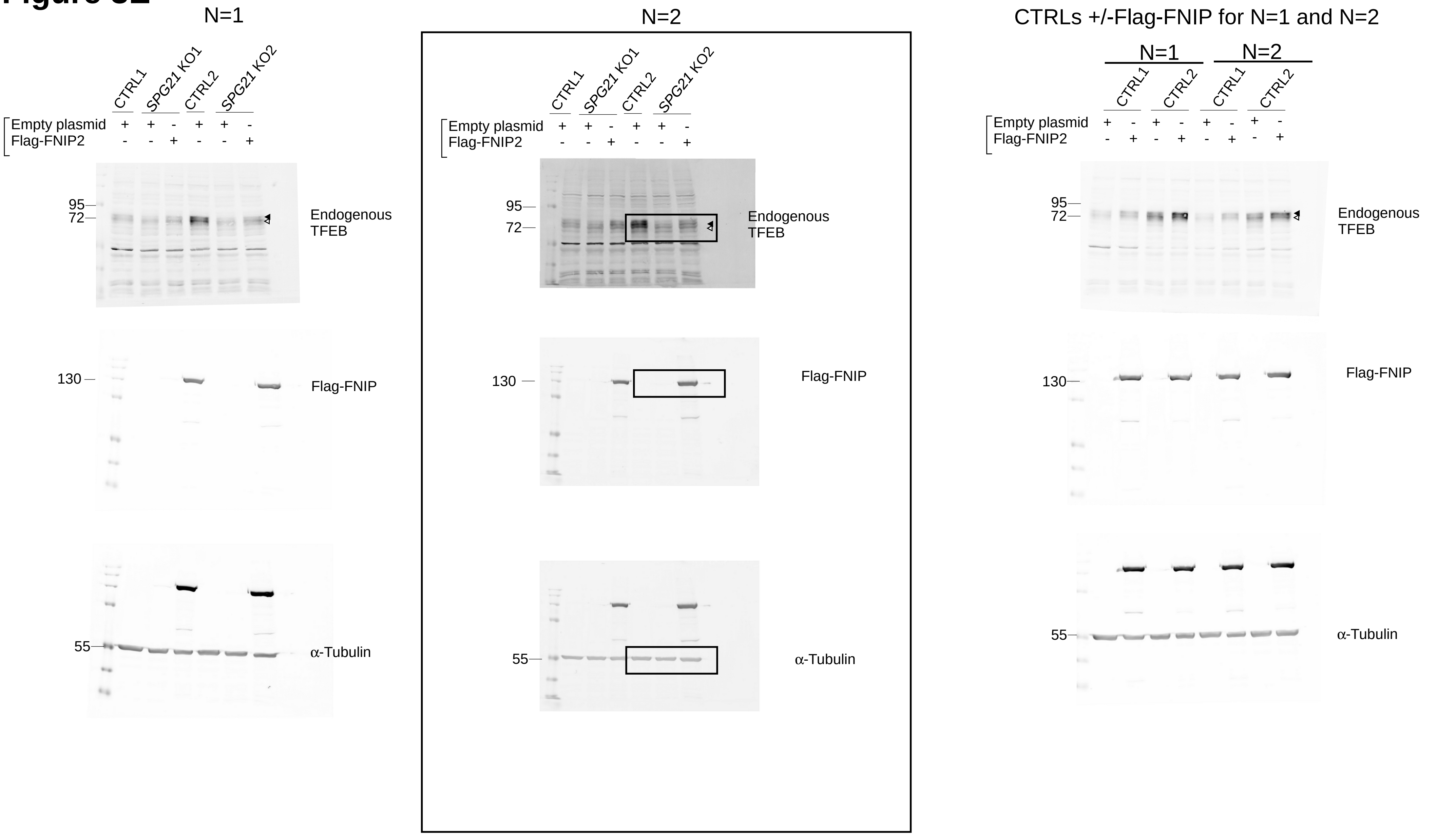

Set shown in Figure 3E

Figure 3E

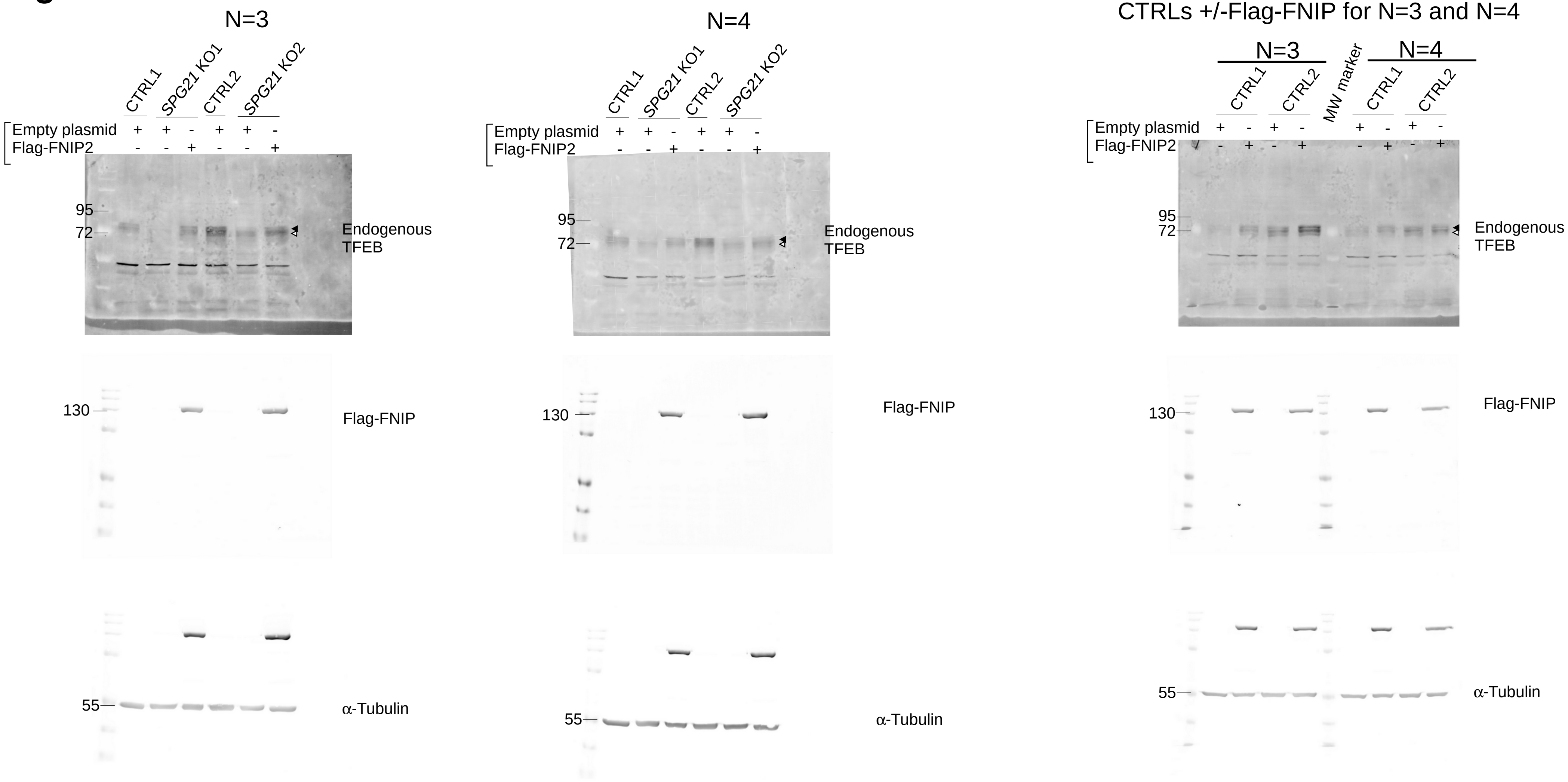

N=3
N=4
CTRLs +/-Flag-FNIP for N=3 and N=4
CTRL1
SPG21 KO1
CTRL2
SPG21 KO2
MW marker
Empty plasmid
Flag-FNIP2
95
72
130
55
Endogenous TFEB
Flag-FNIP
α-Tubulin
