# Supplementary material for: Maspardin/SPG21 controls lysosome motility and TFEB phosphorylation through RAB7 positioning
Source: J Cell Biol. 2025 Dec 16;225(2):e202501135. doi: 10.1083/jcb.202501135 (PMC12707310; doi:10.1083/jcb.202501135)
Supplement: SourceData F4 — is the source file for Fig. 4. [file jcb_202501135_sourcedataf4.pdf]

Figure 4A

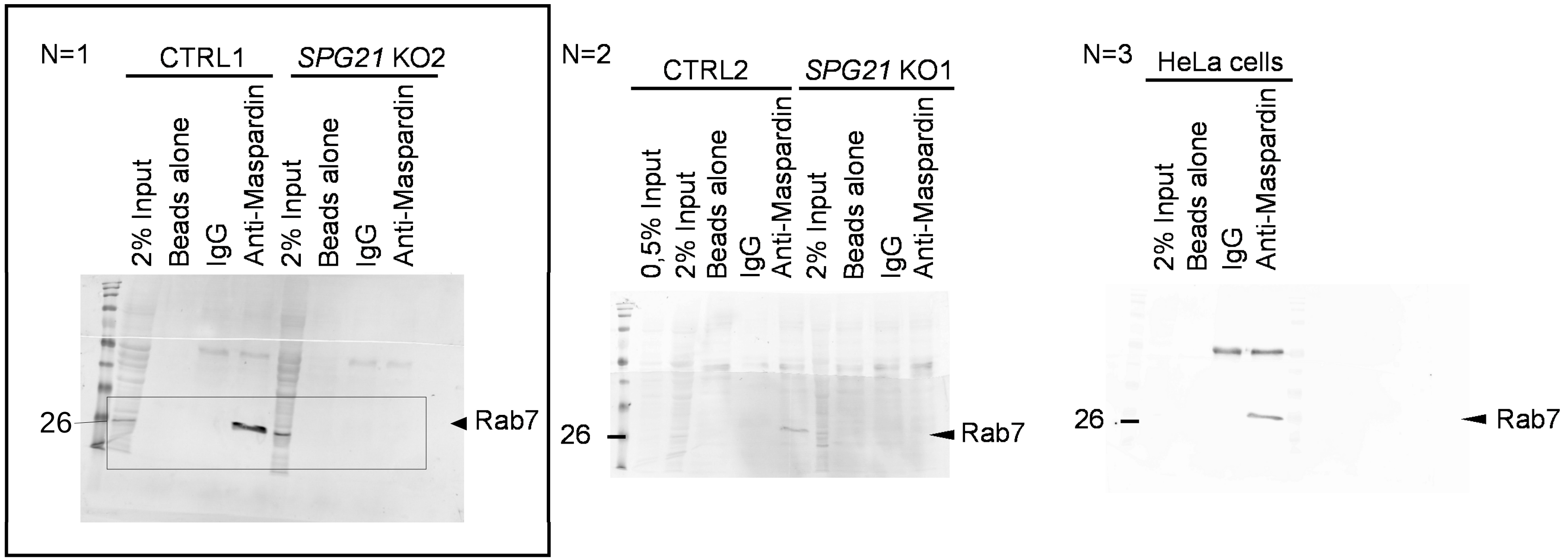

Set shown in the article

Figure 4B

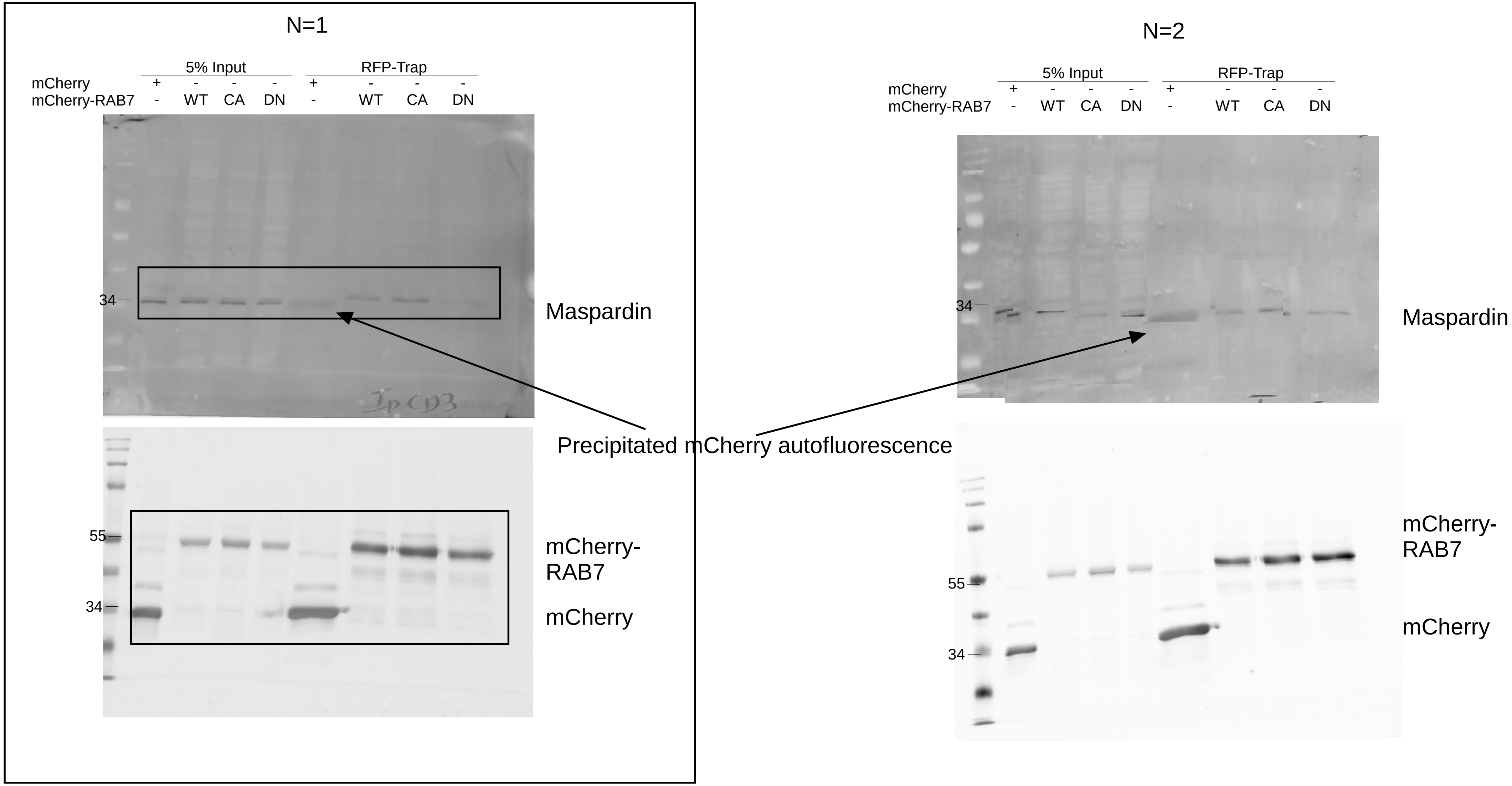

Set shown in the article

Figure 4B

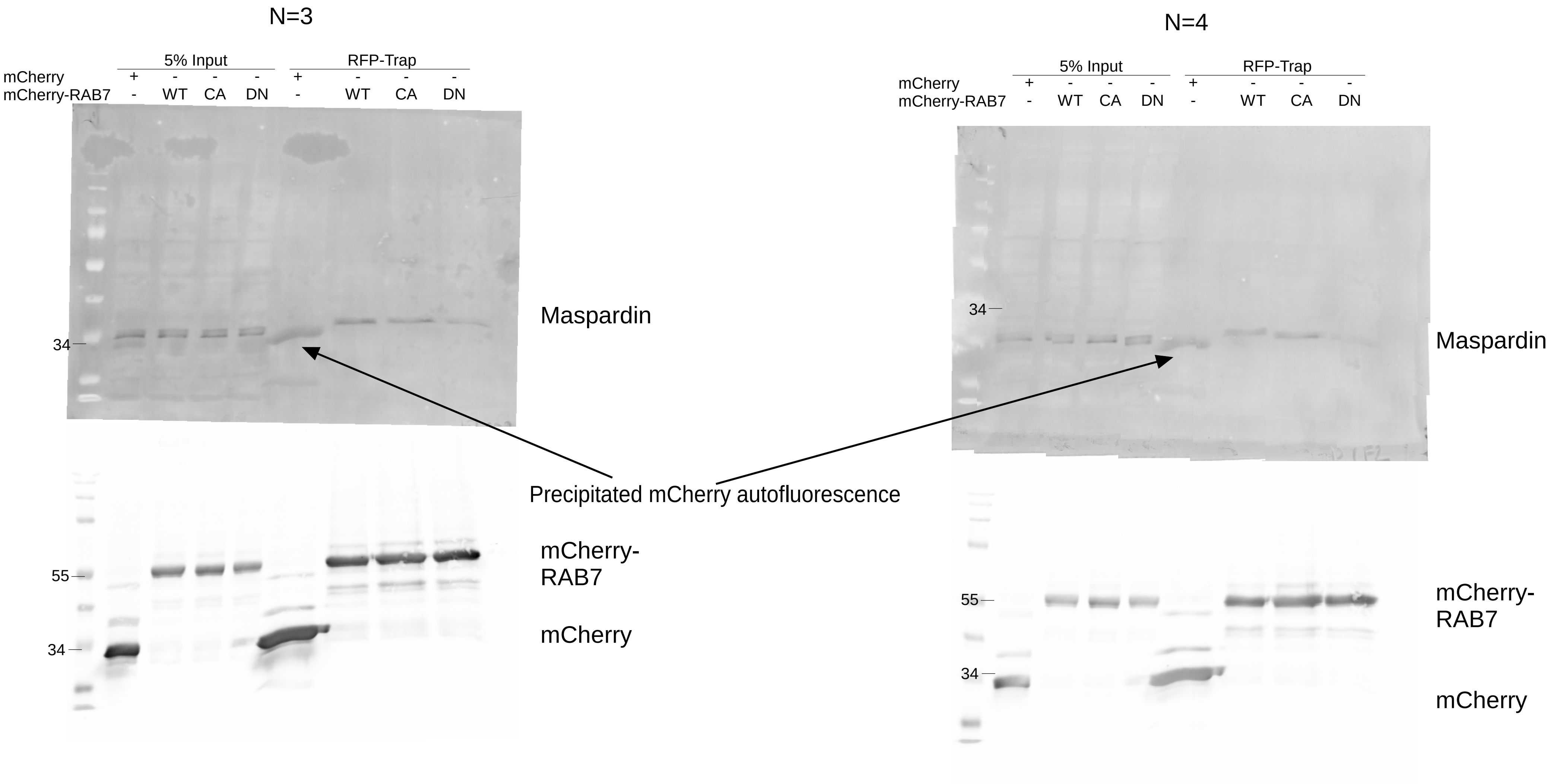

N=3
5% Input
RFP-Trap
mCherry
mCherry-RAB7
WT CA DN WT CA DN
34
55
34
Maspardin
mCherry-RAB7
mCherry
Precipitated mCherry autofluorescence
N=4
5% Input
RFP-Trap
mCherry
mCherry-RAB7
WT CA DN WT CA DN
34
55
34
Maspardin
mCherry-RAB7
mCherry

Figure 4 E

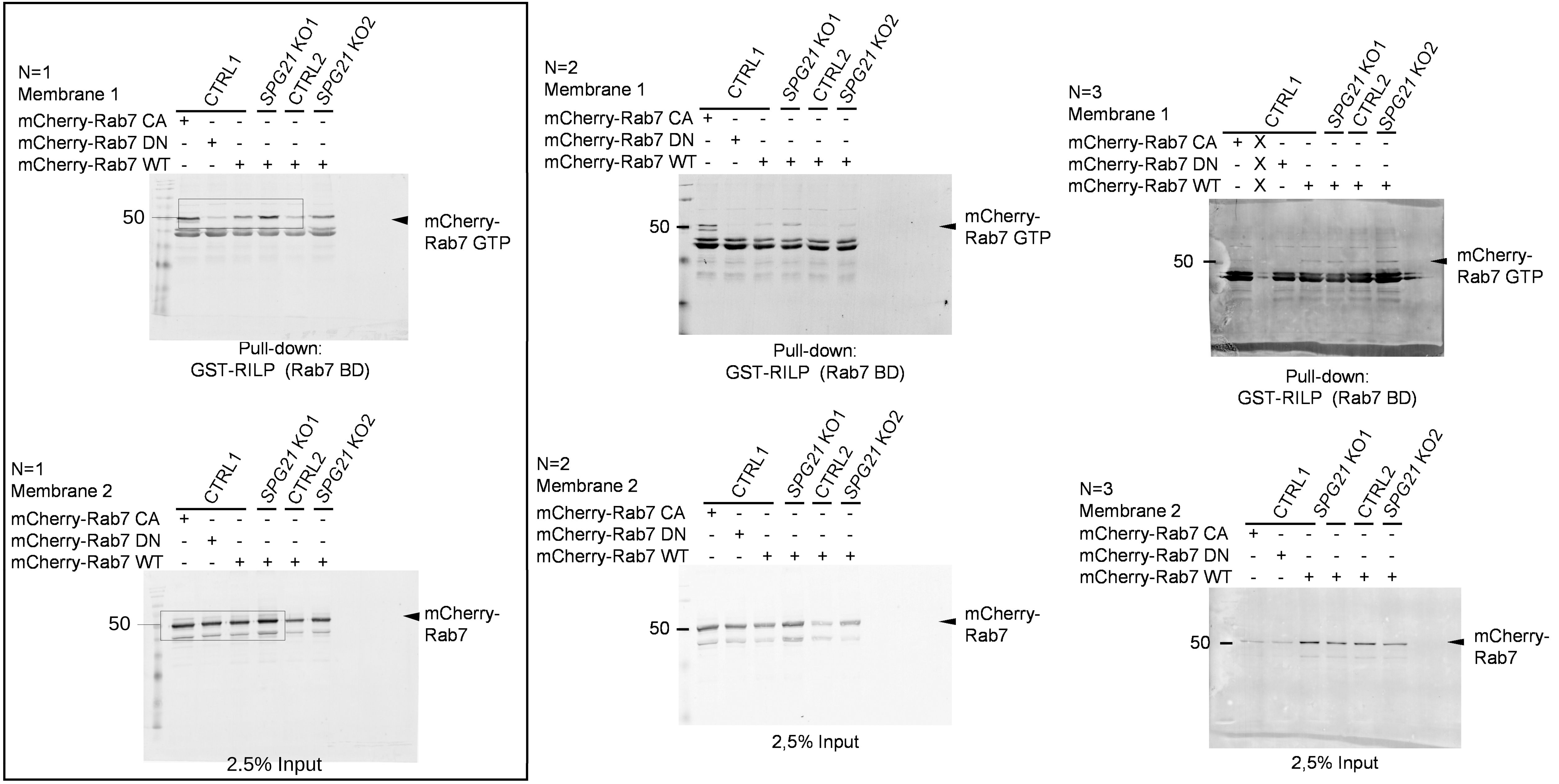

Data shown in the article extracted from this set
