# Supplementary material for: Maspardin/SPG21 controls lysosome motility and TFEB phosphorylation through RAB7 positioning
Source: J Cell Biol. 2025 Dec 16;225(2):e202501135. doi: 10.1083/jcb.202501135 (PMC12707310; doi:10.1083/jcb.202501135)

Figure 5B

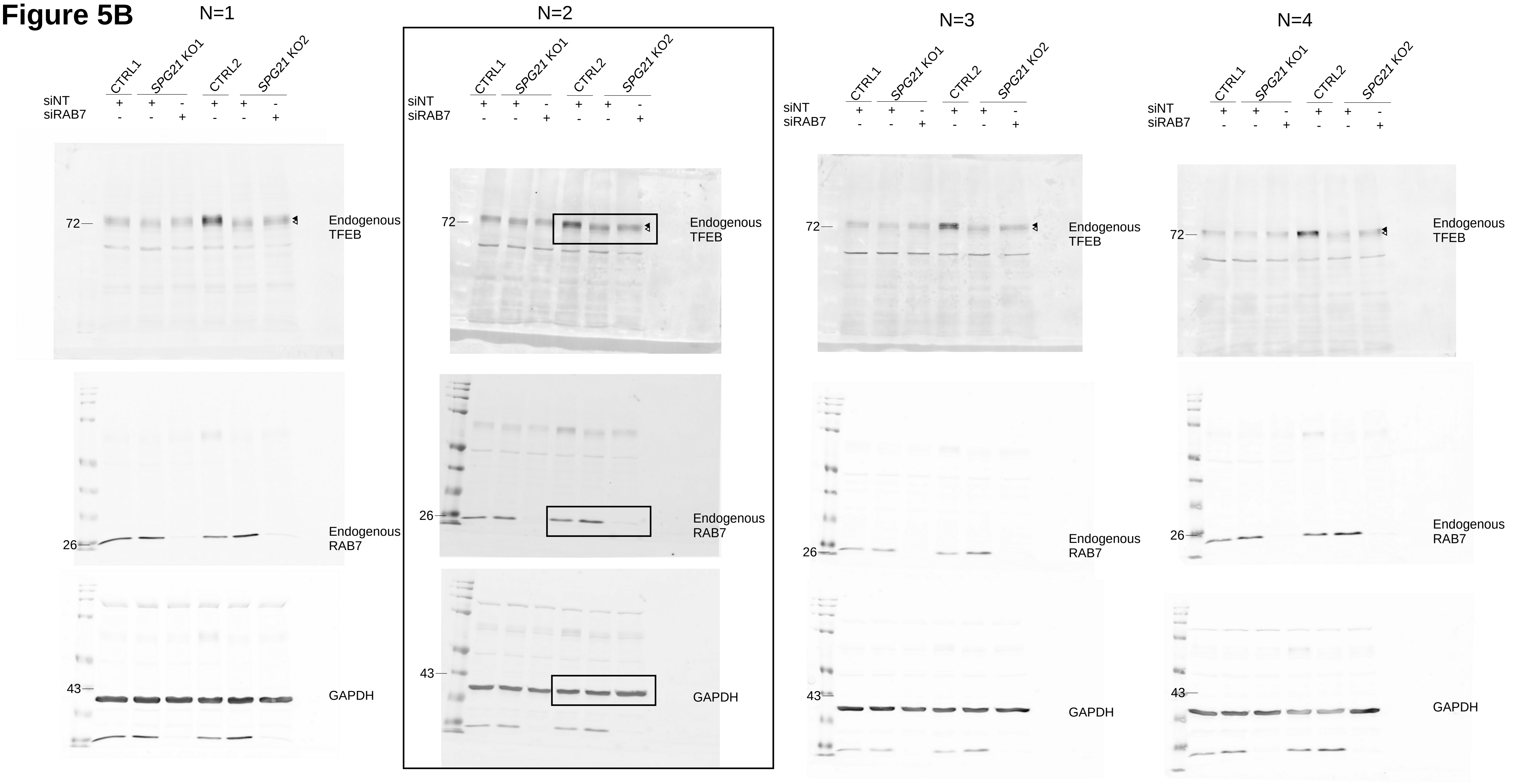

Set shown in the article

Figure 5B
CTL only

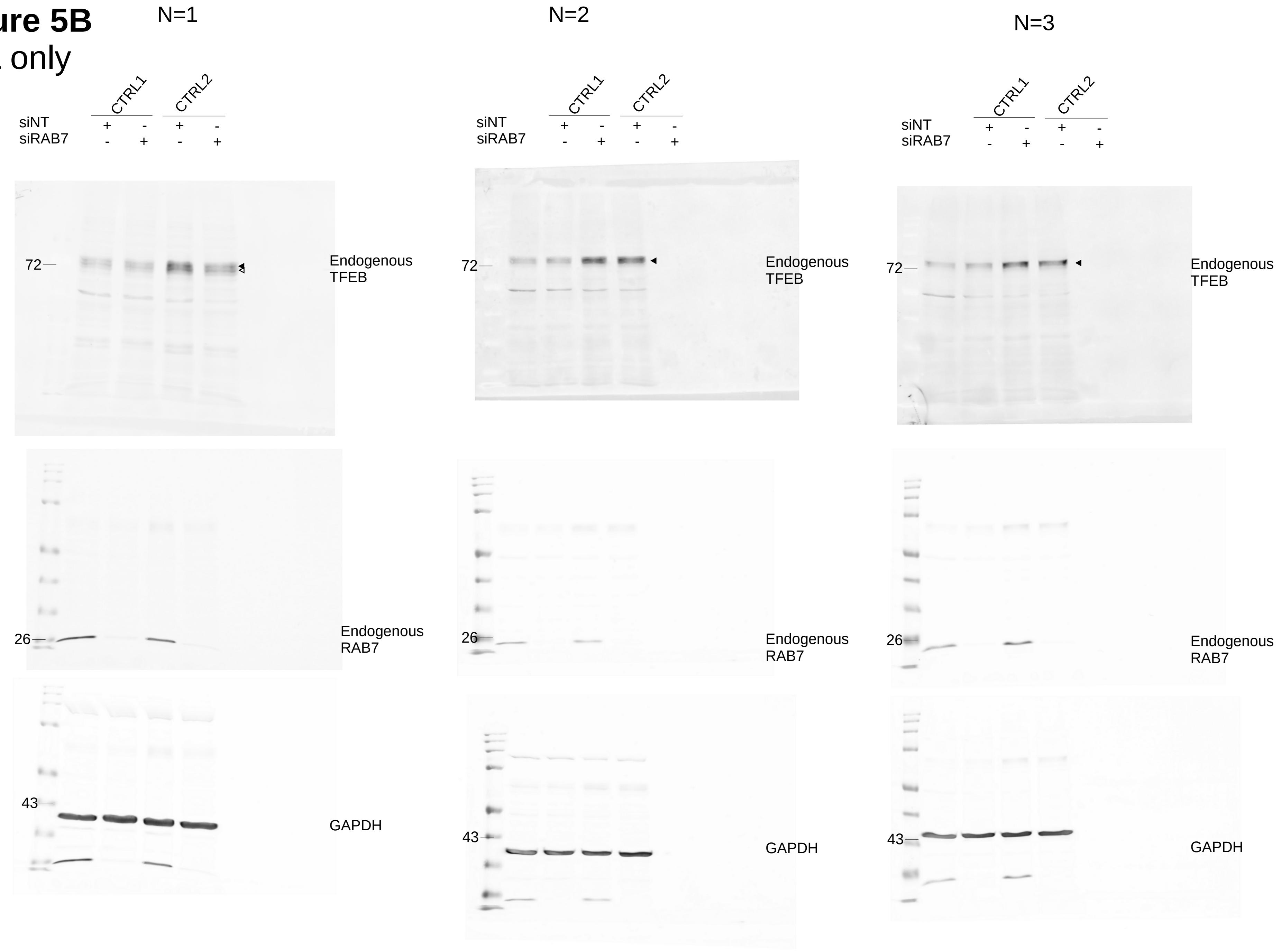

Figure 5C left panel

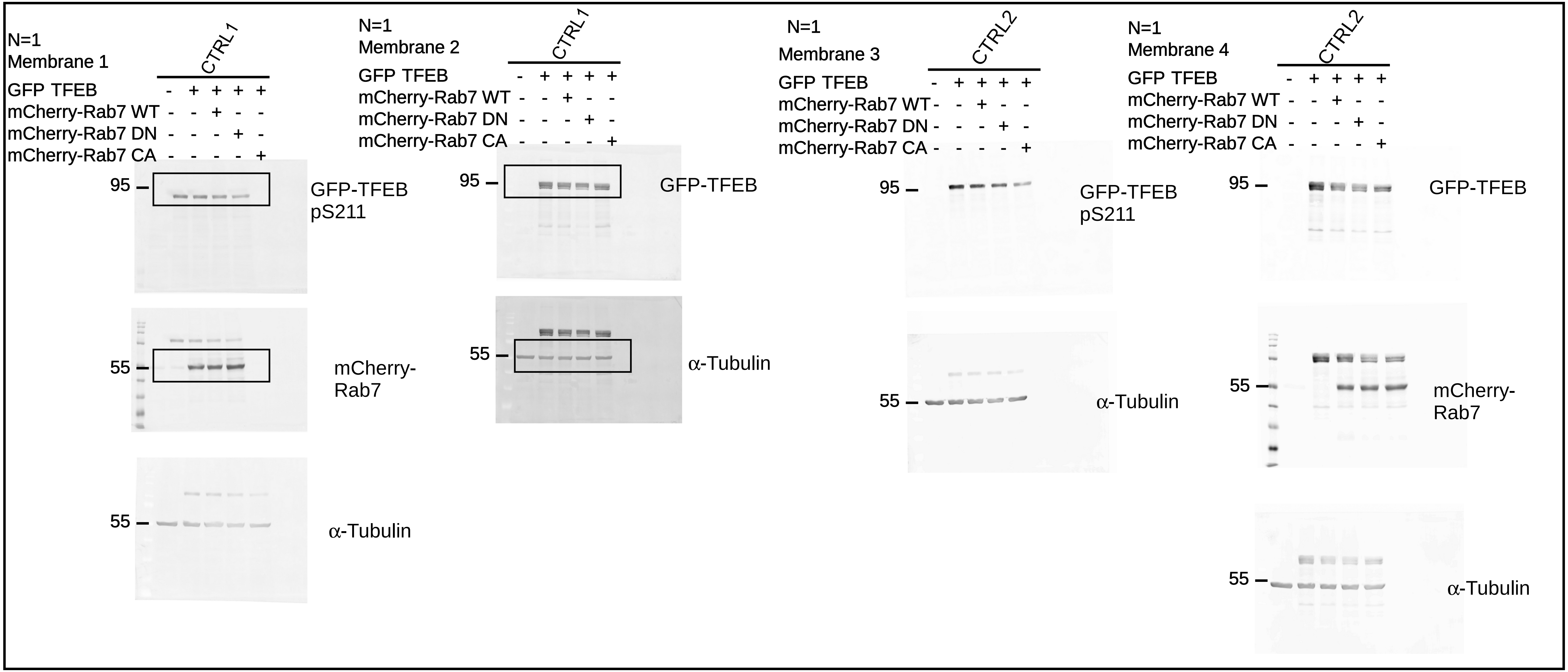

Data shown in the article extracted from this set

Figure 5C left panel

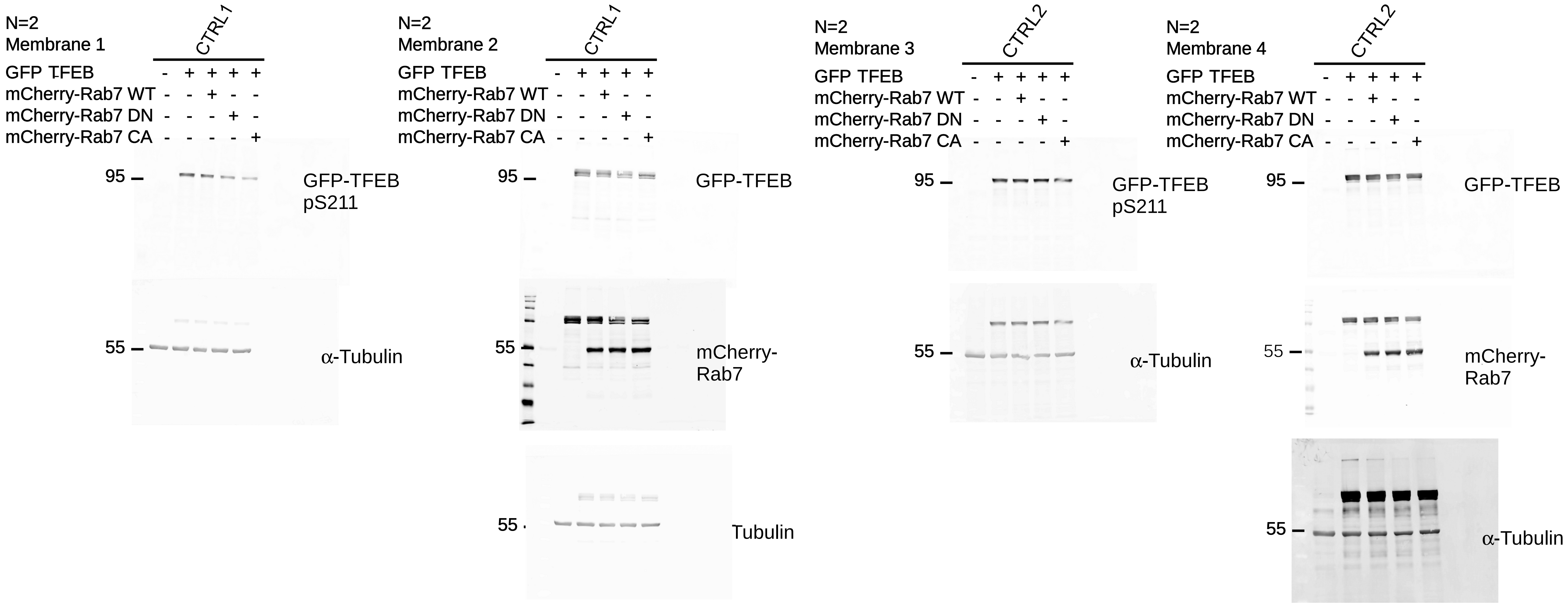

Figure 5C left panel

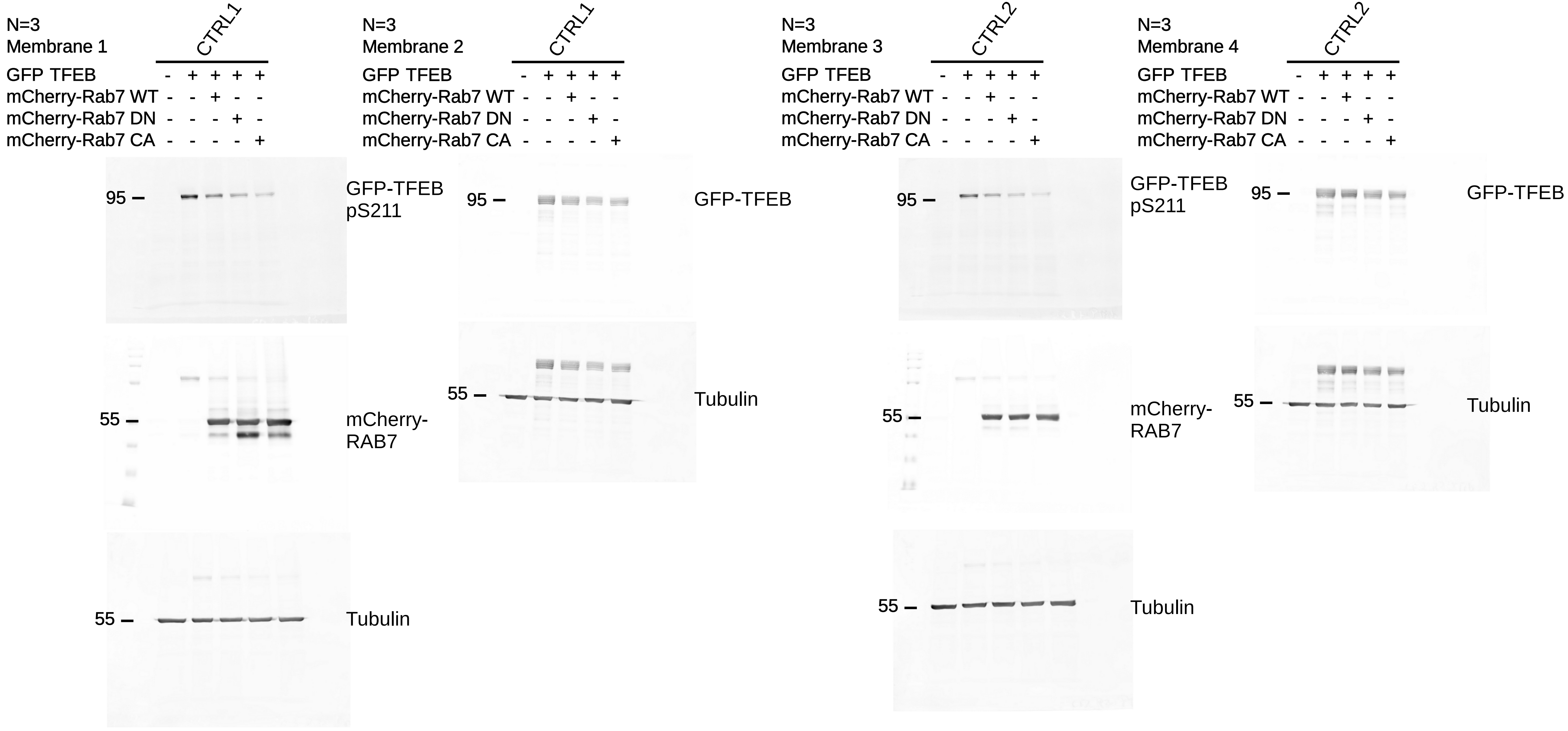

Figure 5C right panel

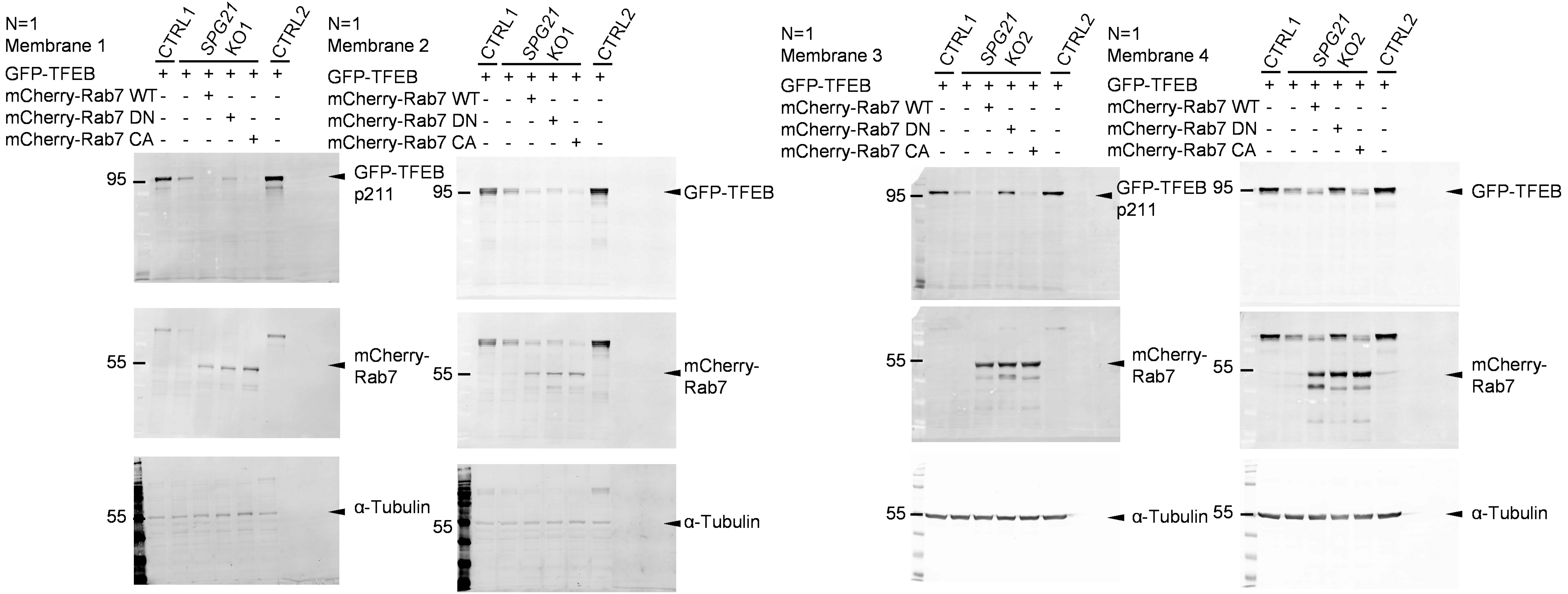

Figure 5C right panel

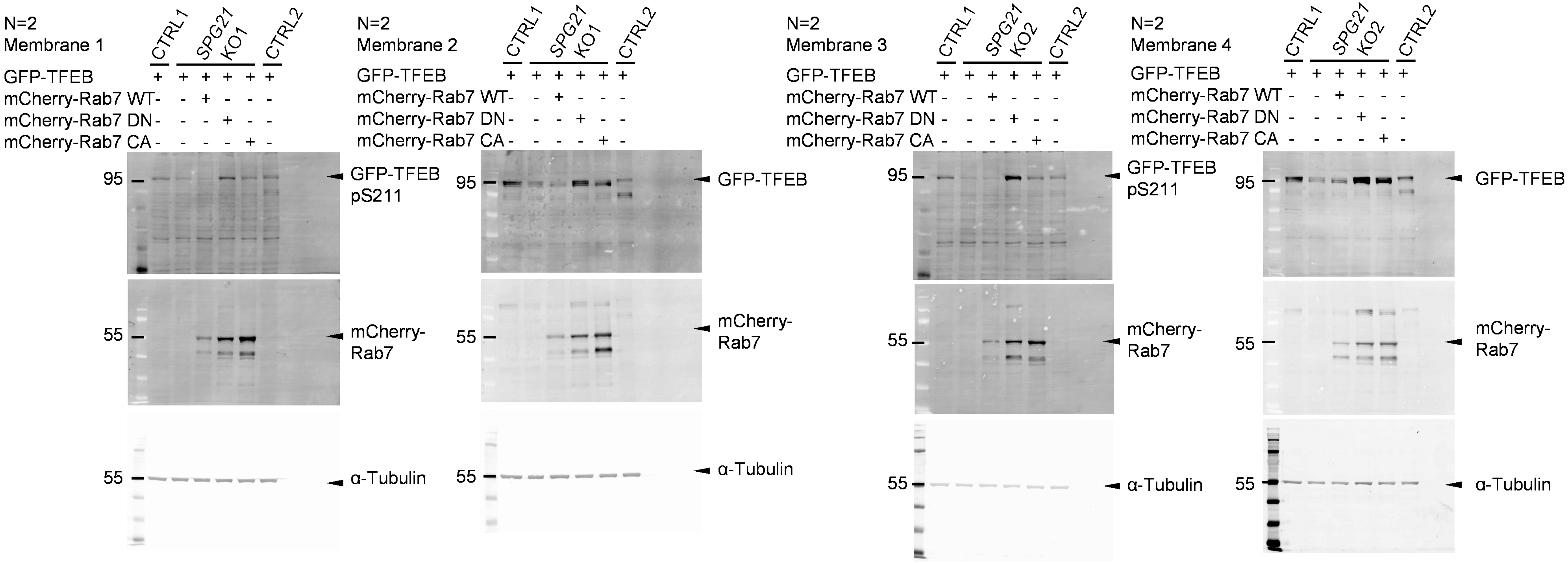

Figure 5C right panel

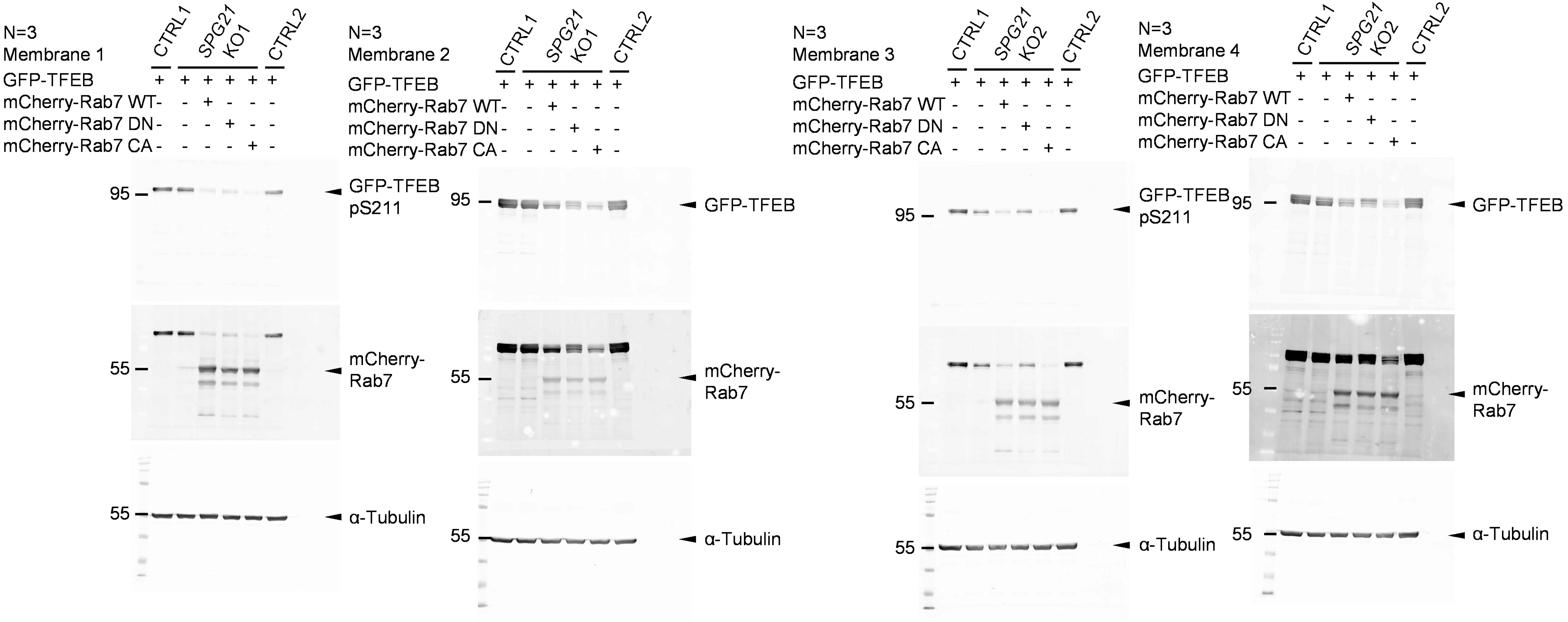

Figure 5C right panel

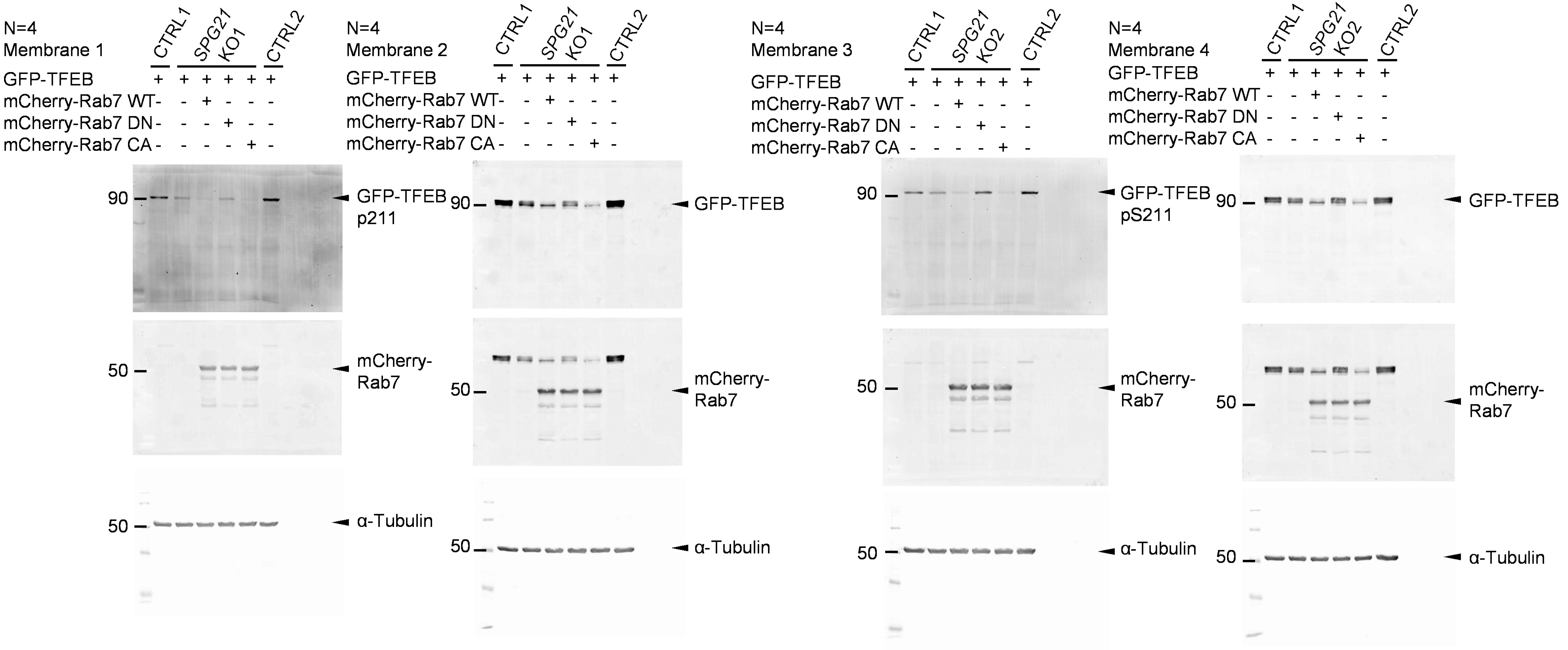

Figure 5C right panel

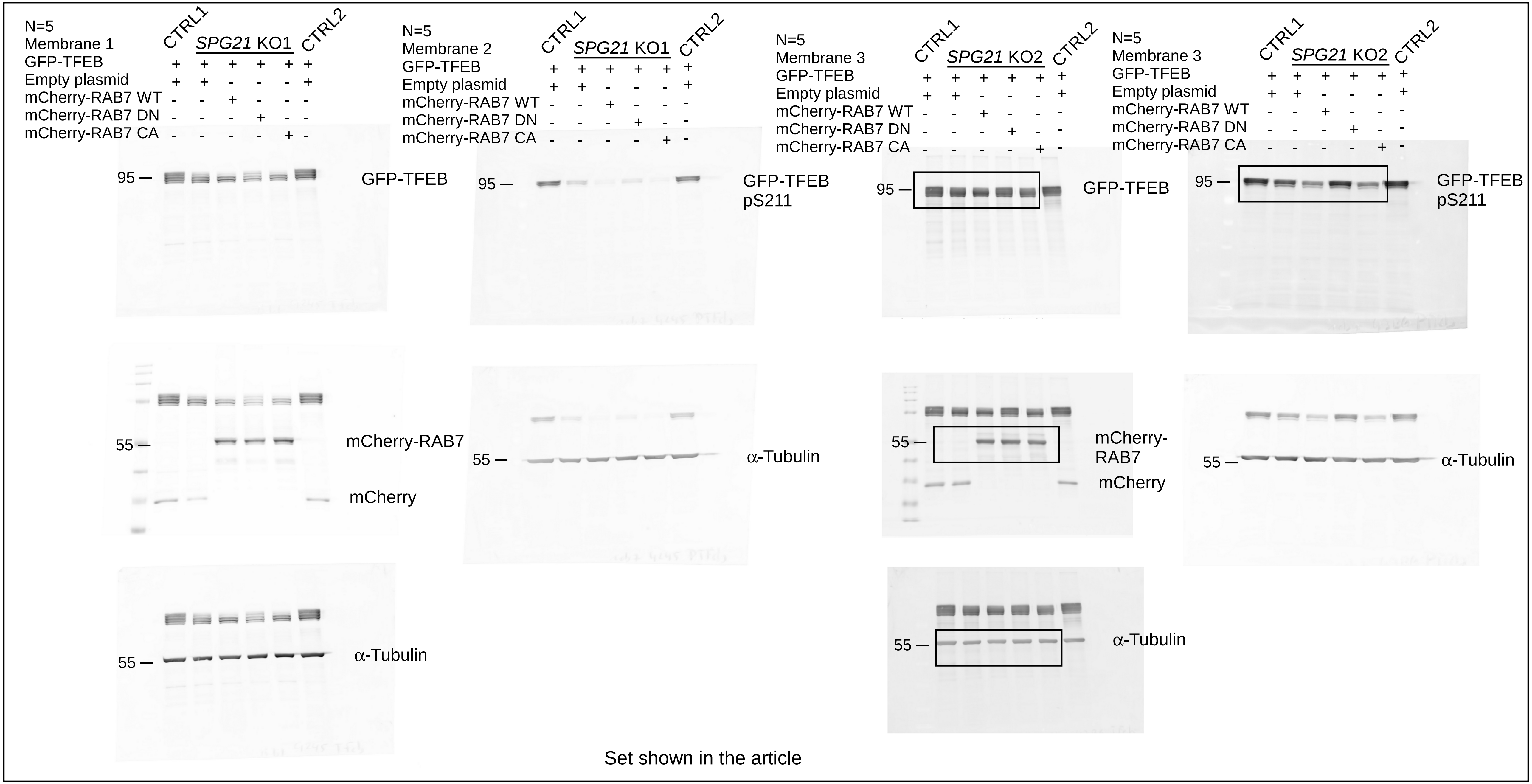

Supplement: SourceData F5 — is the source file for Fig. 5. [file jcb_202501135_sourcedataf5.pdf]
