# Supplementary figures and images for: Maspardin/SPG21 controls lysosome motility and TFEB phosphorylation through RAB7 positioning
Source: J Cell Biol. 2025 Dec 16;225(2):e202501135. doi: 10.1083/jcb.202501135 (PMC12707310; doi:10.1083/jcb.202501135)

Figure 6B

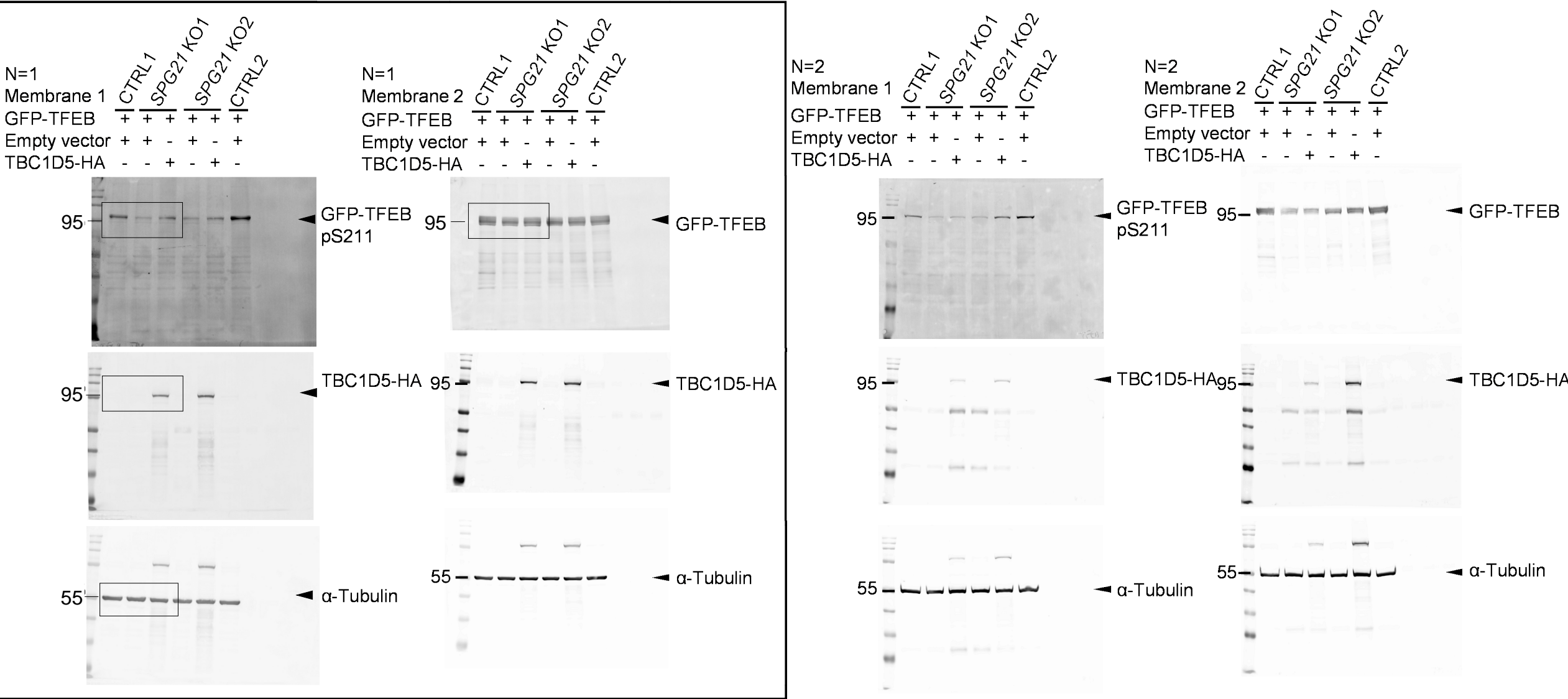

Data shown in the article extracted from this set

Figure 6B

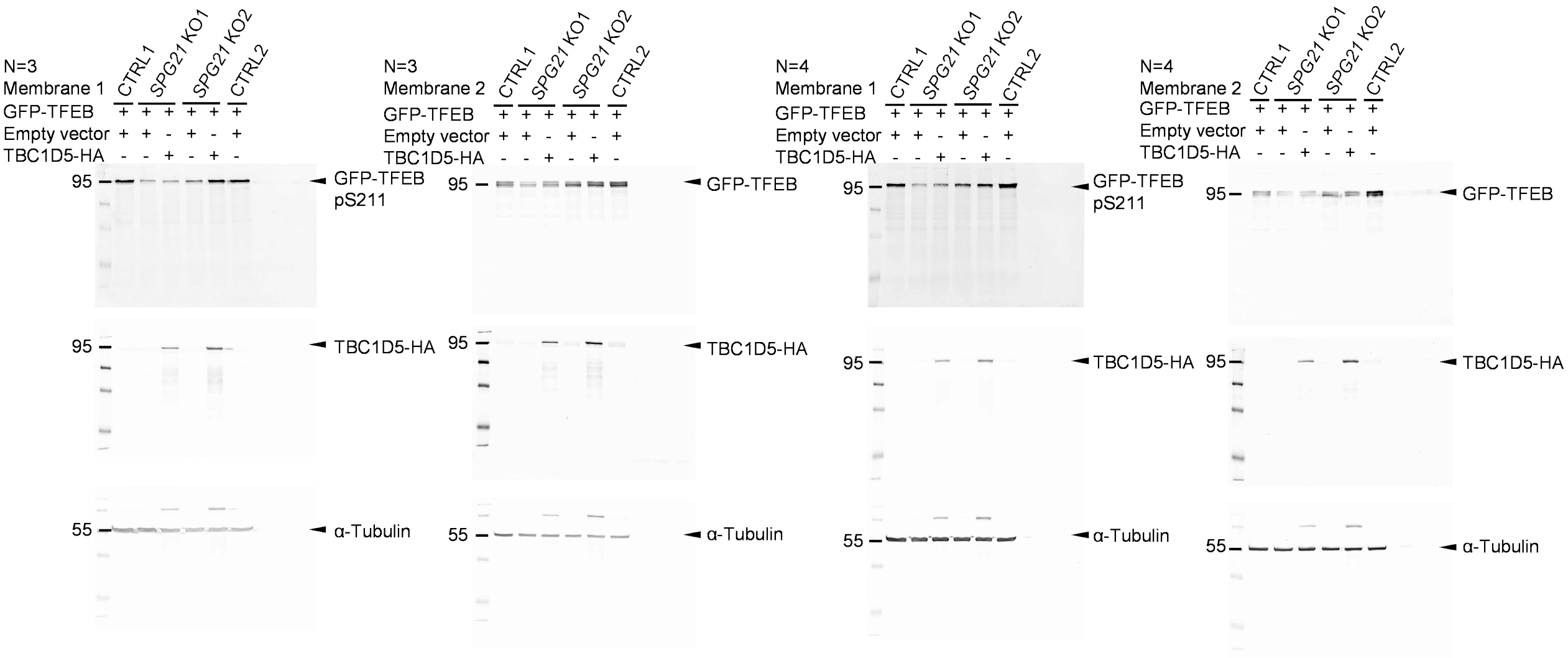

Supplement: SourceData F6 — is the source file for Fig. 6. [file jcb_202501135_sourcedataf6.pdf]

Figure 7D

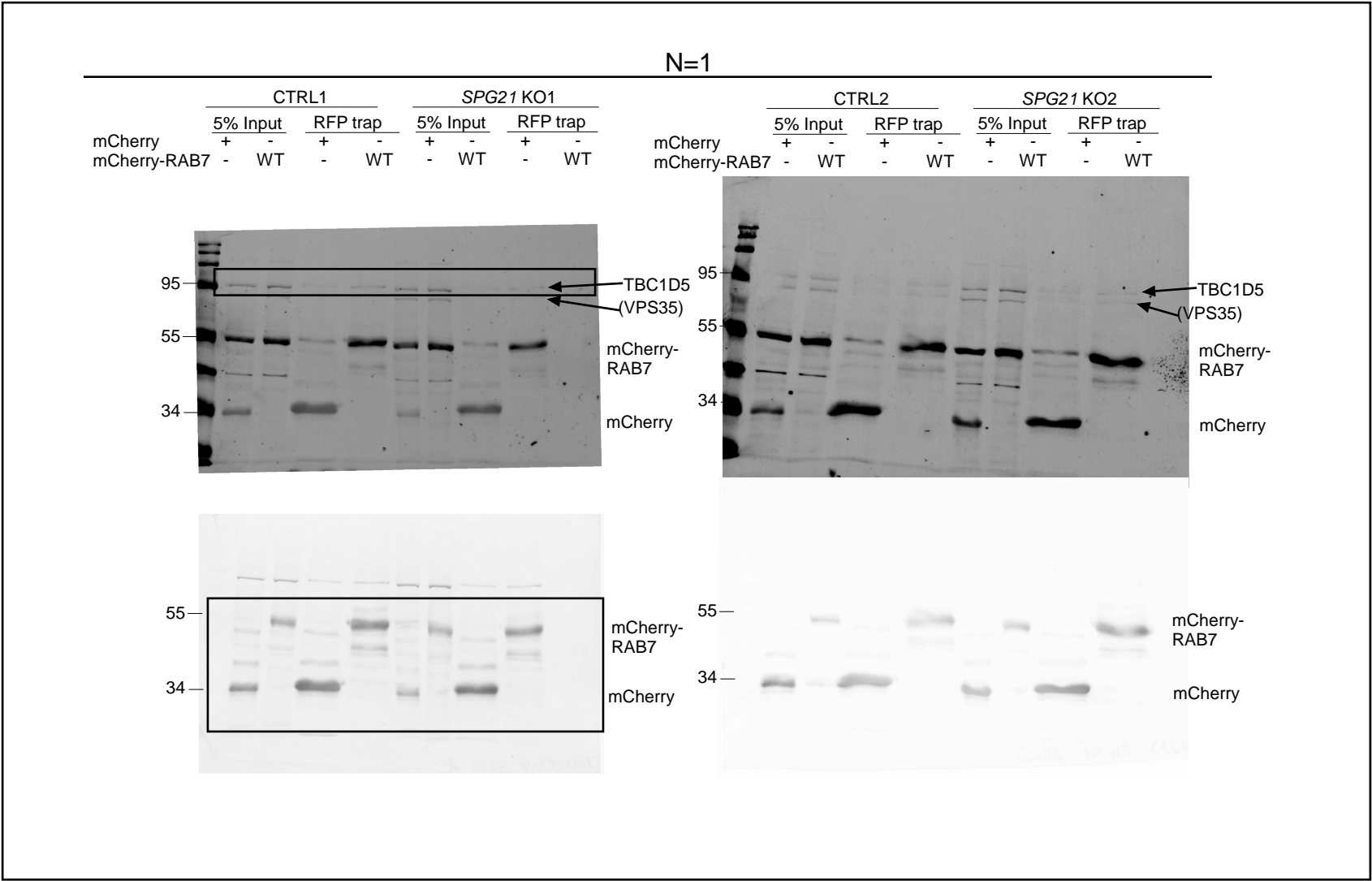

Set shown in the article

Figure 7D

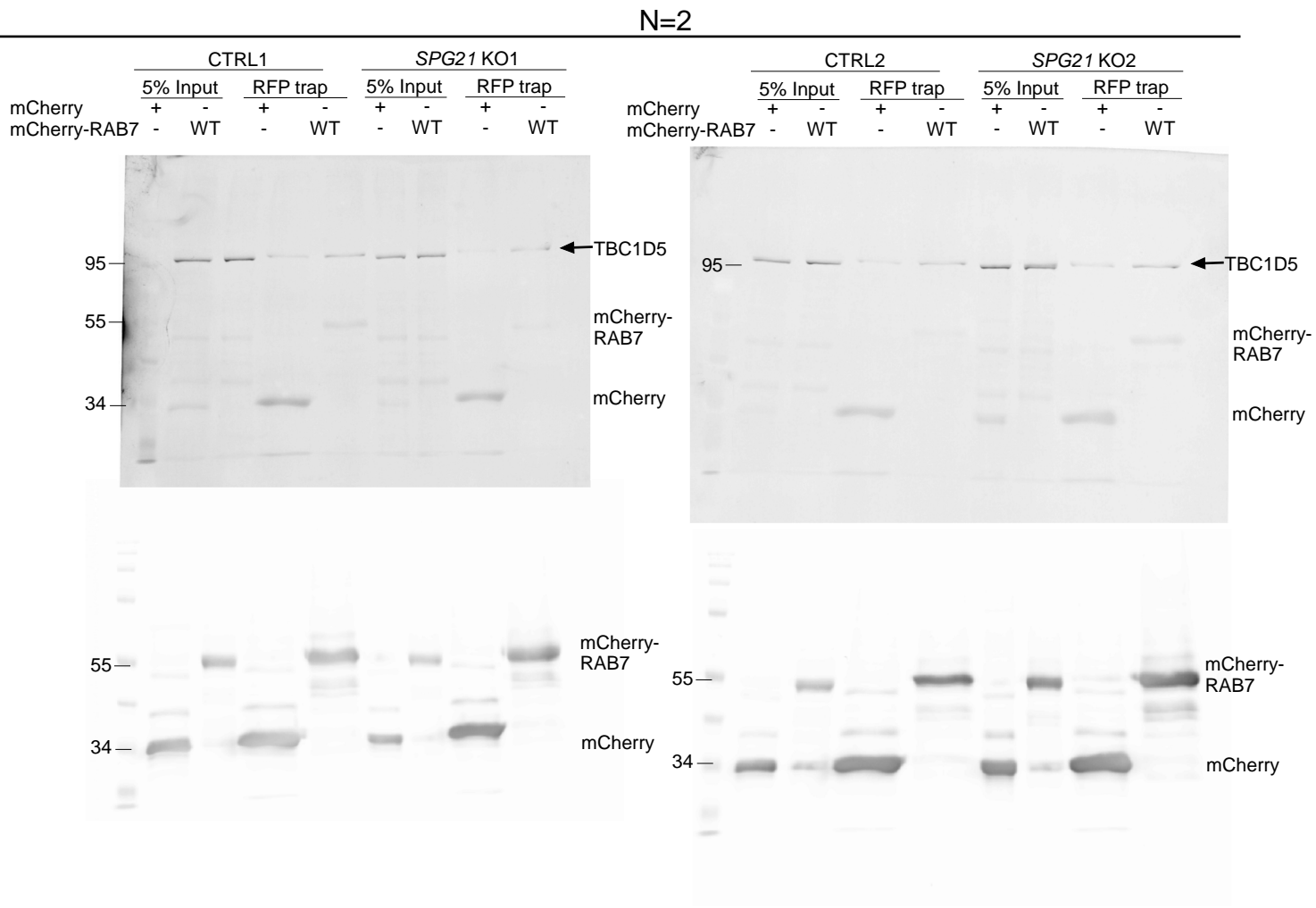

Figure 7D

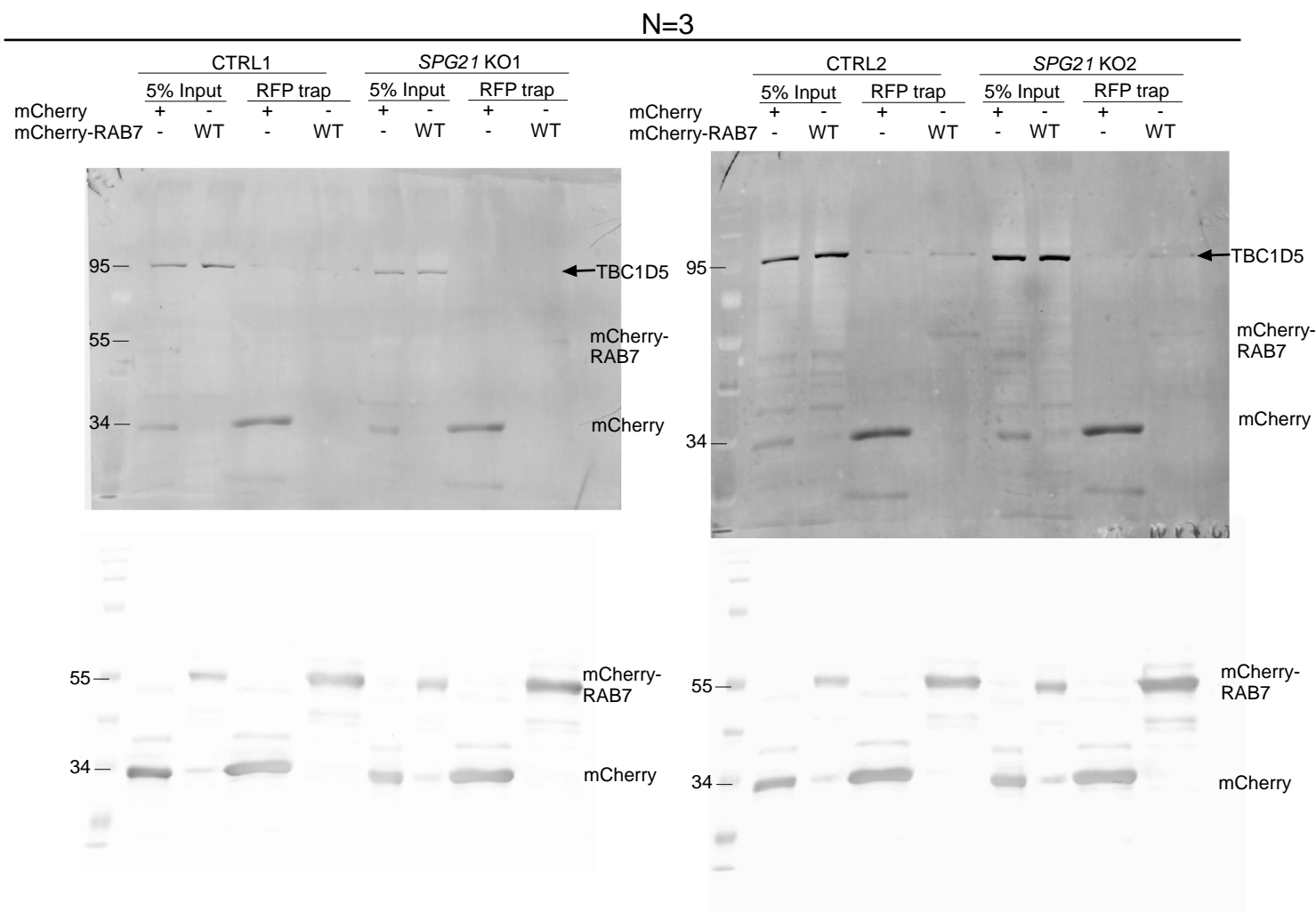

Figure 7D

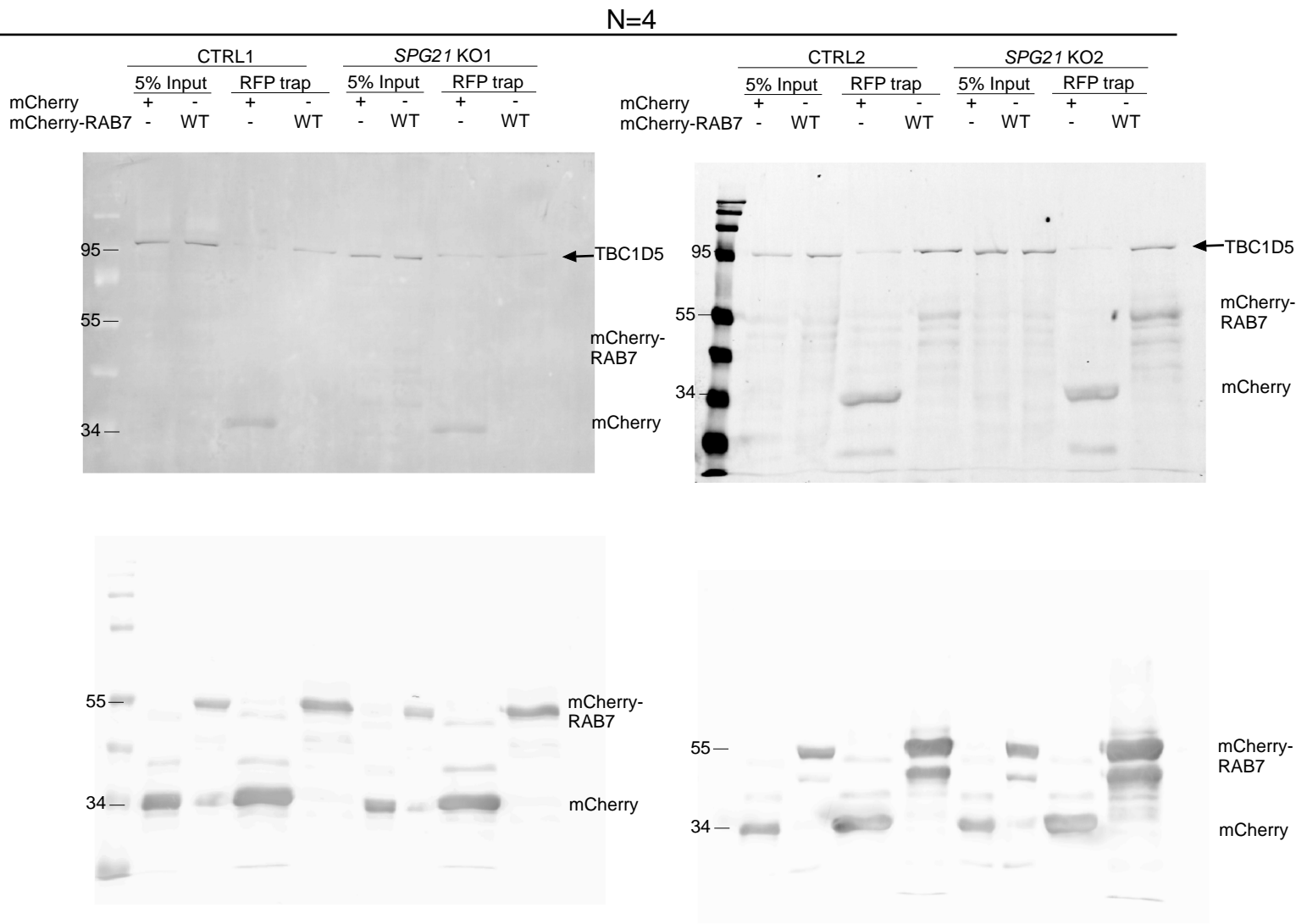

Supplement: SourceData F7 — is the source file for Fig. 7. [file jcb_202501135_sourcedataf7.pdf]
