# Supplementary material for: Maspardin/SPG21 controls lysosome motility and TFEB phosphorylation through RAB7 positioning
Source: J Cell Biol. 2025 Dec 16;225(2):e202501135. doi: 10.1083/jcb.202501135 (PMC12707310; doi:10.1083/jcb.202501135)

## Supplementary Figure 1B

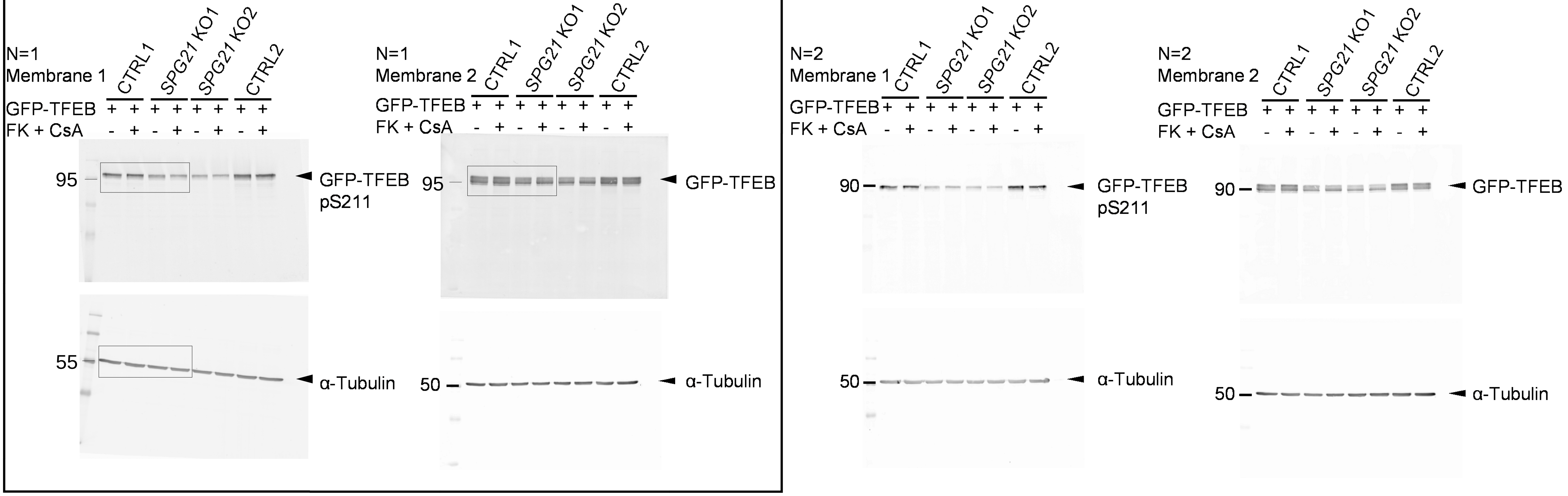


**Data shown in the article extracted from this set**

**Supplementary Figure 1B**

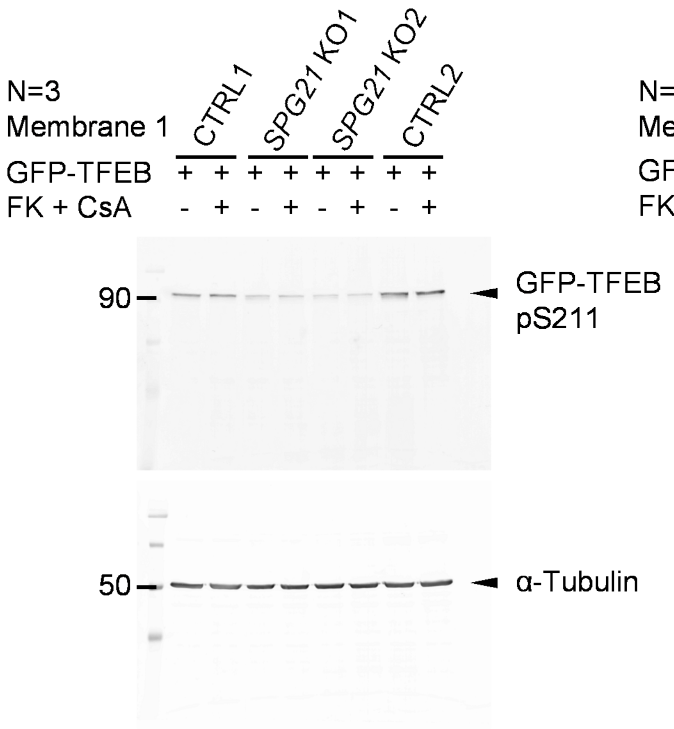


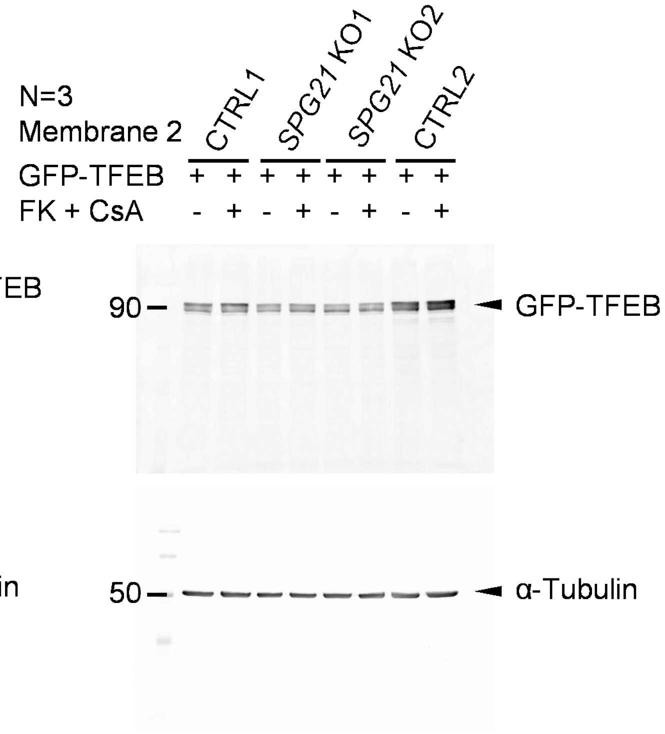

Supplement: SourceData FS1 — is the source file for Fig. S1. [file jcb_202501135_sourcedatafs1.pdf]
