# Supplementary material for: Maspardin/SPG21 controls lysosome motility and TFEB phosphorylation through RAB7 positioning
Source: J Cell Biol. 2025 Dec 16;225(2):e202501135. doi: 10.1083/jcb.202501135 (PMC12707310; doi:10.1083/jcb.202501135)

Figure S2A

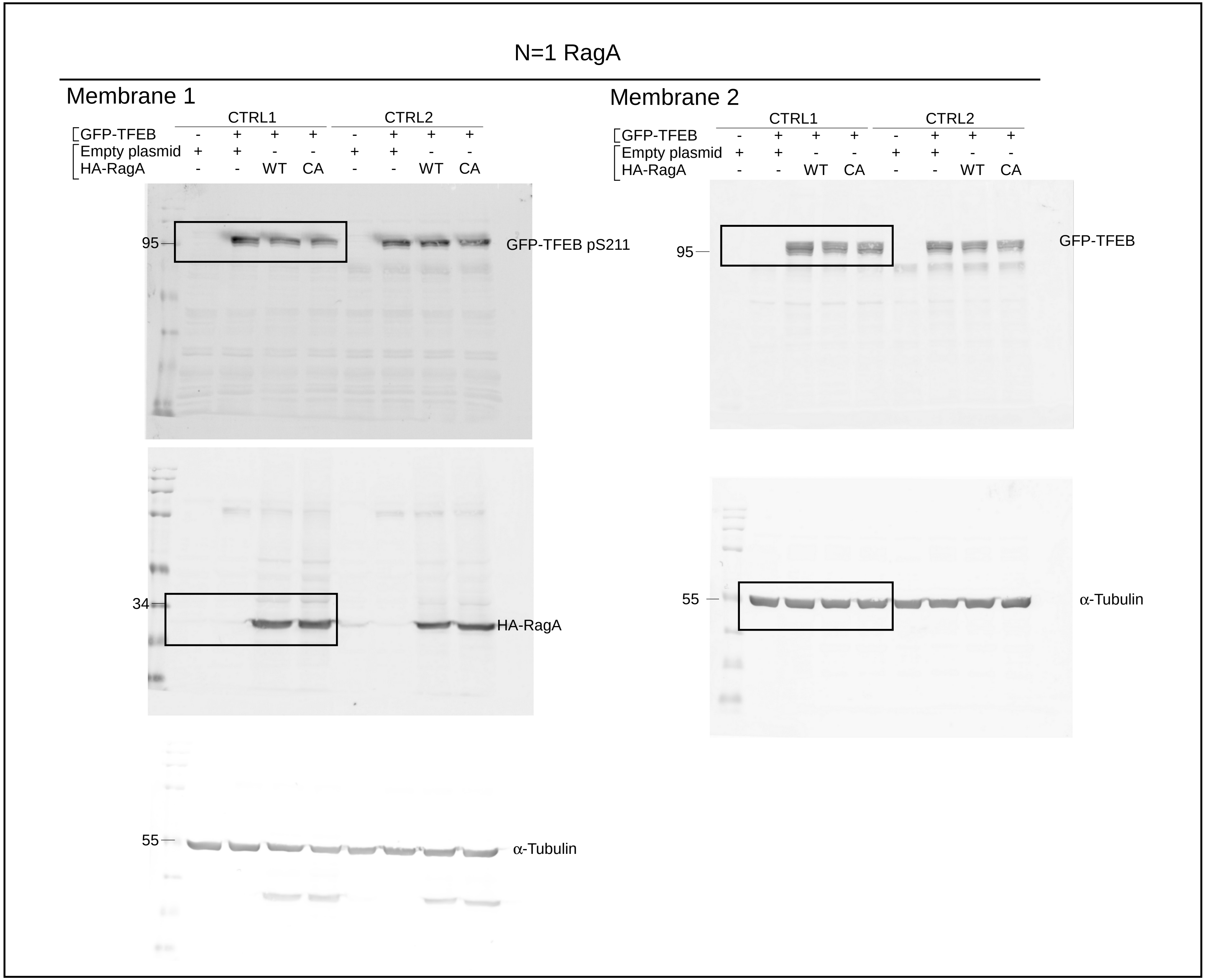

Set shown in the article

Figure S2A

N=1 RagC

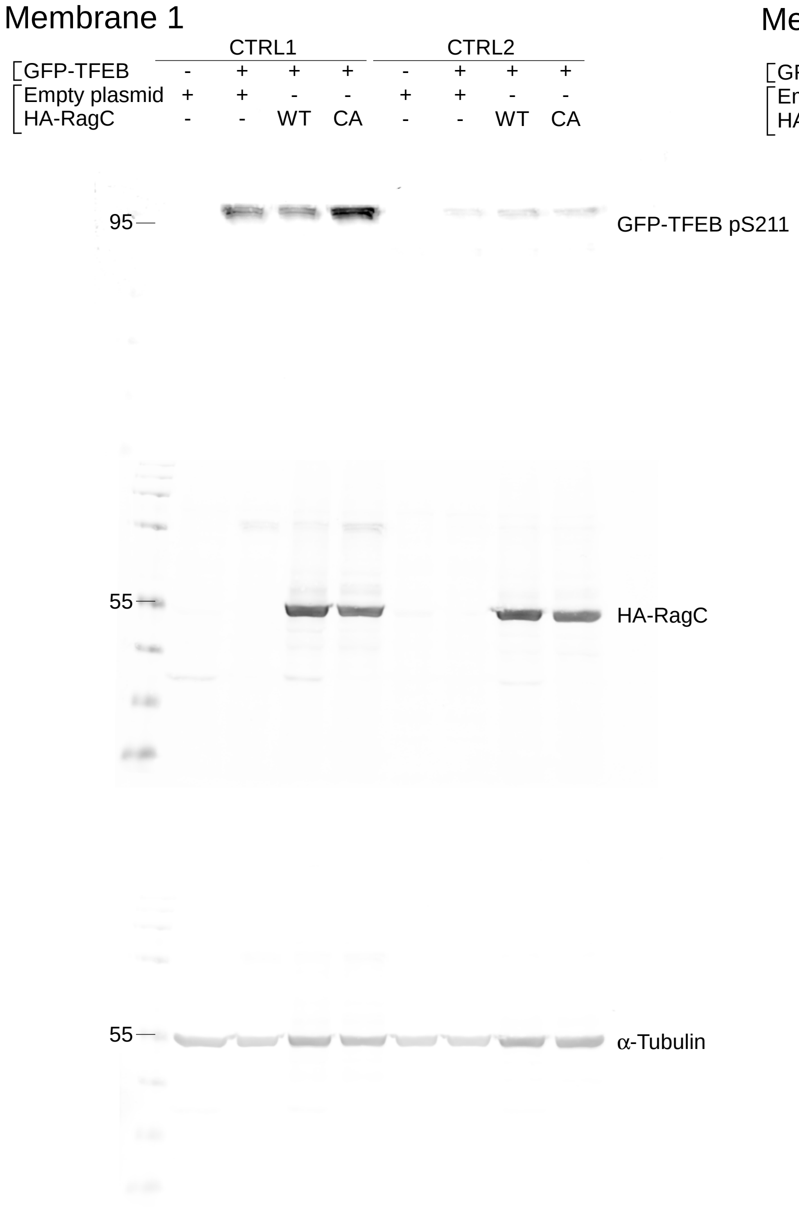

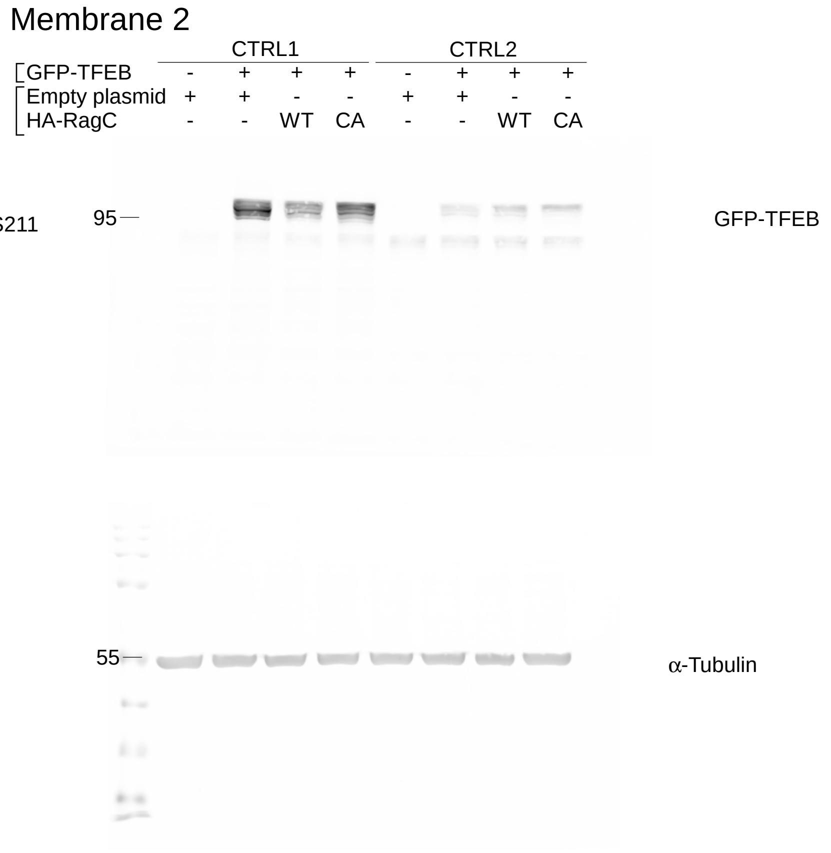

Figure S2A

N=2 RagA

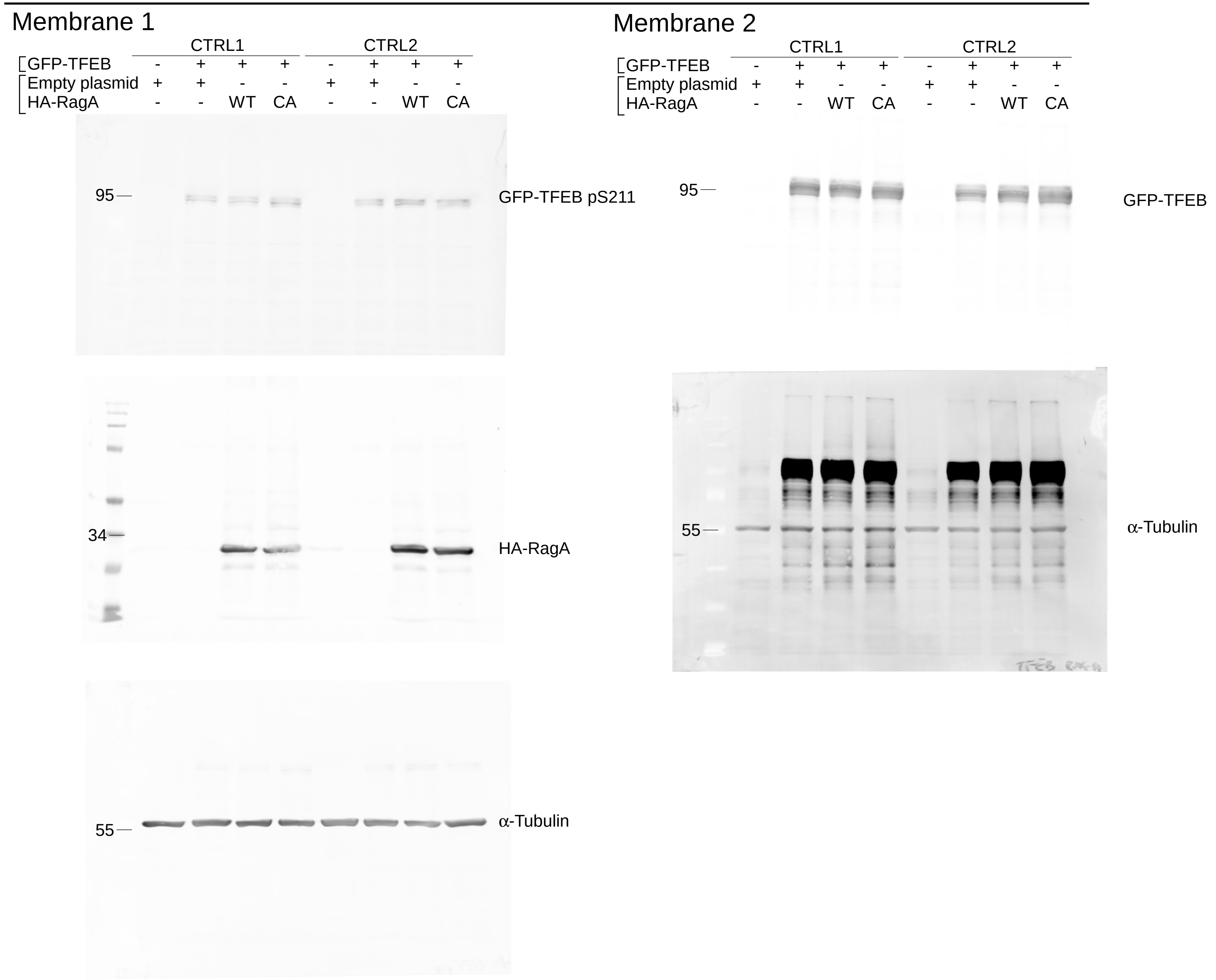

Figure S2A

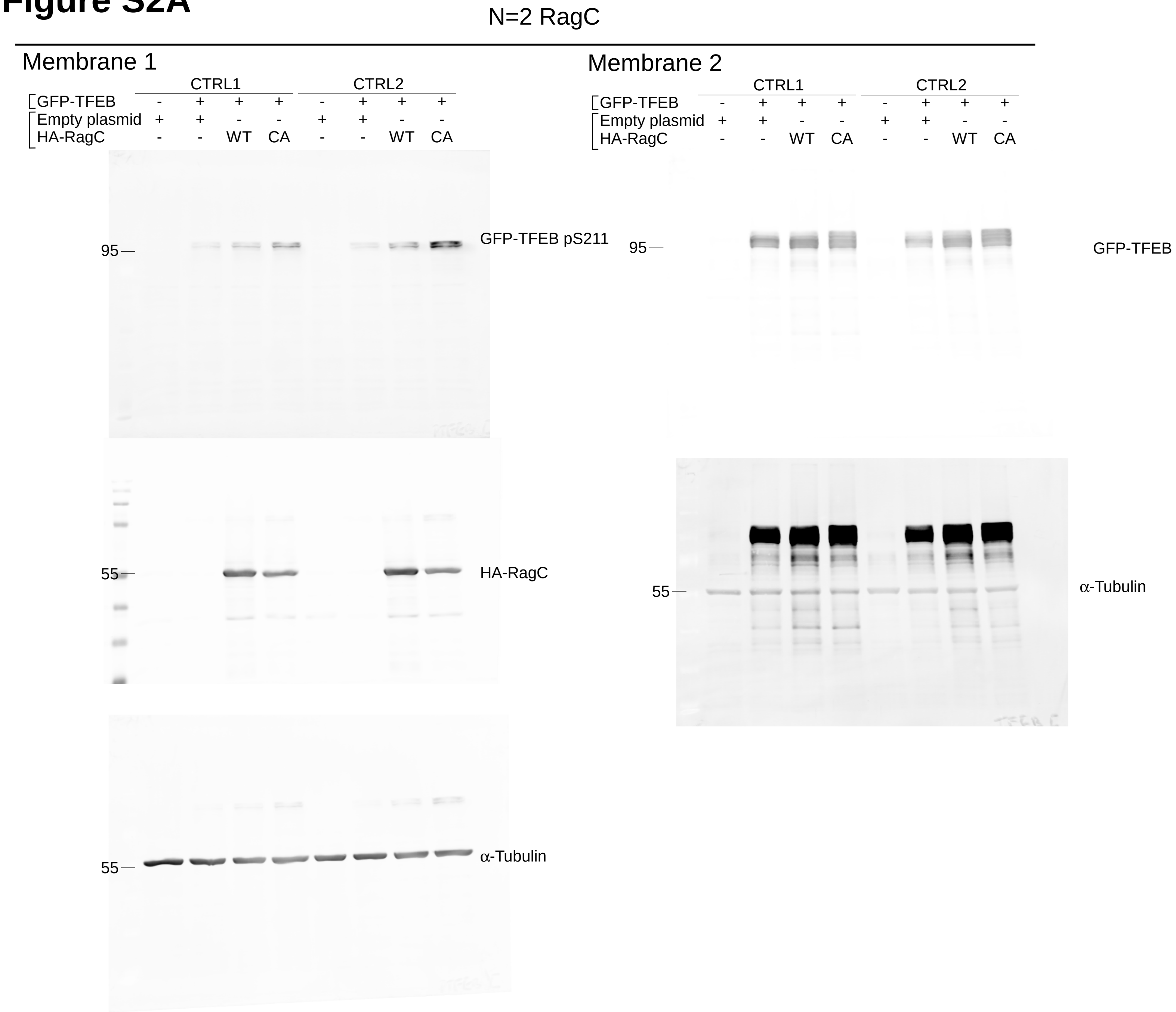

N=2 RagC
Membrane 1
CTRL1
CTRL2
GFP-TFEB - + + + - + + +
Empty plasmid + + - - + + - -
HA-RagC - - WT CA - - WT CA
95
GFP-TFEB pS211
55
HA-RagC
55
α-Tubulin
Membrane 2
CTRL1
CTRL2
GFP-TFEB - + + + - + + +
Empty plasmid + + - - + + - -
HA-RagC - - WT CA - - WT CA
95
GFP-TFEB
55
α-Tubulin

Figure S2A

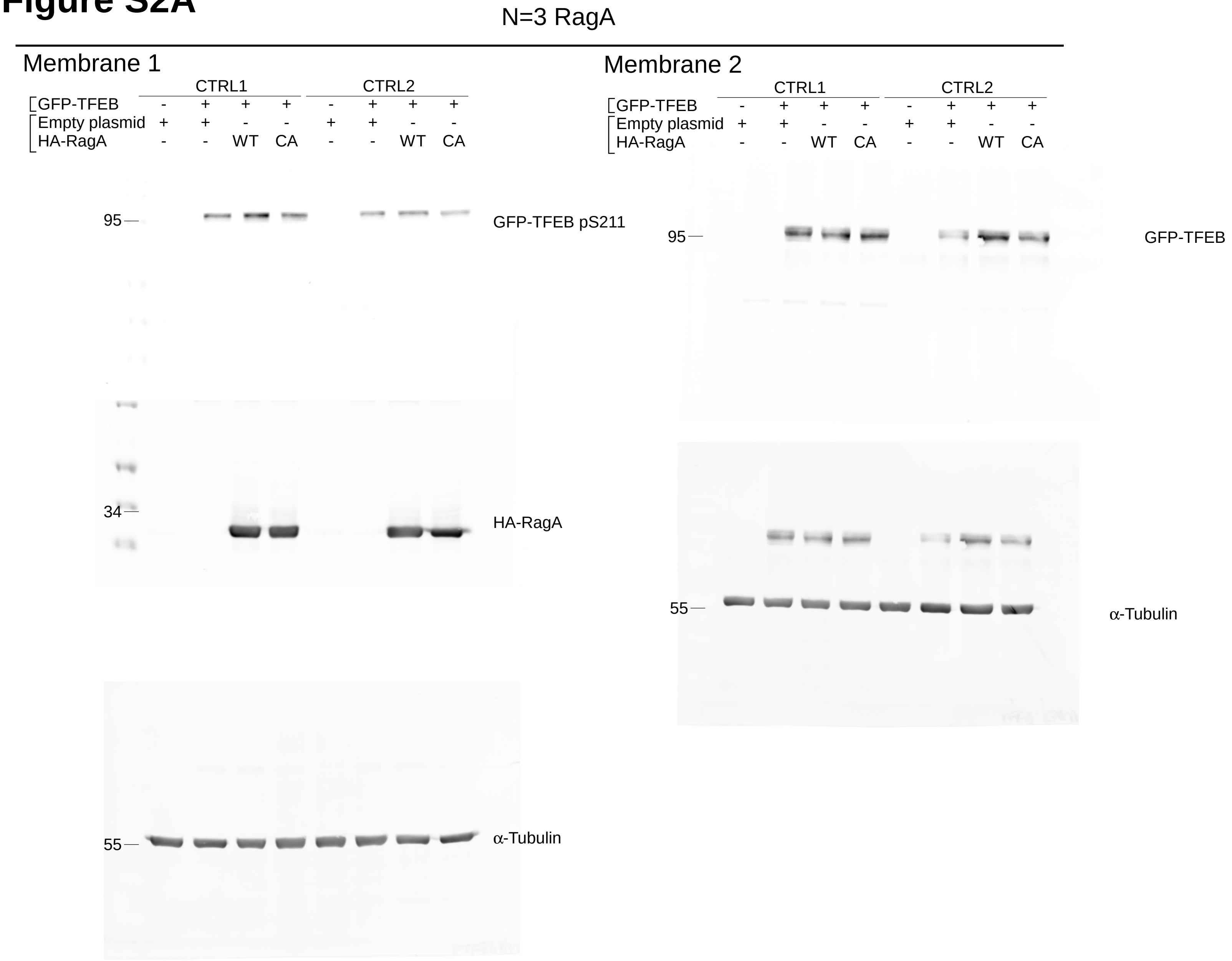
N=3 RagA
Membrane 1
CTRL1
CTRL2
GFP-TFEB - + + + - + + +
Empty plasmid + + - - + + - -
HA-RagA - - WT CA - - WT CA
95
GFP-TFEB pS211
34
HA-RagA
55
α-Tubulin
Membrane 2
CTRL1
CTRL2
GFP-TFEB - + + + - + + +
Empty plasmid + + - - + + - -
HA-RagA - - WT CA - - WT CA
95
GFP-TFEB
55
α-Tubulin

Figure S2A

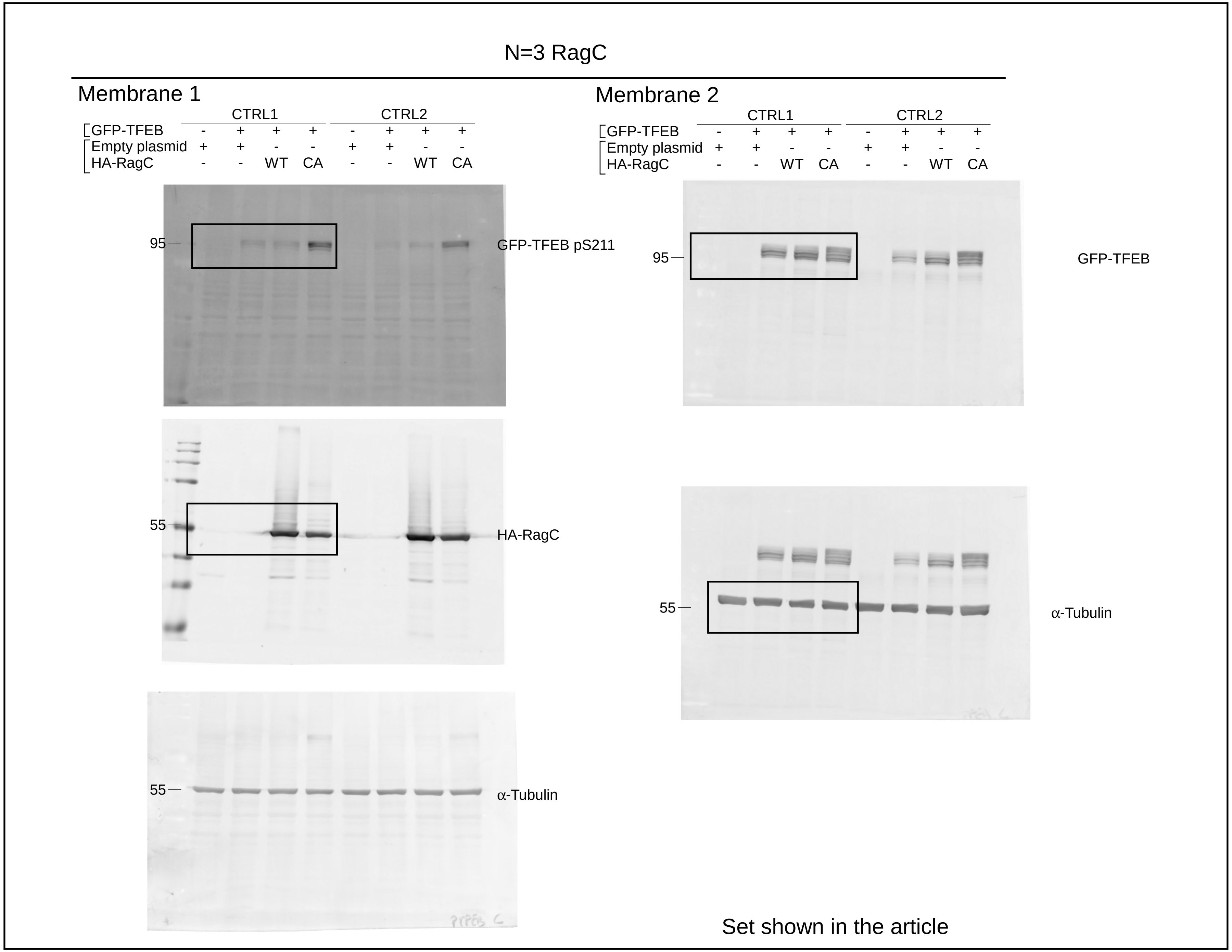
N=3 RagC
Membrane 1
CTRL1
CTRL2
GFP-TFEB - + + + - + + +
Empty plasmid + + - - + + - -
HA-RagC - - WT CA - - WT CA
95
GFP-TFEB pS211
55
HA-RagC
55
α-Tubulin
Membrane 2
CTRL1
CTRL2
GFP-TFEB - + + + - + + +
Empty plasmid + + - - + + - -
HA-RagC - - WT CA - - WT CA
95
GFP-TFEB
55
α-Tubulin
Set shown in the article

Figure S2C

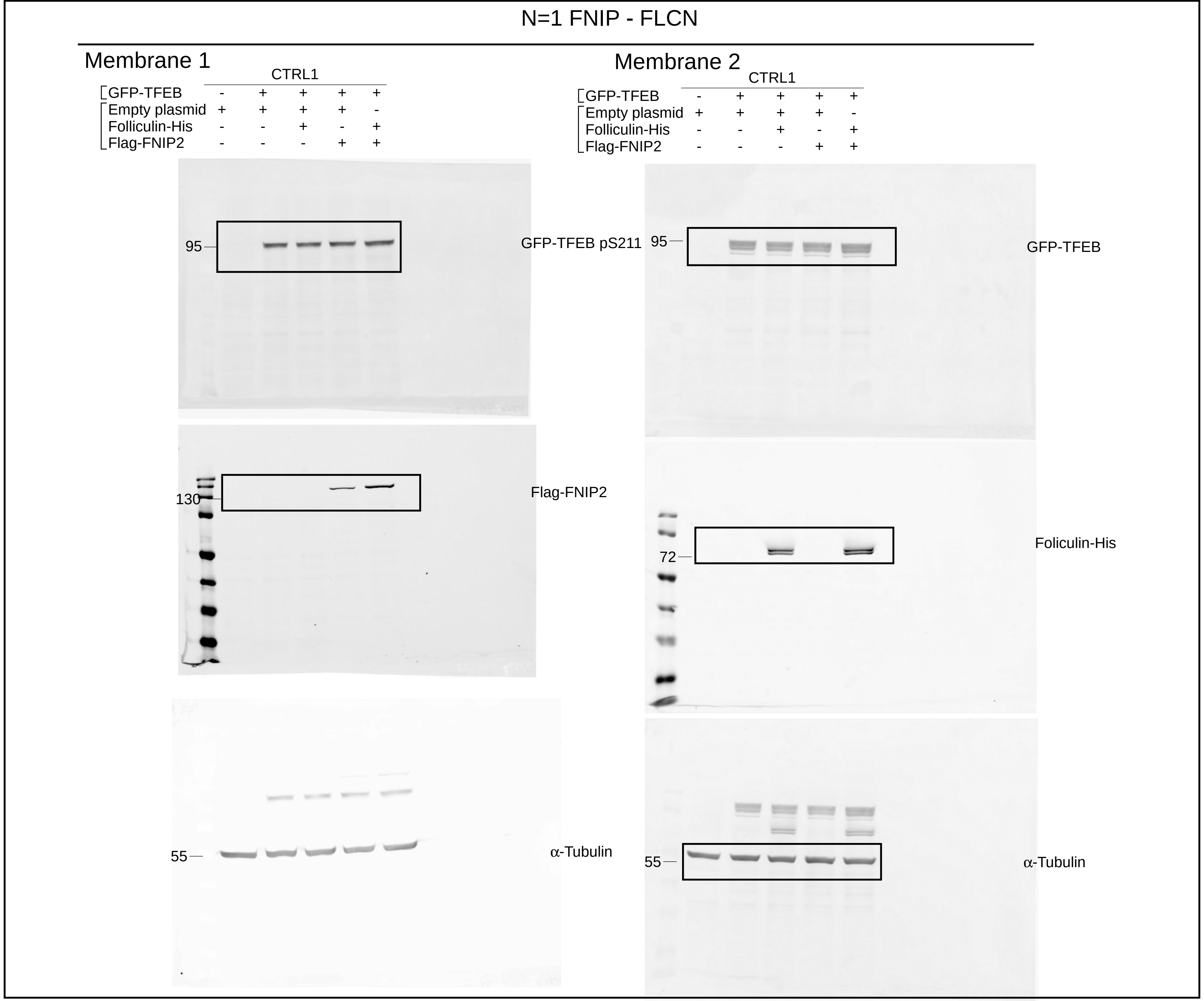

Set shown in the article

Figure S2C

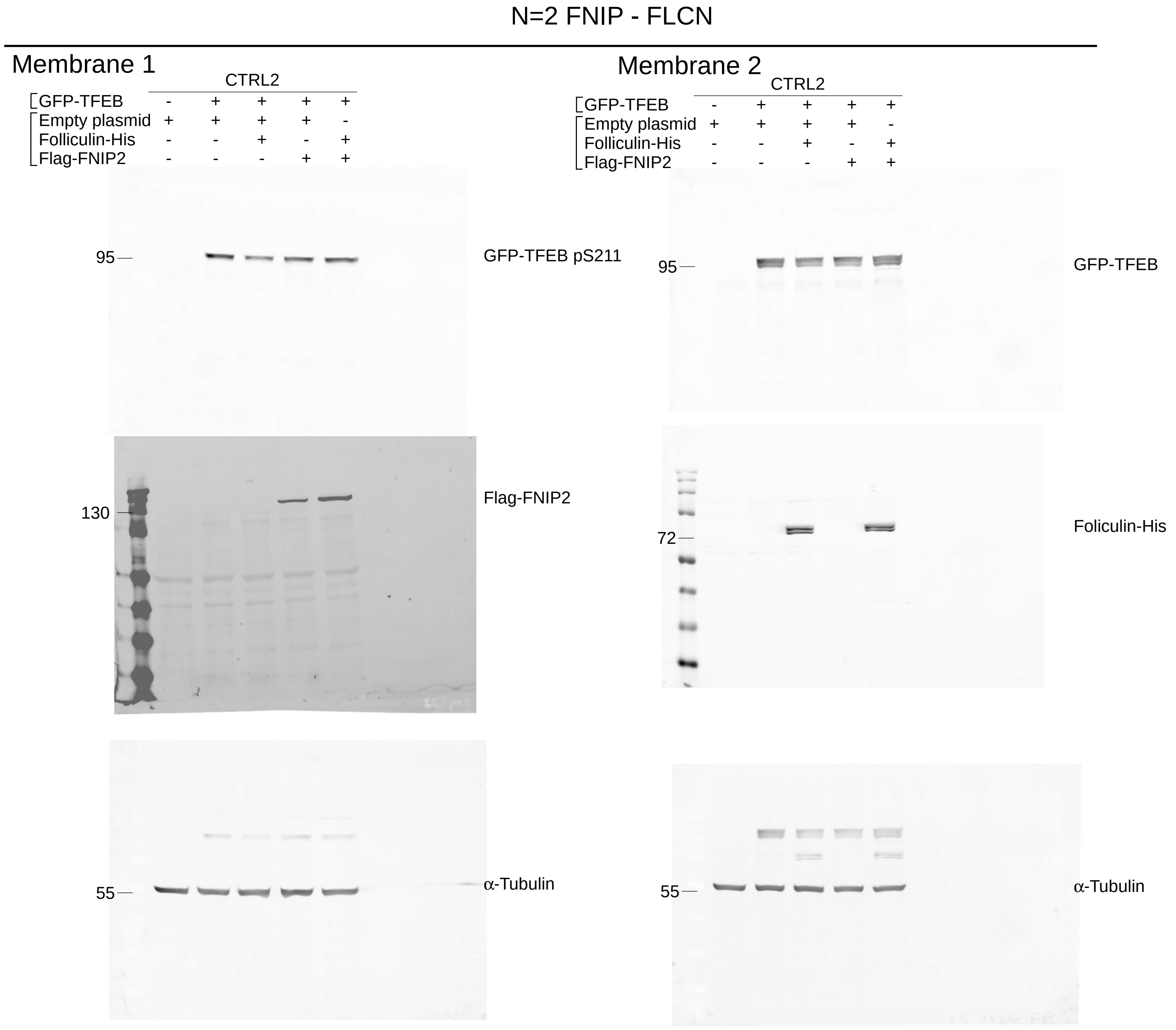
N=2 FNIP - FLCN
Membrane 1
CTRL2
GFP-TFEB - + + + +
Empty plasmid + + + + -
Folliculin-His - - + - +
Flag-FNIP2 - - - + +
95
GFP-TFEB pS211
130
Flag-FNIP2
55
α-Tubulin
Membrane 2
CTRL2
GFP-TFEB - + + + +
Empty plasmid + + + + -
Folliculin-His - - + - +
Flag-FNIP2 - - - + +
95
GFP-TFEB
72
Foliculin-His
55
α-Tubulin

Figure S2C

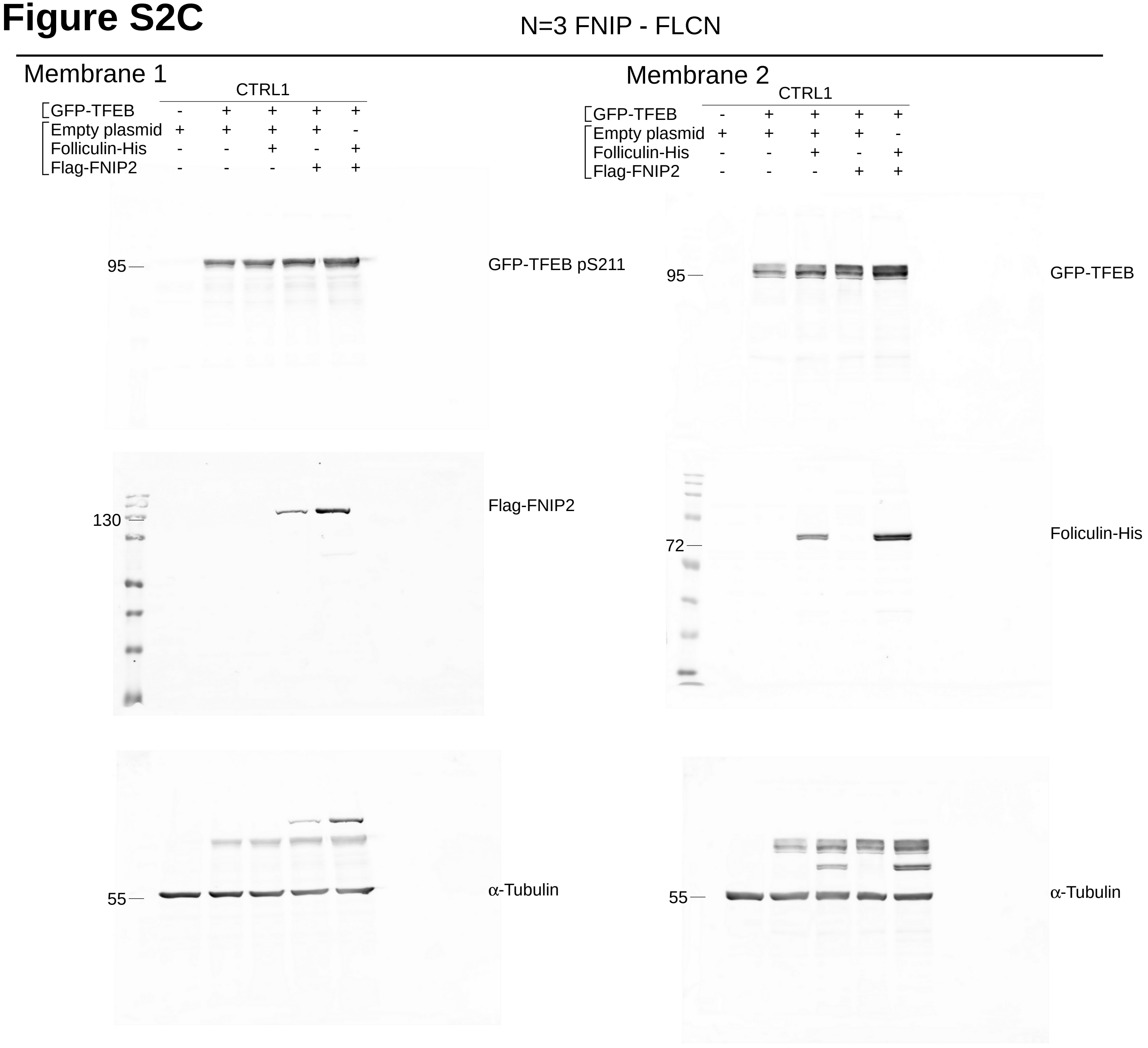
N=3 FNIP - FLCN
Membrane 1
CTRL1
GFP-TFEB - + + + +
Empty plasmid + + + + -
Folliculin-His - - + - +
Flag-FNIP2 - - - + +
95
GFP-TFEB pS211
130
Flag-FNIP2
55
α-Tubulin
Membrane 2
CTRL1
GFP-TFEB - + + + +
Empty plasmid + + + + -
Folliculin-His - - + - +
Flag-FNIP2 - - - + +
95
GFP-TFEB
72
Foliculin-His
55
α-Tubulin

Figure S2C

N=4 FNIP - FLCN

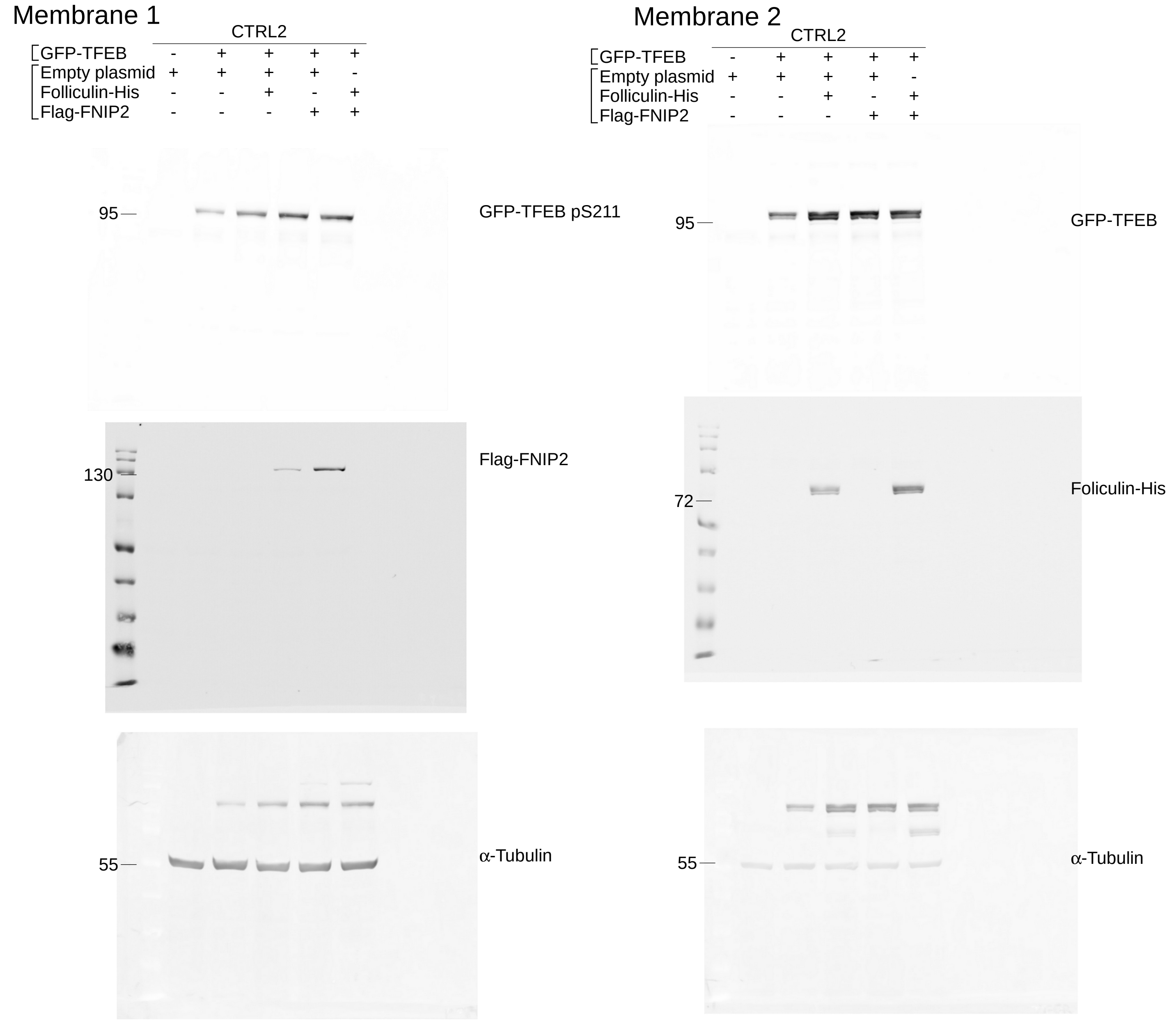

Figure S2D

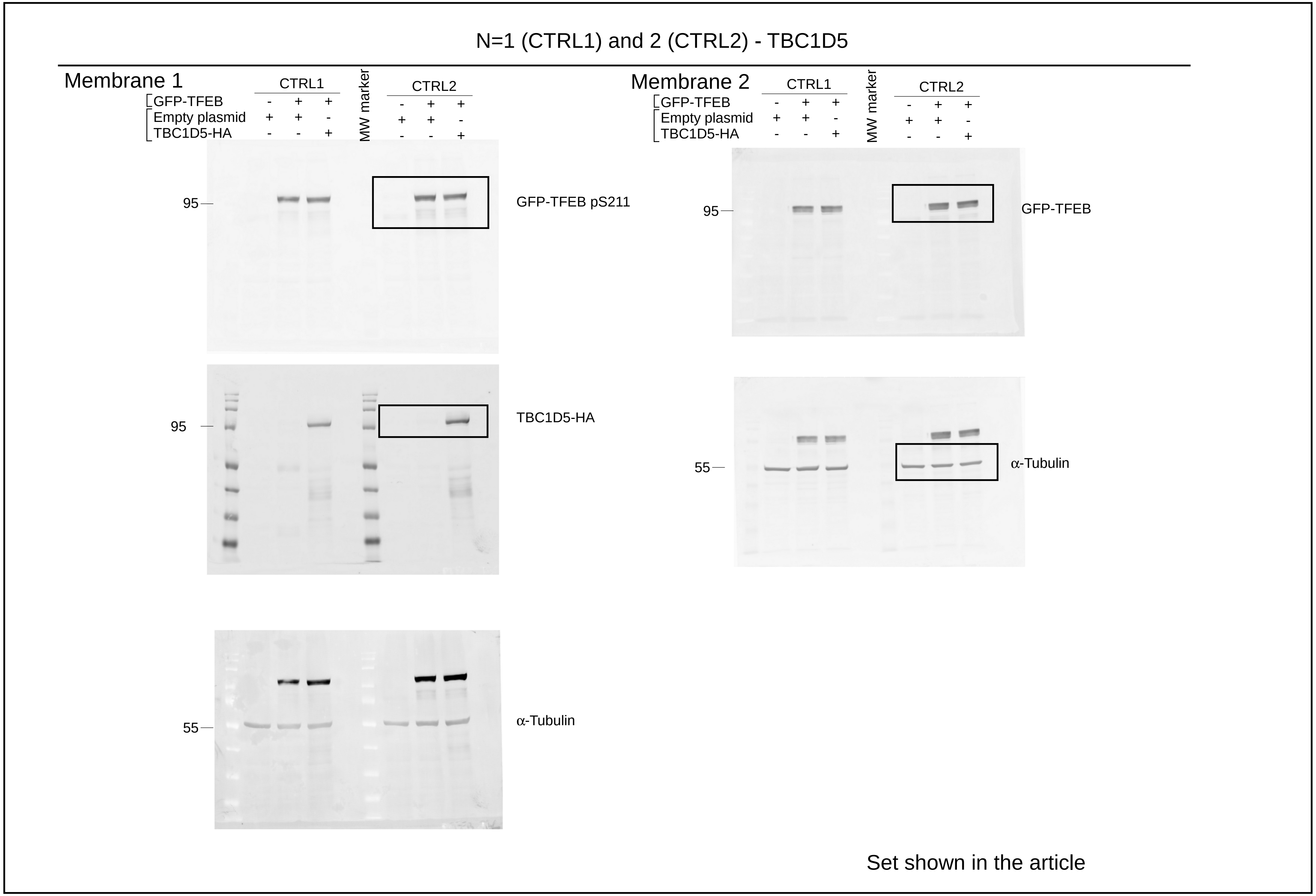
N=1 (CTRL1) and 2 (CTRL2) - TBC1D5
Membrane 1
CTRL1
CTRL2
MW marker
GFP-TFEB - + + - + +
Empty plasmid + + - + + -
TBC1D5-HA - - + - - +
95
GFP-TFEB pS211
95
TBC1D5-HA
55
α-Tubulin
Membrane 2
CTRL1
CTRL2
MW marker
GFP-TFEB - + + - + +
Empty plasmid + + - + + -
TBC1D5-HA - - + - - +
95
GFP-TFEB
55
α-Tubulin
Set shown in the article

Figure S2D

N=3 (CTRL1) - TBC1D5

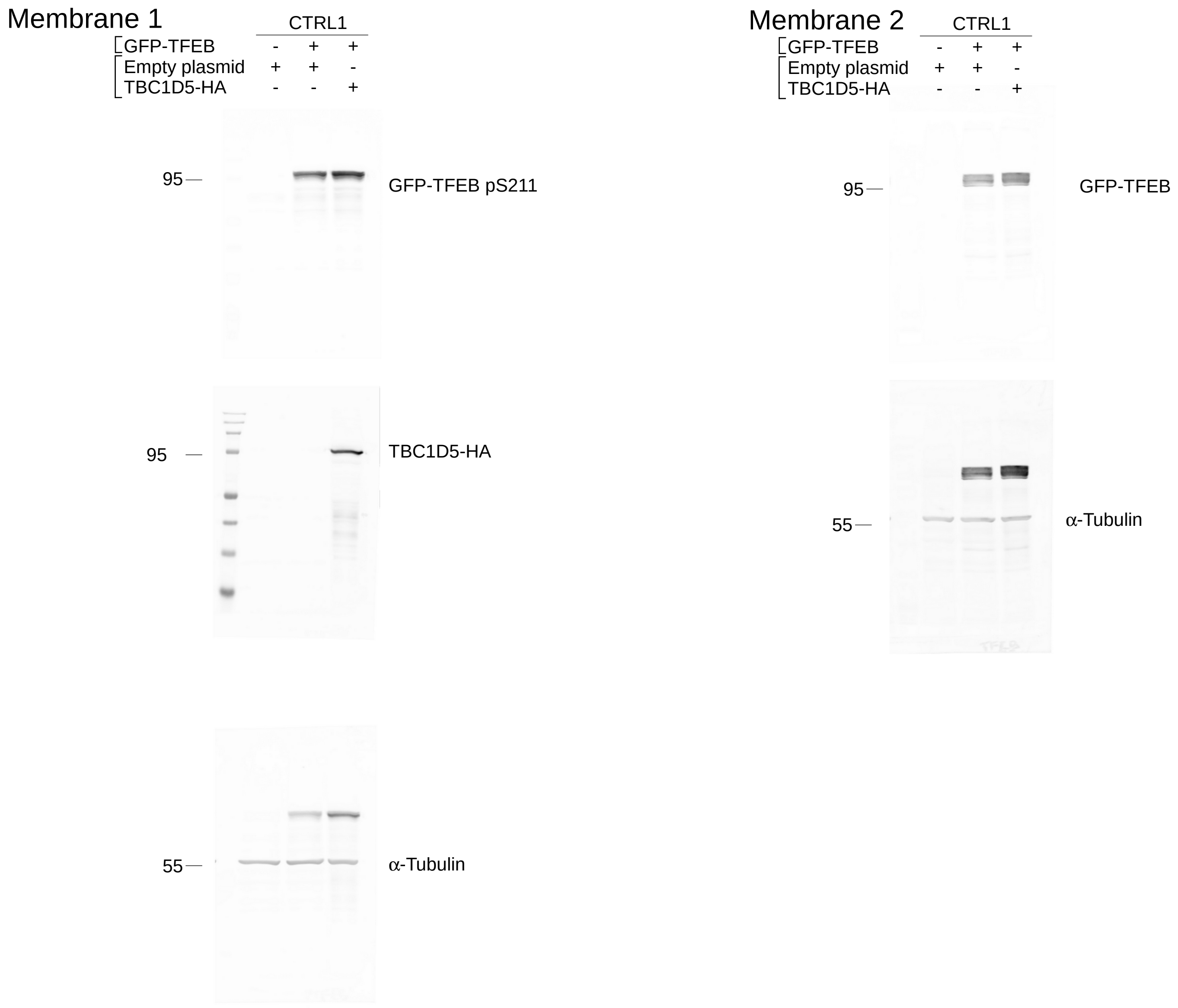

Figure S2D

N=4 (CTRL2) - TBC1D5

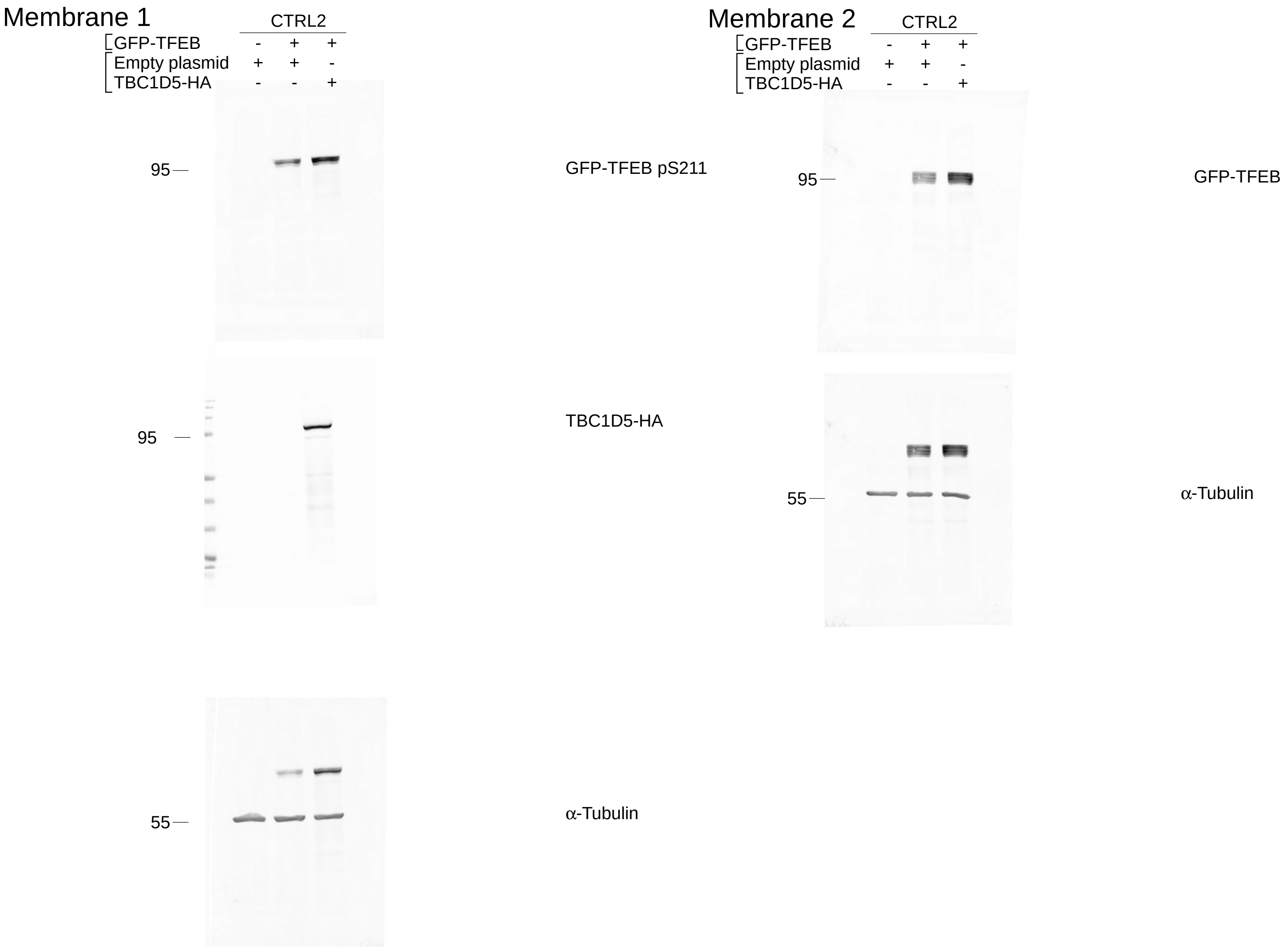

Supplement: SourceData FS2 — is the source file for Fig. S2. [file jcb_202501135_sourcedatafs2.pdf]
