# Supplementary material for: Maspardin/SPG21 controls lysosome motility and TFEB phosphorylation through RAB7 positioning
Source: J Cell Biol. 2025 Dec 16;225(2):e202501135. doi: 10.1083/jcb.202501135 (PMC12707310; doi:10.1083/jcb.202501135)
Supplement: SourceData FS3 — is the source file for Fig. S3. [file jcb_202501135_sourcedatafs3.pdf]

Figure S3A

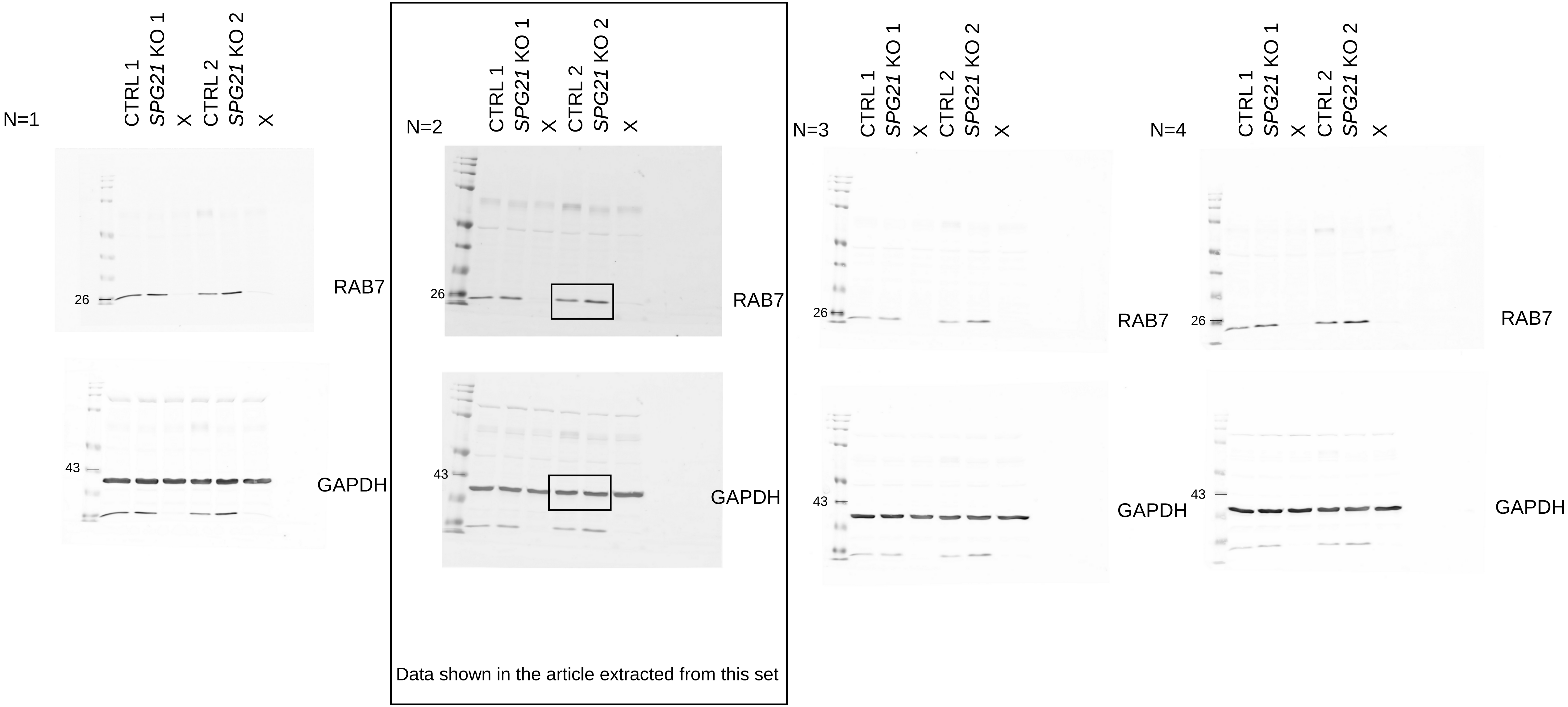
N=1
CTRL 1
SPG21 KO 1
X
CTRL 2
SPG21 KO 2
X
26
RAB7
43
GAPDH
N=2
CTRL 1
SPG21 KO 1
X
CTRL 2
SPG21 KO 2
X
26
RAB7
43
GAPDH
Data shown in the article extracted from this set
N=3
CTRL 1
SPG21 KO 1
X
CTRL 2
SPG21 KO 2
X
26
RAB7
43
GAPDH
N=4
CTRL 1
SPG21 KO 1
X
CTRL 2
SPG21 KO 2
X
26
RAB7
43
GAPDH

Figure S3B N=1

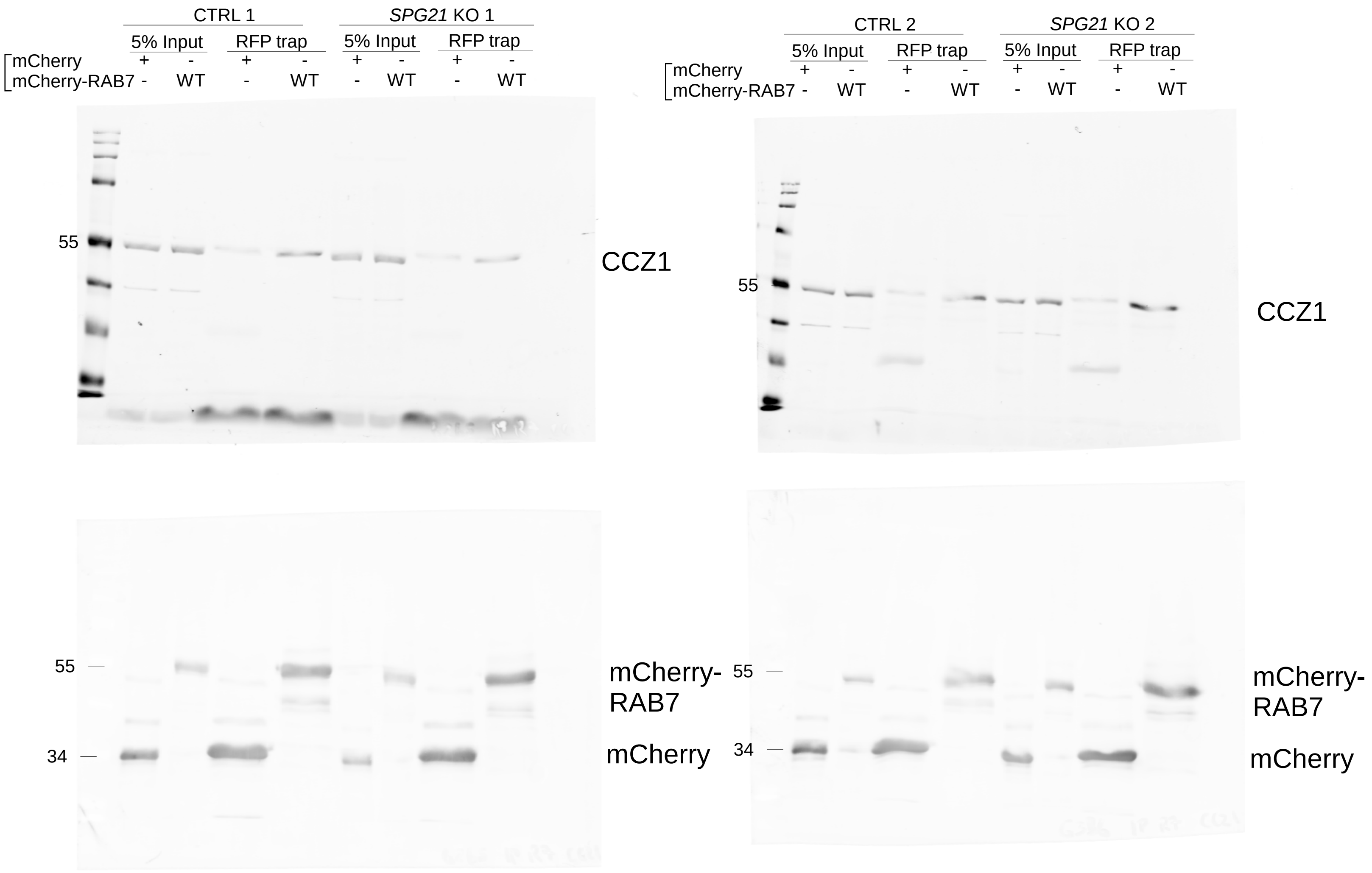

Figure S3B

N=2

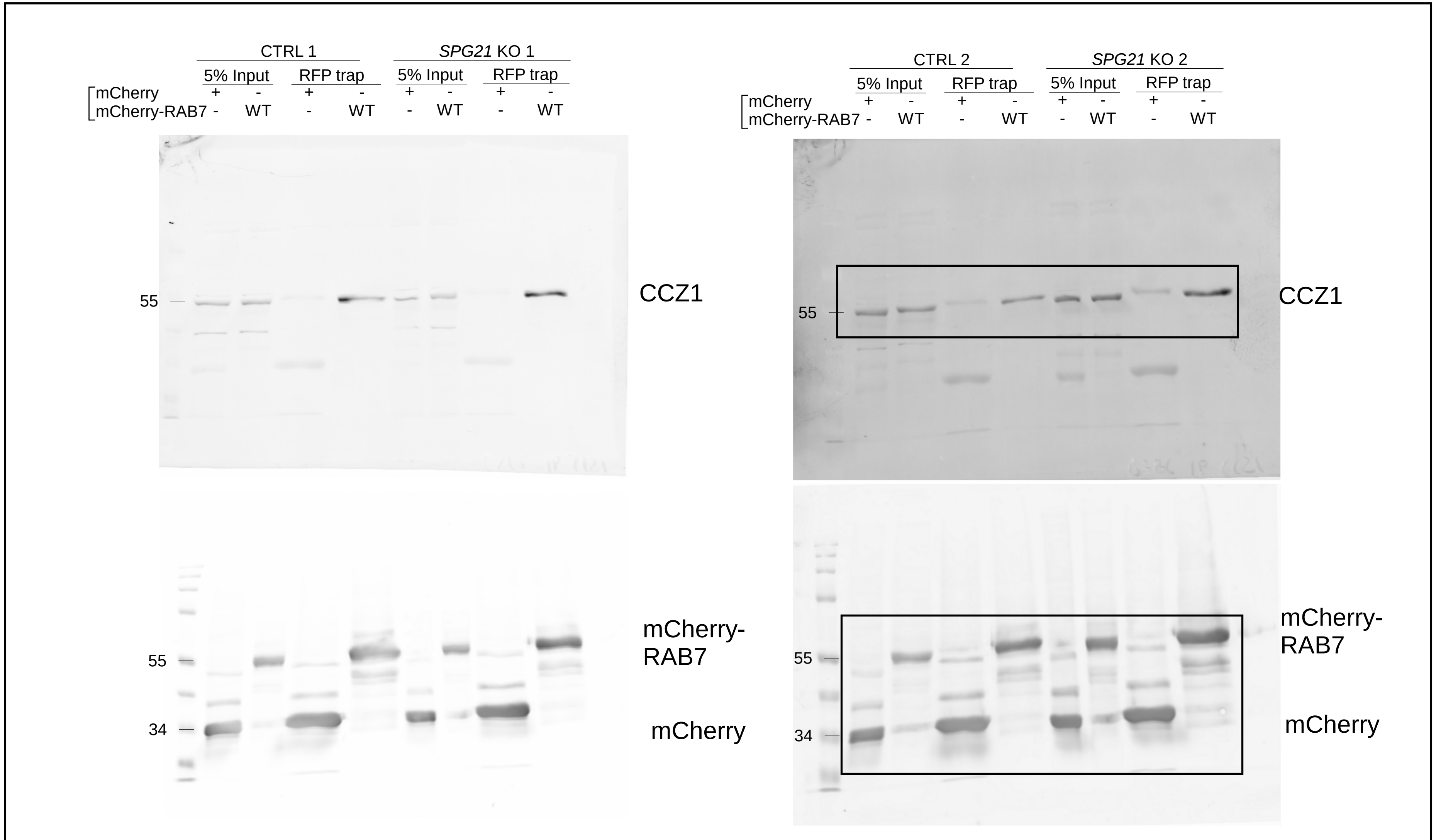

Data shown in the article extracted from this set

Figure S3B N=3

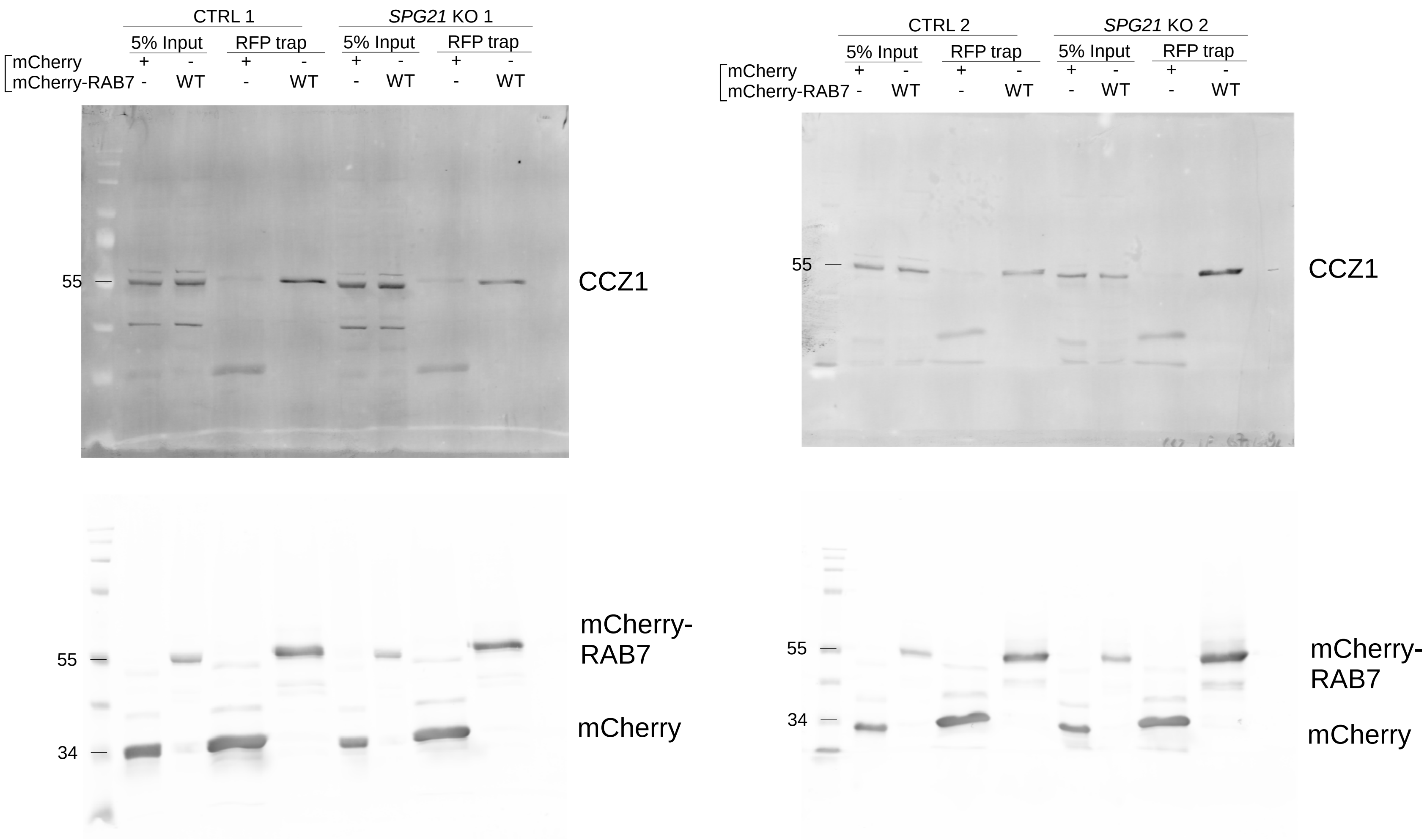

Figure S3E

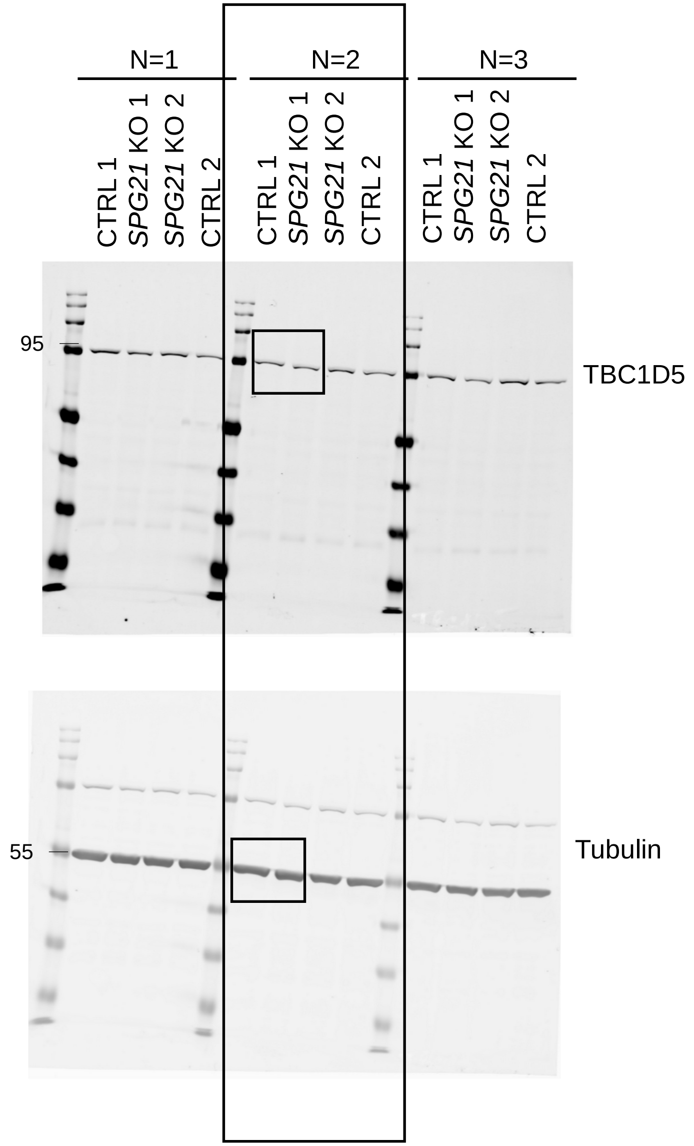

Data shown in the article extracted from this set

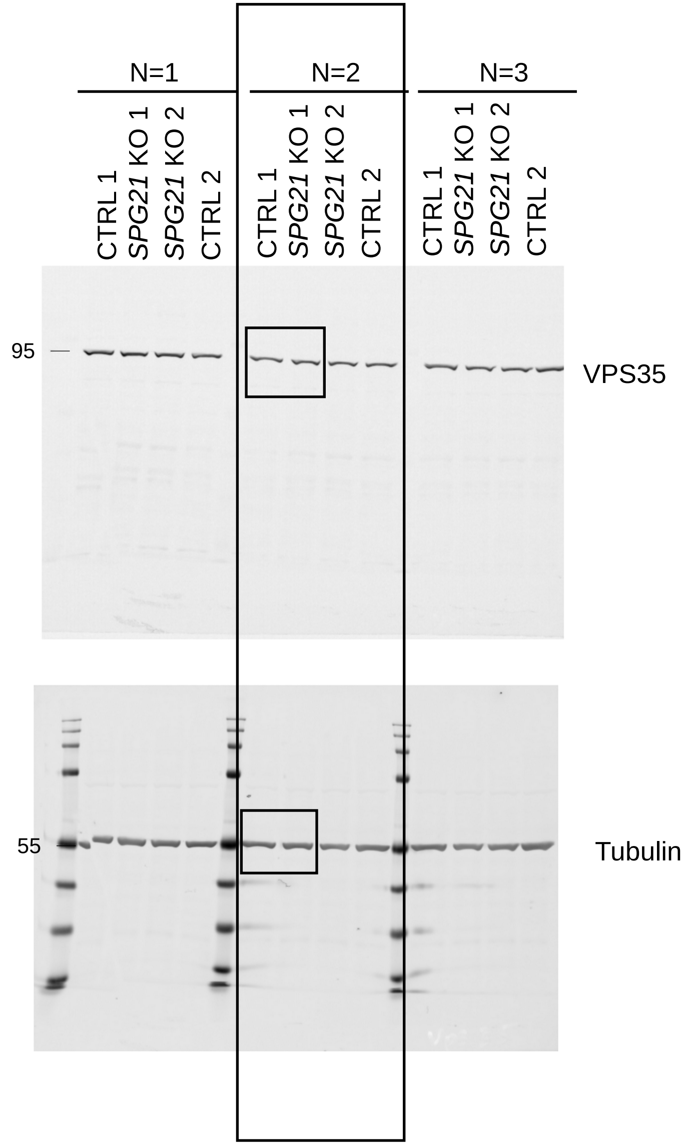

Data shown in the article extracted from this set
